# Cross-study safety analysis of risk factors in CAR T cell clinical trials: An FDA database pilot project

**DOI:** 10.1016/j.omto.2022.10.006

**Published:** 2022-10-20

**Authors:** Matthew Foster, Yonatan Negash, Leslie Eberhardt, Wilson W. Bryan, Kimberly Schultz, Xiaofei Wang, Yuan Xu, Bindu George

**Affiliations:** 1 Science Applications International Corporation (SAIC), Reston, VA 20190, USA; 2 Office of Tissues and Advanced Therapies (OTAT), Center for Biologics Evaluation and Research (CBER), U.S. Food and Drug Administration (FDA), Silver Spring, MD 20993, USA; 3 Office of Translational Sciences (OTS), Center for Drug Evaluation and Research (CDER), U.S. Food and Drug Administration (FDA), Silver Spring, MD 20993, USA

**Keywords:** chimeric antigen receptor (CAR) T cells, cytokine release syndrome, neurological toxicity, CD19, BCMA

## Abstract

The Chimeric Antigen Receptor (CAR) T Cell Safety Database Project explored the use of cross-study safety data to identify risk factors associated with severe cytokine release syndrome (sCRS) and severe neurological toxicities (sNTX) after CAR T cell administration. Sponsors voluntarily submitted data for 1,926 subjects from 17 phases 1 and 2 studies (six acute lymphocytic leukemia [ALL], five non-Hodgkin’s lymphoma [NHL], and six multiple myeloma [MM] studies). Subjects with ALL had a higher risk for developing sCRS and sNTX compared with subjects with NHL or MM. Subjects who received CAR T cells produced with gammaretrovirus vectors including CD28 sequences had higher rates of sNTX compared with subjects who received products produced with other vector designs included in the database. Use of cytokine-directed therapies and corticosteroids at lower toxicity grades were associated with lower rates of sCRS. Although this exploratory study was limited by unadjusted cross-study comparisons, it independently reproduced known risk factors for CAR T cell toxicity. Findings provide stakeholders in the CAR T cell clinical development community information on safety trends for consideration in early phase clinical trial design, as well as avenues for additional research.

## Graphical abstract

**Figure:**
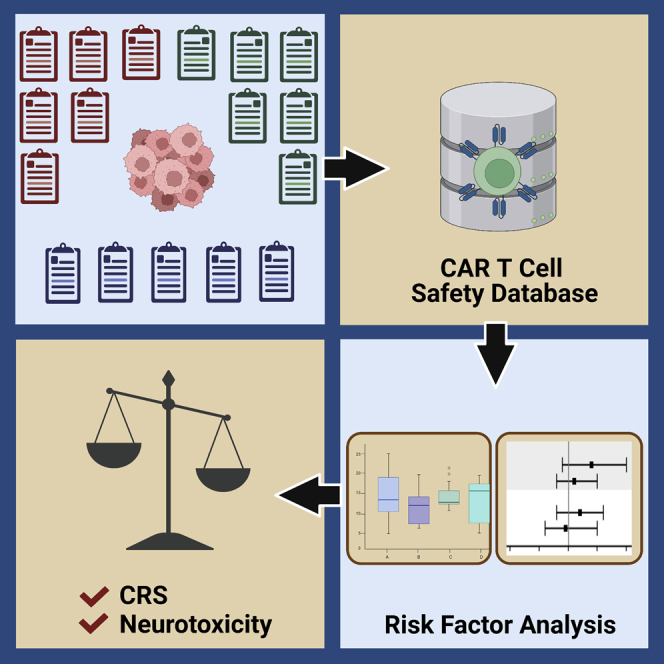

## Teaser

An exploratory safety analysis of chimeric antigen receptor (CAR) T cell products identified multiple risk factors associated with the adverse events: cytokine release syndrome and neurotoxicity. The findings inform stakeholders of potential risk factors associated with CAR T cell products and provide opportunities for future research.

## Introduction

Despite the approval of six chimeric antigen receptor (CAR) T cell products, cytokine release syndrome (CRS), a cytokine-mediated systemic inflammatory response, and neurological toxicities (NTX) continue to be life-threatening adverse events associated with CAR T cell administration.^1^^,^^2^^,^^3^^,^^4^^,^^5^^,^^6^^,^^7^ The clinical presentation of CRS varies from mild flu-like symptoms to high fevers, sinus tachycardia, hypotension, hypoxia, depressed cardiac function, and other signs of organ dysfunction, while NTX presents as a cluster of neurological symptoms ranging from mild confusion, headaches, and hallucinations to aphasia, seizures, and somnolence.^8^ CRS pathophysiology is postulated to be related to CAR T cell activation, expansion, and recruitment of other immune mediator cells, resulting in cascading cytokine production. Although the pathophysiology of NTX remains unknown, the leading hypothesis is a cytokine-mediated disruption of the blood-brain barrier.^9^ Symptoms of CRS and NTX often occur concurrently, and groups such as the American Society for Transplantation and Cellular Therapy (ASTCT) have published guidelines to better harmonize the definitions and grading systems for these adverse events.^10^ Treatment of CRS consists primarily of symptomatic treatment at lower toxicity grades but may involve the use of supplemental oxygen, high-dose vasopressor therapy, and tocilizumab (a humanized anti-human IL-6 receptor monoclonal antibody) with or without concomitant corticosteroid treatment for worsening CRS.^7^^,^^11^ Treatment strategies for NTX vary among different centers but consist primarily of corticosteroid therapy with or without cytokine-directed therapies and supportive care.

Many groups have attempted to identify risk factors associated with the development of CRS and NTX after CAR T cell product administration. Teachey et al.^12^ measured cytokines and clinical biomarkers in 51 subjects and found that peak levels of 24 cytokines (including IFN-ɣ, IL-6, sgp130, and sIL-6R) in the first month after infusion were highly associated with severe CRS. In addition, they developed multiple predictive models, including a logistic regression model, which used a combination of IFN-ɣ and CCL3 to predict which subjects would develop severe CRS (sensitivity 82%, specificity 93%). Hay et al.^13^ performed multivariate analysis of baseline characteristics in 133 adult subjects who received CD19 CAR T cells and identified high marrow tumor burden, lymphodepletion using fludarabine and cyclophosphamide, higher CAR T cell dose, thrombocytopenia before lymphodepletion, and manufacturing of CAR T cells without selection of CD8^+^ central memory T cells as independent predictors of CRS. Brudno and Kochenderfer^14^ performed a review of factors contributing to CRS and NTX and found that higher peak *in vivo* proliferation of CAR T cells, higher cell doses, conditioning chemotherapy containing fludarabine, acute lymphocytic leukemia (ALL) rather than non-Hodgkin’s lymphoma (NHL), higher burden of disease, baseline thrombocytopenia, and baseline elevated markers of endothelial activation (e.g., angiopoietin-2 and von Willebrand factor) were all risk factors for both CRS and NTX. In addition, they found that the CAR structure may contribute to patterns of toxicity. Tedesco and Mohan^15^ performed a systematic review of 33 CAR T cell clinical trials to identify biomarkers predictive of post-treatment CRS and NTX and found that circulating IL-6, IFN-ɣ, IL-10, and IL-15 appear to be associated with the severity of CAR T cell therapy toxicities in both leukemia and lymphoma subjects. Greenbaum et al.^16^ found that endothelial activation and stress index, a clinical surrogate for endothelial dysfunction, combined with ferritin and C-reactive protein was associated with both the incidence and severity of CRS and immune effector cell-associated neurotoxicity syndrome (ICANS) in subjects treated with CAR T cell products.

Although academic researchers have made important contributions to the current understanding of risk factors associated with CAR T cell-induced CRS and NTX, many groups are limited by either relatively small sample sizes or limited access to patient-level data. In order to address these limitations and increase the statistical power to detect risk factors associated with CAR T cell product administration, the FDA Center for Biologics Evaluation and Research (CBER), Office of Tissues and Advanced Therapies (OTAT), initiated a project to (1) assess the feasibility of integrating cross-study CAR T cell product safety data into a central CAR T cell safety database and (2) perform exploratory analyses to validate the use of cross-study data for risk factor identification and predictive modeling.^17^ In this project, clinical safety and chemistry manufacturing and control (CMC) data from 17 studies were voluntarily provided by sponsors and incorporated into a CAR T cell safety database. Maximum CRS and NTX grades within 28 days of CAR T cell product administration were calculated for each subject, and rates of severe (toxicity grade ≥ 3) CRS and NTX were compared among various demographic, clinical, and manufacturing groups to identify risk factors associated with severe CRS and NTX after CAR T cell product administration.

## Results

The CAR T cell safety database contains data for 1,926 subjects from 17 studies received prior to September 1, 2020. Six studies were phase 1, five were phase 2, and six were phases 1 and 2. Six studies contained primarily ALL subjects, five studies contained NHL subjects, and six studies contained multiple myeloma (MM) subjects. Four studies contained primarily pediatric subjects, while 13 studies contained primarily adult subjects.

A total of 1,277 subjects received at least one administration of a CAR T cell product, while the remaining 649 subjects did not receive treatment because of failure to meet the sponsor’s study requirements. Of the 1,277 subjects, 963 (75.4%) received CD19-targeting products, while 314 (24.6%) received B cell maturation antigen (BCMA)-targeting products. Median treated patient count per study was 61 (interquartile range [IQR]: 29–93) across all studies, 49 (IQR: 29–70) for ALL studies, 92 (IQR: 19–182) for NHL studies, and 47 (IQR: 18–79) for MM studies.

**Figure 1:**
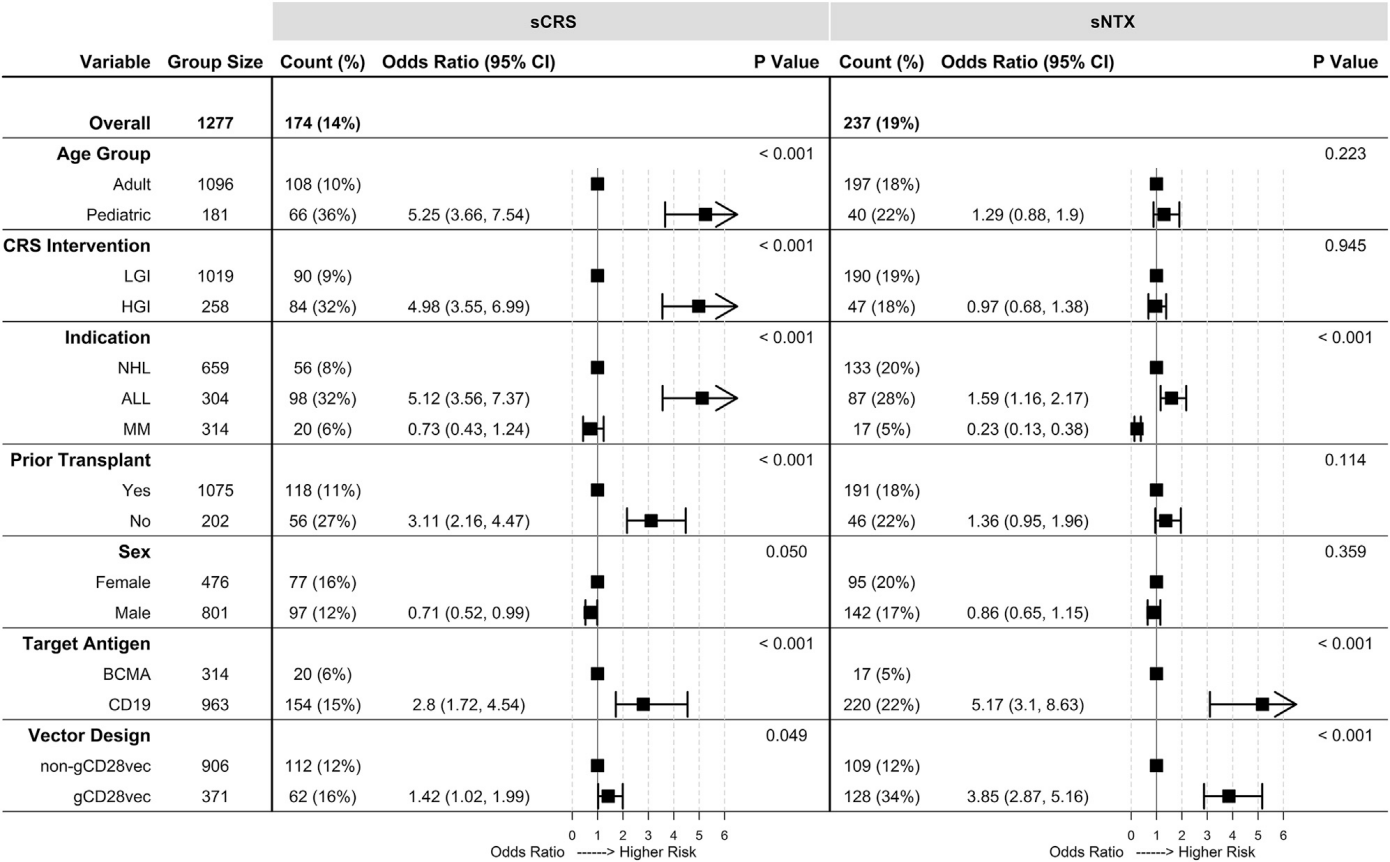
Pooled analysis for select clinical and manufacturing variables The first column lists select variables, while the second column lists the group size. For both severe cytokine release syndrome (sCRS) and severe neurological toxicities (sNTX), the “Count (%)” column gives the number of subjects who experienced sCRS/sNTX as well as the percentage relative to the group size. The “Odds Ratio (95% CI)” columns contain odds ratios and 95% confidence intervals calculated using the first variable in each group as a reference point (reference points: adult for age group, LGI for CRS intervention, NHL for indication, yes for prior transplantation, female for sex, BCMA for target antigen, and non-gCD28vec for vector design). Forest plots are displayed next to the “Odds Ratio (95% CI)” column to visually represent relative risk. The “P Value” column lists p values from chi-square tests comparing severe toxicity rates in each group. ALL, acute lymphocytic leukemia; NHL, non-Hodgkin’s lymphoma; MM, multiple myeloma; BCMA, B cell maturation antigen; gCD28vec, products produced with gammaretrovirus vectors with CD28 sequences in the transgene; LGI, low-grade intervention; HGI, high-grade intervention.

A summary table of group sizes, rates of severe CRS and NTX, and odds ratios comparing select variables from pooled analysis are shown in Figure 1. Further results for indication, CRS management protocol, vector design, age group, cytokines, expansion *in vivo*, dosing parameters, and CMC characteristics are reported below.

### CRS

Within the first 28 days after CAR T cell administration, 807 subjects (63.2%) experienced CRS and 174 subjects (13.6%) experienced severe CRS (sCRS) (toxicity grade ≥ 3). Median onset time for the first sCRS was 3.9 days post-infusion.

#### Indication

**Figure 2:**
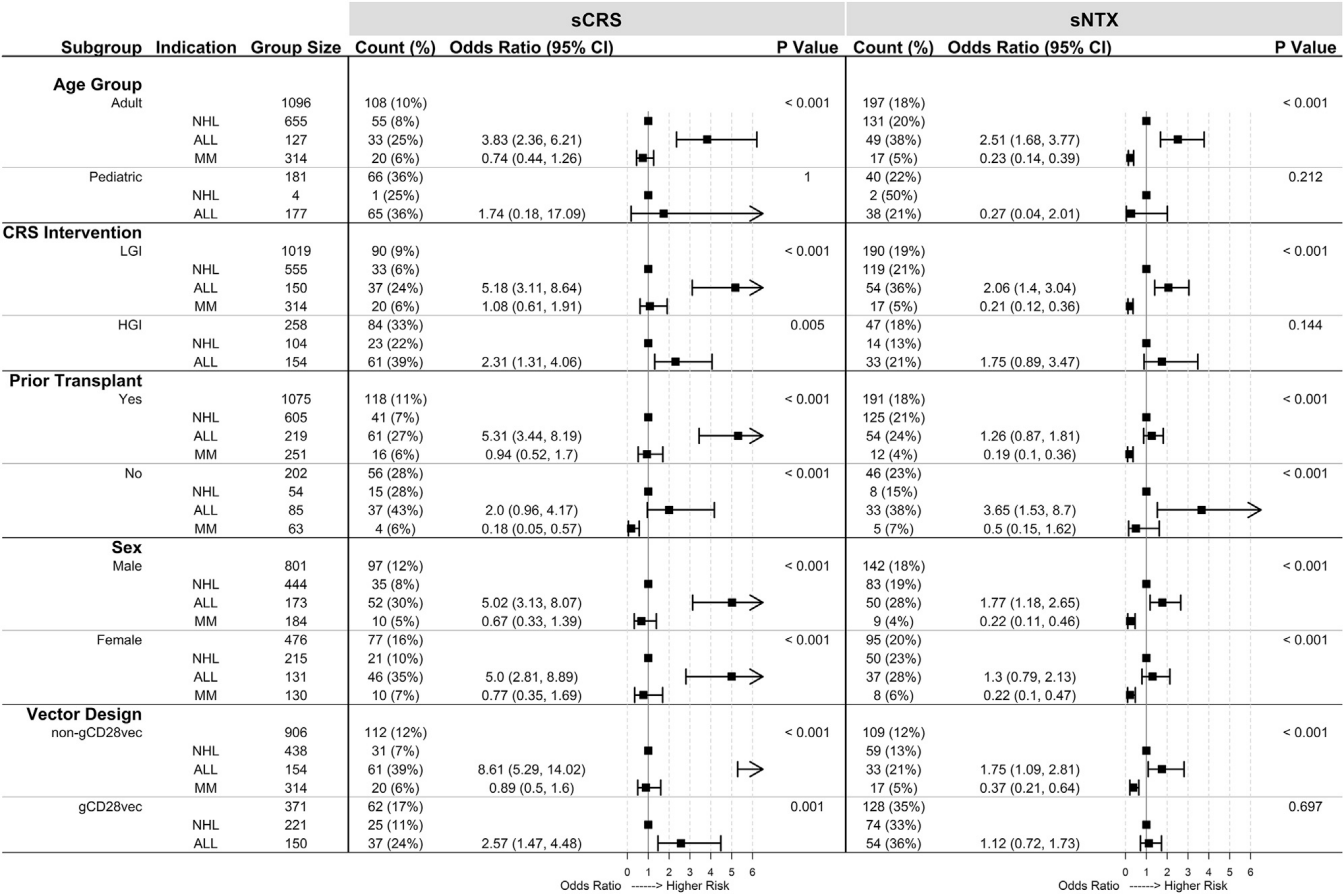
Analysis of indication within select subgroups Within each subgroup (as defined in the first column), rates of severe cytokine release syndrome (sCRS) and severe neurological toxicities (sNTX) were calculated for various indications (non-Hodgkin’s lymphoma [NHL], acute lymphocytic leukemia [ALL], and multiple myeloma [MM]) and presented in the “Count (%)” column. The “Odds Ratio (95% CI)” columns contain odds ratios and 95% confidence intervals calculated using NHL as a reference point within each subgroup. Forest plots are displayed next to the “Odds Ratio (95% CI)” column to visually represent relative risk. The “P Value” column lists p values from chi-square tests comparing severe toxicity rates in each group. gCD28vec, products produced with gammaretrovirus vectors with CD28 sequences in the transgene; LGI, low- grade intervention; HGI, high-grade intervention.

Of 1,277 treated subjects, 304 (23.8%) had ALL, 659 (51.6%) had NHL, and 314 (24.6%) had multiple myeloma. Subtypes of NHL were not analyzed. ALL subjects had higher rates of sCRS than NHL subjects, while there was no statistically significant difference between NHL and MM subjects in the frequency of sCRS (Figure 1, “Indication”). ALL subjects had higher rates of sCRS than NHL subjects in all subgroups except in subjects with no prior transplantation (Figure 2, “Prior Transplant: No”). An inadequate pediatric NHL sample size (n = 4) did not allow comparisons among pediatrics subjects (Figure 2, “Age Group: Pediatric: NHL”). For products administered to both adult ALL and adult NHL subjects, ALL subjects had higher rates of sCRS (Figure S1A). There was no statistically significant difference in sCRS rates between NHL and MM subjects (Figure 2), except in subjects with no prior transplantation (Figure 2, “Prior Transplant: No”).

#### CRS management protocol

**Figure 3:**
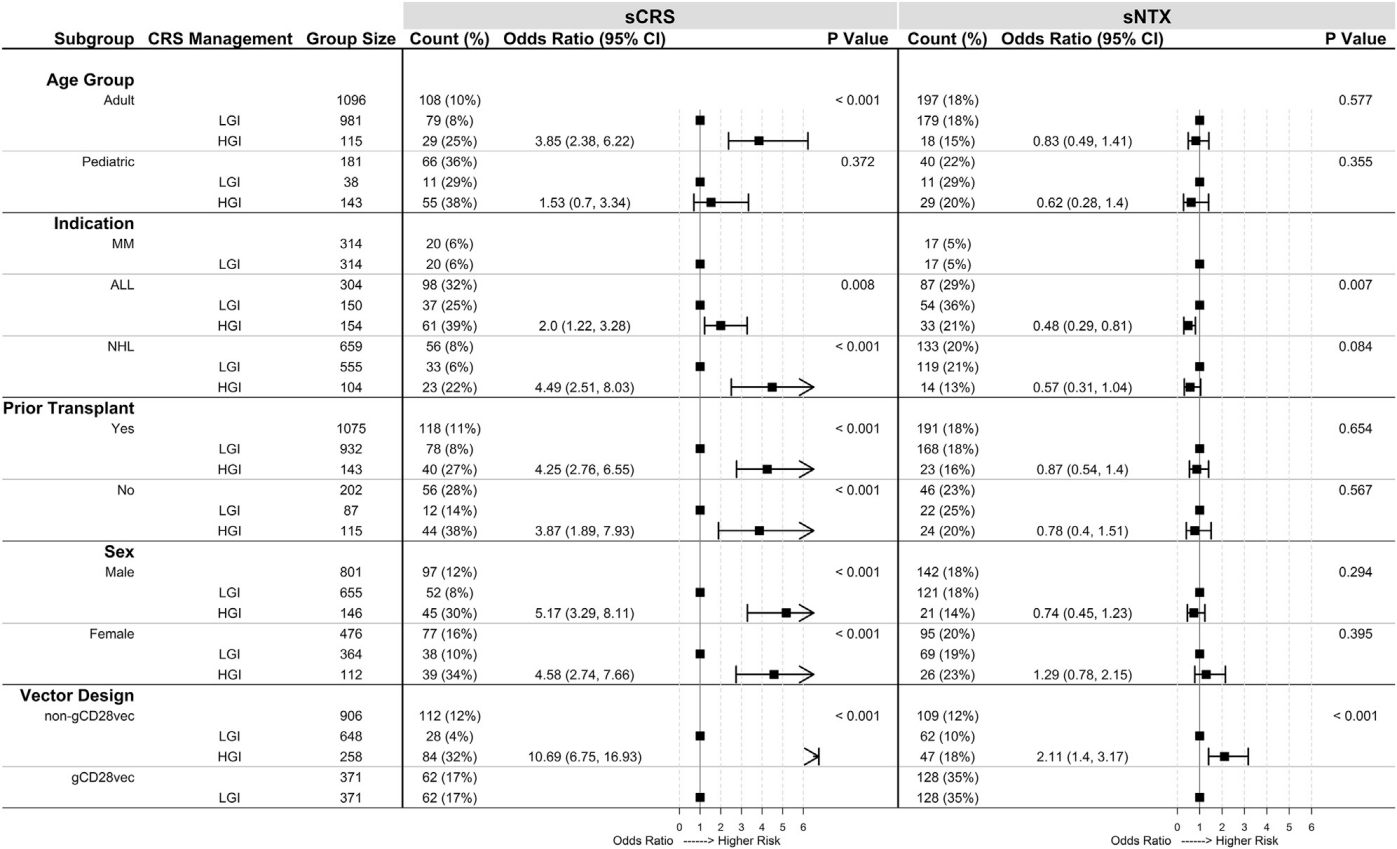
Analysis of CRS management protocol within select subgroups Within each subgroup (as defined in the first column), rates of severe cytokine release syndrome (sCRS) and severe neurological toxicities (sNTX) were calculated for the low-grade intervention (LGI) protocol and the high-grade intervention (HGI) protocol and presented in the “Count (%)” column. The “Odds Ratio (95% CI)” column contains odds ratios and 95% confidence intervals calculated using the LGI group as a reference point within each subgroup. Forest plots are displayed next to the “Odds Ratio (95% CI)” column to visually represent relative risk. The “P Value” column lists p values from chi-square tests comparing severe toxicity rates in each group. ALL, acute lymphocytic leukemia; NHL, non-Hodgkin’s lymphoma; MM, multiple myeloma; gCD28vec, products produced with gammaretrovirus vectors with CD28 sequences in the transgene.

CRS management protocols could be divided into two groups on the basis of the timing of intervention with tocilizumab. In the high-grade intervention (HGI) group, intervention with tocilizumab was generally reserved for sCRS while in the low-grade intervention (LGI) group, tocilizumab intervention could also be used as needed for grade 1 or 2 CRS. Only the rate of sCRS (using maximum CRS grade per subject) was considered in our analysis, not progression of CRS from lower to higher grades. HGI subjects experienced greater rates of sCRS than LGI subjects (Figure 1, “CRS Intervention”). Subgroup analyses supported this finding in all groups except pediatrics (Figure 3, “Age: Pediatric”). Because almost all pediatric subjects had ALL (n = 177 of 181), results from this subgroup analysis may not be applicable in other indications.

#### Vector design

**Figure 4:**
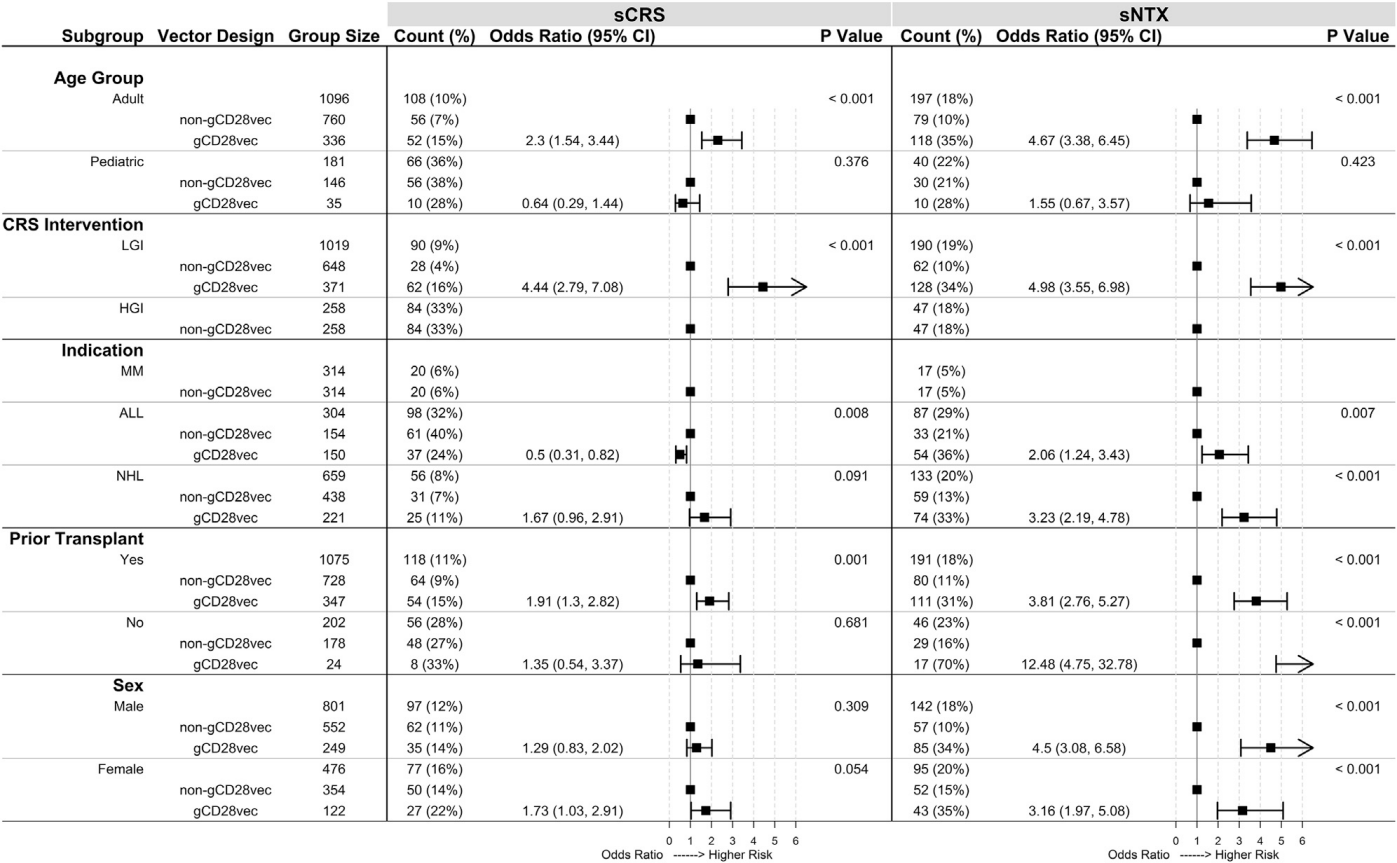
Analysis of vector design within select subgroups Within each subgroup (as defined in the first column), rates of severe cytokine release syndrome (sCRS) and severe neurological toxicities (sNTX) were calculated for products produced with gammaretrovirus vectors with CD28 sequences in the transgene (gCD28vec) and products not produced with this vector design (non-gCD28vec) and presented in the “Count (%)” column. The “Odds Ratio (95% CI)” column contains odds ratios and 95% confidence intervals calculated using the non-gCD28vec group as a reference point within each subgroup. Forest plots are displayed next to the “Odds Ratio (95% CI)” column to visually represent relative risk. The “P Value” column lists p values from chi-square tests comparing severe toxicity rates in each group. ALL, acute lymphocytic leukemia; NHL, non-Hodgkin’s lymphoma; MM, multiple myeloma; LGI, low-grade intervention; HGI, high-grade intervention.

Four different combinations of vector type (gammaretrovirus or lentivirus) and costimulatory domain (4-1BB or CD28) were used across 17 studies to manufacture CAR T cell products (Table S1). Although subjects receiving CAR T cells with gammaretrovirus vectors containing CD28 sequences (gCD28vec) had higher rates of sCRS (Figure 1, “Vector Design”), this result was inconclusive upon subgroup analysis (Figure 4).

#### Age group

Although pediatric subjects experienced higher rates of sCRS compared with adults (Figure 1, “Age Group”), this difference did not persist upon indication and CRS management protocol subgroup analyses (Figure S2A). As there were few pediatric subjects with NHL or MM (n = 4 of 181), subgroup analysis of age by indication could not be performed for these indications. Among ALL subjects, there was no statistically significant difference in sCRS rates between adults (26.0% [n = 33 of 127]) and pediatrics (36.7% [n = 65 of 177]) (p = 0.06). Further subgroup analysis of ALL subjects by CRS management protocol found no statistically significant difference in sCRS rates between pediatrics and adults in either the LGI (28.6% [n = 10 of 35] versus 23.5% [n = 27 of 115], p = 0.70) or the HGI (38.7% [n = 55 of 142] versus 50.0% [n = 6 of 12], p = 0.54) groups.

#### Cytokines

Of 116 cytokines/biomarkers in the CAR T cell safety database, there were data for 17 (IFN-ɣ, IL-1β, IL-2, IL-4, IL-5, IL-6, IL-7, IL-8, IL-10, IL-12, IL-13, IL-15, CCL2, CCL3, CCL4, GMCSF, and TNF-α) of these for at least 60% of treated subjects. Differences in cytokine levels between subjects who experienced sCRS and those who did not are reported in Tables 1 and S2 and Figure S3.

**Table 1:** Differences in cytokine levels for subjects with sCRS and sNTX

Cytokine	sCRS (36 h)	sCRS (before CRS)	sNTX (36 h)
CCL2	higher (p < 0.001, n = 582)	higher (p = 0.01, n = 787)	higher (p < 0.001, n = 582)
CCL3	NSD	NSD	lower (p < 0.001, n = 623)
CCL4	NSD	NSD	higher (p = 0.040, n = 627)
GMCSF	NSD	NSD	NSD
IFN-ɣ	higher (p = 0.01, n = 925)	higher (p = 0.03, n = 1,133)	NSD
IL-1β	NSD	NSD	NSD
IL-2	higher (p = 0.04, n = 913)	NSD	NSD
IL-4	lower (p < 0.001, n = 764)	lower (p < 0.001, n = 1,014)	higher (p = 0.014, n = 764)
IL-5	NSD	NSD	NSD
IL-6	NSD	NSD	NSD
IL-7	NSD	NSD	higher (p = 0.048, n = 600)
IL-8	higher (p = 0.001, n = 925)	NSD	higher (p = 0.023, n = 925)
IL-10	NSD	NSD	NSD
IL-12	NSD	lower (p < 0.001, n = 1,004)	NSD
IL-13	NSD	NSD	NSD
IL-15	NSD	NSD	NSD
TNF-α	NSD	NSD	NSD

Average maximum cytokines levels within 36 h after CAR T cell product administration in subjects with severe cytokine release syndrome (sCRS) and severe neurological toxicities (sNTX) compared with subjects with non-sCRS/non-sNTX. For subjects who experienced sCRS, average maximum cytokine concentrations were also calculated using all cytokine concentrations before CRS. NSD, no significant difference at p = 0.05.

#### Expansion *in vivo*

Higher peak CAR T cell expansion has been shown to be associated with CRS.^18^^,^^19^ Expansion *in vivo* was evaluated as the peak level (Cmax) of CAR T cell transgene copies in blood within 28 days after CAR T cell product administration. Data were available for 8 of 17 studies and 661 of 1,277 treated subjects. Median maximum transgene concentration was greater in subjects who experienced sCRS (57,988 versus 19,260 transgene copies/μg DNA, p < 0.001). Median maximum transgene concentration was greatest for ALL subjects (50,679 transgene copies/μg DNA, n = 243), followed by NHL (15,870 transgene copies/μg DNA, n = 410) and MM (243 transgene copies/μg DNA, n = 8). Because of differences in measurement units, not all MM expansion data could be included in the analysis. Among ALL subjects, median maximum transgene concentration was higher in adult (62,532 transgene copies/μg DNA, n = 78) compared with pediatric (44,652 transgene copies/μg DNA, n = 243) subjects (p=0.004). However, in subgroup analysis, differences between adult and pediatric ALL subjects receiving products with the same costimulatory domain were not significant. Transgene concentration measurements during the first 36 h after CAR T cell administration were not available for most subjects and were therefore not examined.

#### Dosing parameters

Three dosing parameters (transduced cell count, transduction frequency, and total cell count) were analyzed for correlation with sCRS. Transduced cell count is the quantity of T cells in the final CAR T cell product that have been transduced with the CAR, transduction frequency is the ratio of transduced cells to total cells in the final CAR T cell product, and total cell count is the quantity of all cells in the final CAR T cell product. Although products within a study are manufactured to meet the sponsors’ specified transduced cell dose, transduction frequency may vary widely among products because of the transduction rate of the patient-specific lot.

**Figure 5:**
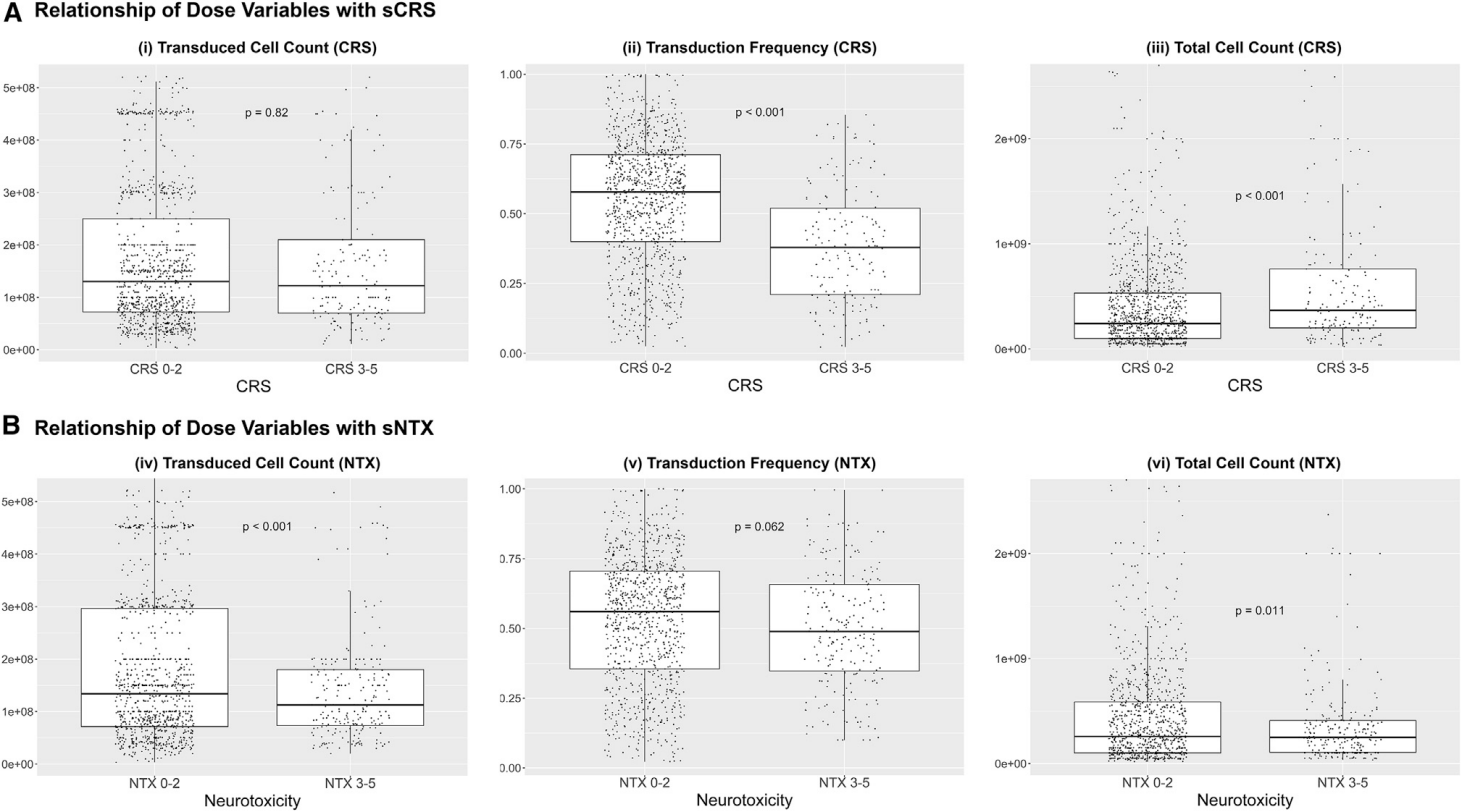
Relationship of dose variables with sCRS and sNTX (Ai) There was no statistically significant difference in median transduced cell count for products administered to subjects with and without severe cytokine release syndrome (sCRS) (1.2 × 10^8^ versus 1.3 × 10^8^, n = 1,165). (Aii) Subjects with sCRS received products with smaller median transduction frequency (37.9% versus 57.8%, n = 1,150). (Aiii) Subjects with sCRS received products with greater median total cell counts (3.7 × 10^8^ versus 2.4 × 10^8^, n = 1,254). (Biv) Subjects with severe neurological toxicities (sNTX) received products with smaller median transduced cell counts (1.1 × 10^8^ versus 1.3 × 10^8^, n = 1,165). (Bv) There was no statistically significant difference in median transduction frequency for products administered to subjects with versus without sNTX (56.1% versus 48.9%, n = 1,150). (Bvi) Subjects with sNTX received products with smaller median total cell counts (2.5 × 10^8^ versus 2.6 × 10^8^, n = 1,254). Results were considered significant at p ≤ 0.05.

There was no statistically significant difference in transduced cell counts between subjects who experienced sCRS versus non-sCRS (Figure 5Ai). Subjects who experienced sCRS received products with lower transduction frequencies (Figure 5Aii). Subgroup analysis by indication found that for subjects with ALL or NHL, but not MM, transduction frequencies were significantly lower among subjects who experienced sCRS (Figure S4). Subgroup analysis indicated that for LGI subjects, transduction frequencies were lower for those who experienced sCRS, while there was no statistically significant difference among HGI subjects (results not shown). Although subjects who experienced sCRS received products with greater total cell counts (Figure 5Aiii), this may be driven by subjects in the HGI group (which reported higher rates of sCRS) who received products with greater total cell counts than LGI subjects (7.90 × 10^8^ versus 1.92 × 10^8^, p < 0.001, n = 1,254). Subgroup analysis within CRS management protocols (LGI versus HGI) revealed no statistically significant difference in median total cell count between subject who experienced sCRS versus non-sCRS.

#### Percentage T cells

**Figure 6:**
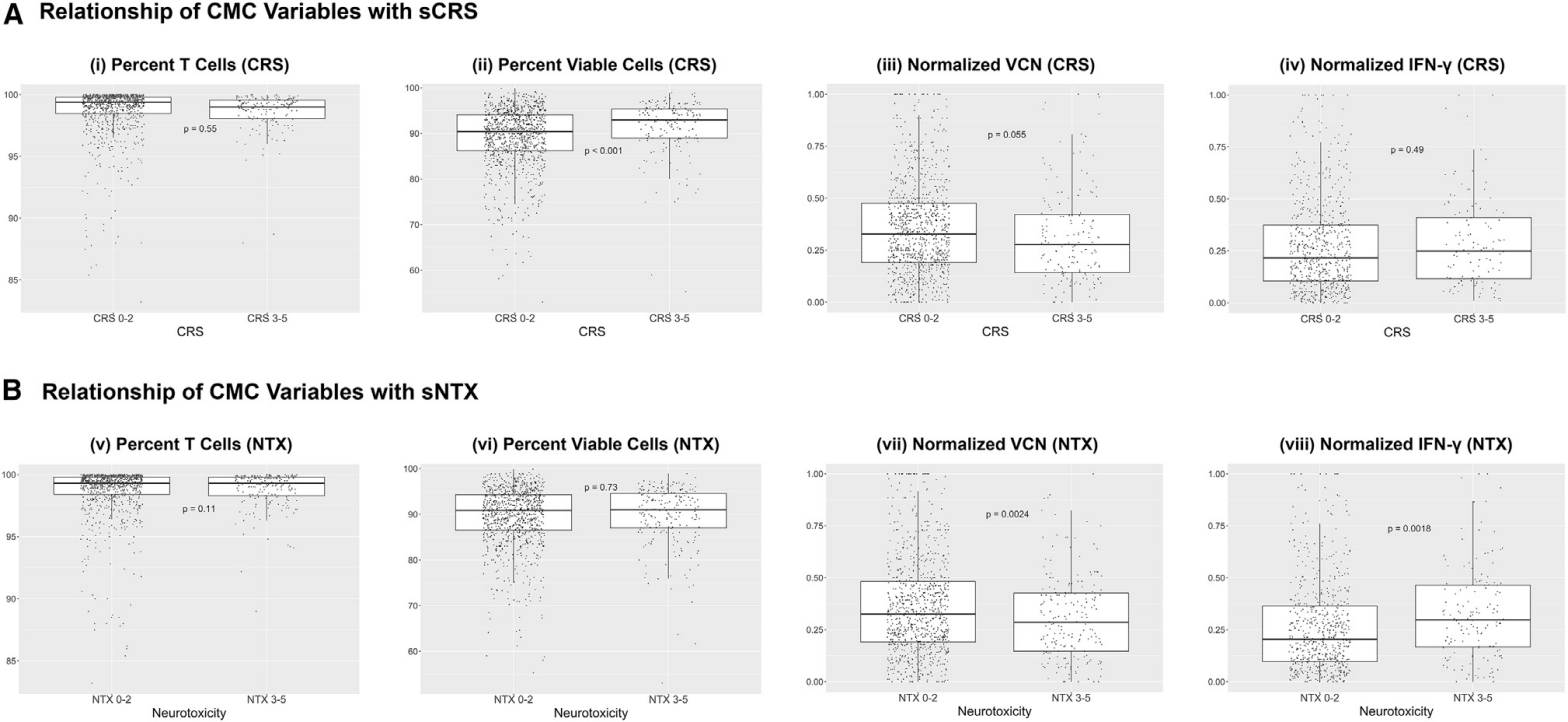
Relationship of cell manufacturing and control variables with sCRS and sNTX (Ai) Percentage T cells was not a differentiating factor for severe cytokine release syndrome (sCRS) (99.0% versus 99.4%, n = 1,047). (Aii) Subjects with sCRS received products with greater percentage viable cells (92.3% versus 90.4%, n = 1,149). (Aiii) Rank-normalized vector copy number (VCN) was not a differentiating factor for sCRS (0.28 versus 0.33, n = 1,061). (Aiv) Rank-normalized interferon gamma (IFN-ɣ) was not a differentiating factor for sCRS (0.25 versus 0.22, n = 745). (Bv) Percentage T cells was not a differentiating factor for severe neurological toxicities (sNTX) (99.3% versus 99.3%, n = 1,047). (Bvi) Percentage viable cells was not a differentiating factor for sNTX (91.0% versus 90.9%, n = 1,149). (Bvii) Subjects with sNTX received products produced with lower rank-normalized VCN (0.29 versus 0.32, n = 1,061). (Bviii) Subjects with sNTX received products with greater rank-normalized IFN-ɣ (0.30 versus 0.20, n = 745). Results were considered significant at p ≤ 0.05.

Percentage T cells was not a differentiating factor for sCRS (Figure 6Ai).

#### Percentage viable cells

Although cell viability was >90% for both groups, subjects who experienced sCRS received products with higher percentages of viable cells (Figure 6Aii). Subgroup analysis by indication revealed that among NHL subjects, percentages of viable cells were higher among subjects who experienced sCRS (94.5% [n = 52 of 493] versus 89.3% [n = 493 of 545], p < 0.001), whereas there was no statistically significant difference among subjects with ALL (92.2% [n = 97 of 298] versus 90.0% [n = 201 of 298], p = 0.16) or MM (92.8% [n = 18 of 292] versus 91.3% [n = 274 of 292], p = 0.97). Subgroup analysis by CRS management protocol found that among LGI subjects, subjects who experienced sCRS received products with a higher percentage of viable cells compared with subjects who did not experience sCRS (92.5% [n = 84 of 886] versus 89.8% [n = 802 of 886], p = 0.002). Figure S5 provides additional analysis of the relationship between cell viability and sCRS for both indication and CRS management protocol.

#### Vector copy number

Because of different underlying assay methods and measurement units (e.g., per cell, per transduced cell, per amount of DNA), vector copy number (VCN) data were rank-normalized; VCN levels in each study were assigned a value between 0 and 1. VCN rank was not a differentiating factor for sCRS (Figure 6Aiii).

#### Potency

Because of different underlying assay methods and measurement units, product potency results (as measured by IFN-ɣ secretion assay) were rank-normalized in each study by assigning a value between 0 and 1. Product potency rank was not a differentiating factor for sCRS (Figure 6Aiv).

### NTX

Within the first 28 days after CAR T cell administration, 851 subjects (66.6%) experienced NTX, and 237 subjects (18.6%) experienced severe NTX (sNTX) (toxicity grade ≥ 3). Median onset time for the first sNTX was 5.6 days post-infusion.

#### Indication

Compared with subjects with NHL, subjects with ALL had higher rates of sNTX, while subjects with MM had lower rates of sNTX (Figure 1, “Indication”). Subgroup analyses indicated that ALL subjects had increased risk of sNTX compared with NHL subjects in certain subgroups (adults, LGI CRS management protocol, no prior transplantation, males, and subjects receiving gCD28vec; Figure 2). There was no statistically significant difference in sNTX rate between adult ALL and NHL subjects administered the same CD19-targetting CAR T cell product (Figure S1B). MM subjects had lower rates of sNTX than NHL subjects (Figure 2), except in subjects with no prior transplantation (Figure 2, “Prior Transplant: No”).

#### CRS management protocol

There was no statistically significant difference in rates of sNTX between HGI and LGI subjects (Figure 1, “CRS Intervention”). Subgroup analysis supported this finding in all groups except ALL (Figure 3, “Indication: ALL”) and non-gCD28vec (Figure 3, “Vector Design: non-gCD28vec”). In the ALL subgroup, this was an artifact of HGI subjects’ consisting mostly of pediatrics who had lower rates of sNTX than adults (Figure S2B). Lower sNTX rates in the non-gCD28vec subgroup may be an artifact of LGI subjects’ receiving a disproportionate number of BCMA products, which had lower rates of sNTX than CD19 products (Figure 1). After controlling for age group and target antigen in the ALL group and non-CD28vec group, respectively, there were no statistically significant differences in sNTX rate between HGI and LGI subjects (Figures S6 and S7).

#### Vector design

Subjects in the gCD28vec group had higher rates of sNTX (Figure 1, “Vector Design”). Higher rates of sNTX were maintained regardless of sex, prior transplant status, CRS management protocol, or indication (Figure 4). Among adults, the gCD28vec group had higher rates of sNTX; while among pediatric subjects, there was no difference in sNTX rate between the gCD28vec and non-gCD28vec group (Figure 4, “Age Group”). As noted above, most pediatric subjects (n = 177 of 181) had underlying ALL, and the pediatric gCD28vec group was relatively small (n = 35), therefore we cannot rule out product- or indication-specific factors.

#### Age group

Although pooled analysis revealed no statistically significant difference in sNTX rates between adults and pediatrics (Figure 1, “Age Group”), subgroup analysis of ALL subjects indicated that pediatrics had lower rates of sNTX than adults (Figure S2B, “Indication: ALL”). Although some subgroups show pediatrics with higher rates of sNTX (Figure S2B, “Vector Design: non-gCD28vec”), this was secondary to indication as pediatrics primarily had ALL, which was associated with higher rates of sNTX (Figures 1 and 2). Controlling for indication in the non-gCD28vec group revealed pediatrics associated with lower rates of sNTX (19.7% [n = 28 of 142] versus 41.7% [n = 5 of 12]).

#### Cytokines

Differences in cytokine levels between subjects who experienced sNTX and those who did not are reported in Tables 1 and S2 and Figure S8.

#### Expansion *in vivo*

Median maximum transgene concentration was greater in subjects who experienced sNTX compared with those who did not (71,273 versus 19,997 transgene copies/μg DNA, p = 0.002).

#### Dosing parameters

Although subjects who experienced sNTX received products with lower transduced cell counts (Figure 5Biv), this finding did not persist in subgroup analysis by either indication or vector design. Transduction frequency was not a differentiating factor for sNTX (Figure 5Bv). Although subjects who experienced sNTX received products with lower total cell counts (Figure 5Bvi), this may be due to subjects in the gCD28vec group (which reported higher rates of sNTX) who received products with lower total cell counts than non-CD28vec subjects (2.36 × 10^8^ versus 3.00 × 10^8^, p < 0.001, n = 1,254). Subgroup analysis by vector design revealed no statistically significant difference in median total cell count between sNTX and non-sNTX subjects in either the gCD28vec or non-gCD28vec groups.

#### Percentage T cells

Percentage T cells was not a differentiating factor for sNTX (Figure 6Bv).

#### Percentage viable cells

Percentage of viable cells was not a differentiating factor for sNTX (Figure 6Bvi).

#### Vector copy number

Subjects who experienced sNTX received products manufactured with a lower rank VCN compared with subjects who did not experience sNTX (Figure 6Bvii). In subgroup analysis by indication, ALL subjects with sNTX received products manufactured with a lower rank VCN (0.21 [n = 84 of 288] versus 0.29 [n = 204 of 288], p = 0.002), whereas there was no statistically significant difference in VCN rank for subjects with NHL (0.33 [n = 103 of 508] versus 0.34 [n = 405 of 508], p = 0.61) or MM (0.31 [n = 12 of 265] versus 0.33 [n = 253 of 265], p = 0.34).

#### Potency

Although subjects who experienced sNTX received higher rank potency products (Figure 6Bviii), this finding did not persist in subgroup analysis by indication for subjects with ALL (0.3 [n = 46 of 175] versus 0.26 [n = 129 of 175], p = 0.32), NHL (0.3 [n = 88 of 329] versus 0.24 [n = 241 of 329], p = 0.09), or MM (0.17 [n = 11 of 241] versus 0.14 [n = 230 of 214], p = 0.45).

### Multivariate classification models

Multivariate models using data from all studies and all domains (clinical and CMC) were developed to identify which combination of factors show a strong association with the occurrence of sCRS or sNTX. Significant parameters for sCRS and sNTX are listed in Table 2. Forward-selected logistic regression models typically outperformed other classification methods on validation datasets. The best performing logistic regression model predicted the occurrence of sCRS with an accuracy of 73% and sensitivity of 80%, while the best model for predicting sNTX had an accuracy of 68% and sensitivity of 62%.

**Table 2:** Significant parameters for multivariate logistic regression models

Predictors	Odds ratio	95% CI	p value
**CRS (Grade 3–5)**			

Maximum temperature (36 h)	1.44	1.26–1.64	<0.001
Maximum IL-4 concentration (36 h)	0.93	0.88–0.97	0.004
ALL indication	3.01	1.69–5.49	<0.001
gCD28vec products	3.96	1.55–12.21	0.008
HGI CRS management protocol	5.35	1.95–17.27	0.002

**NTX (Grade 3–5)**			

Maximum temperature (36 h)	1.43	1.27–1.61	<0.001
MM indication	0.17	0.08–0.35	<0.001
gCD28vec products	2.93	1.90–4.56	<0.001

Multivariate models using data from all studies and all domains were developed to identify which combination of factors show a strong association with the occurrence of severe cytokine release syndrome (CRS) or neurological toxicities (NTX). Model parameters were selected using forward selection using cross validation scores from a logistic regression estimator and selected parameters were fit using logistic regression with L1 regularization. Odds ratios and associated p values for significant variables were obtained from a logistic regression estimator. For CRS, indication, CRS intervention, vector design, temperature, IL-4, IL-8, and TNF-α were selected during parameter selection step. Temperature, IL-4 concentration, acute lymphocytic leukemia (ALL) indication, products produced with gammaretrovirus vectors with CD28 sequences in the transgene domain (gCD28vec), and high-grade intervention (HGI) CRS management protocol had significant effects on risk for severe CRS. For NTX, indication, vector design, temperature, CCL2, IL-8, IL-1β, and potency (IFN-ɣ) were selected during parameter selection step. Temperature, multiple myeloma (MM) indication, and gCD28vec vector design had significant effects on risk for severe NTX.

## Discussion

The first goal of this project was to assess the feasibility of integrating CAR T cell product data from multiple studies to enable cross-study data analysis. Although data formats varied among sponsors, the creation of a CAR T cell product-specific standard format and the development of data extraction, transformation, and loading tools allowed successful integration of cross-study data into a single database.

The second goal of this project was to perform exploratory analyses to validate the use of the integrated database for risk factor identification and predictive modeling. Although subgroup analysis and normalization methods were used to adjust for differences between studies, not all sources of confounding were able to be controlled for. As a result, risk factors identified in this exploratory analysis may not be directly related to sCRS/sNTX but instead may reflect fundamental differences in the included phase 1 and 2 trials.^20^^,^^21^^,^^22^^,^^23^ In addition, as analyses were exploratory in nature, p values were not adjusted for multiple comparisons, further limiting the interpretation of results.^24^ Therefore, findings should be interpreted as hypothesis generating and are not intended to support clinical or regulatory decisions.

Despite these limitations, several findings will be of interest to the CAR T cell clinical development community and may be considered when designing early phase dose-escalation cohorts. First, adults with ALL had a higher risk for developing both sCRS and sNTX compared with adults with either NHL or MM. Although evidence for the association of ALL with sNTX was not as strong as that for sCRS, it was still evident in multiple subgroup analyses. Although the association of ALL with sCRS and sNTX may be related to CAR T cell expansion *in vivo* (ALL subjects had substantially higher maximum transgene levels after administration), exploration of this hypothesis was beyond the scope of this analysis. A limitation of this analysis is that although disease burden was captured in our database, there was no meaningful way to standardize disease burden across different indications. As disease burden has been associated with both sCRS and sNTX, differences in underlying disease burden may have confounded this result.^13^^,^^14^^,^^25^ Despite this limitation, our findings support work by others who have found that rates of sCRS are higher in patients with ALL than in those with NHL.^14^^,^^26^^,^^27^^,^^28^ Thus, when considering the design of phase 1 CAR T cell therapy studies (particularly for CD19-targeted products), separate dose-escalation cohorts based on indication may be considered for determining maximum tolerable dose, which may be different among different indications with the same target.

Second, among subjects with ALL, pediatric subjects were less likely to experience sNTX and equally likely to experience sCRS as adult subjects. This finding supports work by others who have compared rates of sCRS and sNTX in adult versus pediatric ALL subjects.^29^^,^^30^ Because of the limited number of pediatric subjects with NHL or MM in our database, it is unclear if these findings are applicable outside of ALL, although others have found age related associations with ICANS in adults with ALL and NHL.^31^^,^^32^^,^^33^^,^^34^

Third, studies using LGI with tocilizumab had lower rates of sCRS, with no difference in rates of sNTX. This finding supports the paradigm shift in CRS management that has been observed when comparing earlier studies with more recent ones, with the use of cytokine-directed therapies and corticosteroids at lower grades of toxicity leading to a significant reduction in sCRS with no evident impact on ICANS.^30^^,^^35^^,^^36^^,^^37^^,^^38^^,^^39^ These findings suggest different risk mitigation strategies may be needed for CRS and ICANS.

This study reports several CAR T cell CMC findings. First, CAR T cells produced with gammaretrovirus vectors that included CD28 sequences in the CAR design appeared to increase the risk for sNTX. This finding corroborates other who have noted that subjects receiving CAR T cell products with CD28 domains have experienced higher levels of sNTX.^14^^,^^30^^,^^40^ Because of the lack of structural variety in products produced using gammaretrovirus vectors, it was not possible to determine if the increased risk for sNTX was a result of vector type or CAR structure (Table S1). As vector design was typically uniform within a clinical study, the possibility of confounding factors unique to studies using this vector design cannot be ruled out.

Second, lower transduction frequency in the CAR T cell product was significantly associated with higher rates of sCRS and this relationship was observed in subjects grouped by both indication and study. This correlation could relate to the relationship between higher total cell counts and increased sCRS incidence, as lower transduction frequency would necessitate administration of a higher total number of cells to meet dose. This finding suggests that untransduced cells or cellular traits that contribute to lower transduction frequency could be a sCRS risk factor. Although others have found associations between T cell subset composition and toxicity, because neither the cellular composition of untransduced cells nor the T cell subsets were evaluated by most sponsors, we could not determine if particular cell types were associated with higher rates of sCRS.^13^^,^^41^^,^^42^^,^^43^^,^^44^^,^^45^

There were multiple challenges associated with interpreting results from this cross-study data analysis. The primary challenge was how to properly assess the trade-off between achieving greater statistical power for risk factor detection (by increasing the number of eligible subjects in the analysis) with the increase in potential confounders when combining datasets with different clinical and CMC profiles. Differences in the severity and frequency of CRS and NTX among subjects from different clinical and CMC subgroups (such as subjects with different cancers or different product profiles) may also have caused the analysis results to be confounded by factors unique to these subgroups. In addition, pertinent baseline characteristics such as tumor burden and inclusion/exclusion criteria across studies was not captured in our analysis and limits interpretation of results.

Although we tried to account for some sources of confounding by using subgroup analyses, differences in prognostic factors and limitations in the quantity of reported data between studies made it impossible to perform comparisons in a completely standardized way. Although disease burden data were sometimes available for leukemias, lymphomas, which require specifications for imaging techniques in addition to the standardized PET/CT-based imaging routinely used to evaluate response to therapy and disease status at study entry, were not available for most studies. Even if included, it would be difficult to interpret the results of an analysis evaluating the impact of disease burden on safety outcomes within and across indications, as differences in imaging parameters used to determine disease burden may also confound these results. Despite the limitations from confounding and missing data, this is the largest analysis of pooled data across CAR T cell products and results from this exploratory analysis can help researchers identify areas to test in more rigorously controlled populations.

A second major challenge was incorporating differences in grading and management strategies between studies. For CRS, differences in the Penn, Lee, and ASTCT criteria not only influence CRS toxicity grade but also the timing of tocilizumab administration, further complicating cross-study comparison.^10^^,^^46^^,^^47^^,^^48^^,^^49^^,^^50^ Multiple steps were taken to control for and investigate how differences in grading systems affected our findings. Subgroup analyses were performed within studies using the same grading systems to test the robustness of pooled results. We also repeated our analysis with sCRS defined as toxicity grade ≥ 4 (the grade at which different grading criteria converge in assessment) and found the primary findings of our analysis remained consistent (results not shown). We also investigated the algorithmic conversion between grading criteria and again found the primary findings of our analysis remained consistent (results not shown). These complementary approaches lend validity to our exploratory findings, despite variations in the CRS grading systems.

For NTX, outcomes were standardized using Medical Dictionary for Regulatory Activities (MedDRA) system organ classes. Despite not taking a more selective approach for classifying neurological events (e.g., using the ICANS definition), almost all studies used the same version of the Common Terminology Criteria for Adverse Events (CTCAE), and rates of sNTX were consistent with listed rates in the package insert of marketed products.^51^^,^^52^^,^^53^^,^^54^^,^^55^ Therefore this standardization strategy allowed accurate comparisons between studies in this exploratory analysis, although we do not recommend this approach for use in clinical trial design or decision making.

This study showed that although it is feasible to combine cross-study safety data for analysis, data standardization and differences in study design complicate analysis and interpretation of results. Although standardization efforts in the field may facilitate future efforts to assess safety risks across products, CAR T cell products are still an evolving therapeutic class and adherence to specified standards may not be feasible or desirable at this time. Although this study focuses specifically on risk factors associated with CRS and NTX, future analyses could consider additional safety factors and emerging toxicities (e.g., prolonged cytopenia, macrophage activation syndrome, or hemophagocytic lymphohistiocytosis).^56^^,^^57^ Findings from these exploratory analyses offer researchers and stakeholders in the CAR T cell clinical development community information on broader safety trends, considerations for early phase trial design, and avenues for future research.

## Materials and methods

Proprietary and confidential clinical safety and manufacturing data from 17 early to late phase studies received prior to September 1, 2020, were voluntarily provided by investigational new drug (IND) application sponsors for incorporation into the CAR T cell safety database.^17^ Studies were selected by asking sponsors with relatively large numbers of patients in CAR T cell trials to voluntarily contribute data for cross-study safety analysis. Such data, unlike submissions for licensing applications, were not cleaned or locked. Demographics, adverse events, concomitant medications, serum cytokine profiles, dosing regimen, cellular kinetic profiles, and CMC information were included in the CAR T cell safety database. Most data were provided in SAS transport files and followed the Clinical Data Interchange Standards Consortium (CDISC) Study Data Tabulation Model (SDTM).^58^ All data voluntarily submitted for this project were included in the analysis.

To integrate cross-study data into a standardized database, a custom data schema was designed based on the CDISC SDTM format. Software tools were developed to transform sponsor-submitted data to meet the requirements of the integrated database. As data were received, the initial data schema was updated to incorporate additional CAR T cell-specific information (e.g., cell population metrics). Reference tables were also developed to standardize terminology and reduce inconsistencies in the database. Additional information on data standardization (Notes S1–S5), as well as the final schema for the CAR T cell safety database (Appendix I), can be found in the supplemental information.

Occurrence of sCRS or sNTX within 28 days after CAR T cell product administration were the primary safety outcomes of interest. A time window of 28 days after product administration was selected to focus our analysis on short-term toxicities likely due to product administration and not related to disease progression. Subjects were classified as experiencing sCRS if they experienced at least one occurrence of CRS at toxicity grade ≥ 3 within 28 days after CAR T cell product administration. CRS toxicity grades were not reviewed for accuracy or adherence to the sponsor’s stated grading criteria and were accepted as reported. NTX was broadly defined as the occurrence of one or more adverse events under the neurologic disorders or psychiatric disorders Medical Dictionary for Regulatory Activities 20.1 system organ class.^59^ Subjects were classified as experiencing sNTX if they experienced at least one occurrence of NTX at toxicity grade ≥ 3 within 28 days after CAR T cell product administration. For subjects who had multiple episodes of CRS or NTX, only the maximum CRS or NTX toxicity grade was used for analysis. The most common adverse events used to define both NTX and sNTX are listed in Tables S3 and S4.

After integration of cross-study safety data, risk factors analysis was performed separately for sCRS and sNTX. Risk factors analyzed included indication (ALL versus NHL versus MM), CRS management protocol (HGI versus LGI; Note S6), vector design (gCD28vec versus non-gCD28vec), age group (adult versus pediatric; Note S7), cytokines (maximum concentration in blood within 36 h post-CAR T cell product infusion), expansion *in vivo* (maximum concentration of CAR T cell transgene copies in blood within 28 days post-CAR T cell product infusion), dosing parameters (transduced cell count, transduction frequency, and total cell count), and CMC characteristics (percentage T cells, percentage viable cells, vector copy number, and potency).

Bivariate analysis of continuous data was performed by splitting subjects into two groups on the basis of adverse event severity (i.e., severe versus non-severe) and using t tests to determine significant differences between groups (p ≤ 0.05). Bivariate analysis of categorical data used the chi-square test to test for differences in the percentages of subjects with sCRS or sNTX in different subgroups (p ≤ 0.05). For select variables, odds ratios and 95% confidence intervals were calculated to evaluate comparative risk. Initial bivariate analysis was followed by subgroup analyses to rule out potential confounding factors. For cases in which integration of numerical data from across studies was not possible, because of differences in units or underlying assay methods (e.g., if two studies used different target cells for IFN-ɣ secretion assays), data were either rank-normalized to values between 0 and 1 within each study or dropped from analysis. Despite the potential for different underlying assay methods, cytokine data were not rank normalized, as comparisons of cytokine distributions between studies were comparable after converting all measurements to picograms per milliliter.

Multivariate models such as logistic regression, decision trees, and random forests were developed to identify which combination of factors show a strong association with the occurrence of sCRS or sNTX. Inputs to these models included demographics data (including indication), CMC data (IFN-ɣ, VCN, cellular composition data, and vector design data), dose data (total cell count, transduced cell count, percentage transduction, total cell count per kilogram, and transduced cell count per kg), CRS management protocol, maximum cytokine concentrations within 36 h after CAR T cell administration, and maximum temperature within 36 h after CAR T cell administration (Note S8). All multivariate models were developed using training data and validated on data not used during model training. As cytokine data were typically submitted in a timeseries format with sparse measurements at irregular time points, cytokine data were interpolated to be compatible with multivariate analysis algorithms (Note S9).

## Data availability

The data that support the findings of this study are not openly available. Confidentiality agreements with sponsors who voluntarily provided clinical safety and manufacturing for this project prohibit data sharing.

## Supplemental information

Document S1. Notes S1–S9, Tables S1–S4, Figures S1–S8, and Appendix I

Document S2. Article plus supplemental information
