# Supplementary material for: Cross-study safety analysis of risk factors in CAR T cell clinical trials: An FDA database pilot project
Source: Mol Ther Oncolytics. 2022 Oct 20;27:182–94. doi: 10.1016/j.omto.2022.10.006 (PMC9643340; doi:10.1016/j.omto.2022.10.006)
Supplement: Document S1. Notes S1–S9, Tables S1–S4, Figures S1–S8, and Appendix I [file mmc1.pdf]

OMTO, Volume *27*

Supplemental information

Cross-study safety analysis of risk factors in CAR T cell clinical trials: An FDA database pilot project

Matthew Foster, Yonatan Negash, Leslie Eberhardt, Wilson W. Bryan, Kimberly Schultz, Xiaofei Wang, Yuan Xu, and Bindu George

Supplemental Materials

Supplemental Notes

Note S1: Data Standardization

Multiple reference tables were developed to standardize information across various domains, this included the adverse events, concomitant medications, disease identification, response grading, cell types, cellular markers, and cytokines/biomolecules reference tables. Further information on standardization efforts in the adverse events (Note S2) and concomitant medication (Note S3) domains can be found below.

Note S2: Adverse Event Data Standardization

Adverse event standardization required standardizing adverse event terms using the adverse event reference table, as well as labeling adverse events using an adverse event mapping dictionary. The Adverse Event reference table was built using the Medical Dictionary for Regulatory Activities (MedDRA) version 20.1 and allowed mapping between MedDRA preferred terms, lower-level terms, and system organ class entries in the adverse event domain. While a natural language processing (NLP) tool was initially developed to standardize adverse events to MedDRA preferred terms, we found that 98.8% of terms could be directly mapped using dictionary look up methods and therefore the added value of using NLP to improve dictionary mapping was offset by the time taken to perform these processes.

Note S3: Concomitant Medication Data Standardization

The concomitant medication table was built around the World Health Organization (WHO) B3 Drug Dictionary from March 2020 and allowed mapping between the WHO drug name, WHO Drug Code, reference ID, anatomic therapeutic class (ATC), chemical abstracts service (CAS) number, unique ingredient identifier (UNII), and WHO Standard Drug Groupings (SDG) identification number. This dictionary was chosen as it was the most used drug standardization dictionary by sponsors participating in our project. By standardizing treatment information with the WHO Drug dictionary, we were not only able to work with standardized data, but also utilize the ATC and SDG classification systems to analyze the effect of broader drug classes such as corticosteroids.

Note S4: Unit Testing for Data Quality Control

Unit testing occurred before parsing and served to test if there were any underlying problems with the data. Occasionally we ran into issues where a file was either corrupt or required special parameters to open and extract data. To address this, unit tests identified datafiles for which additional effort was needed to open the file. Other unit tests ensured that patient identification numbers (contained in the USUBJID column) in various SDTM domains occur in the patient demographics table. This was important as the demographics table served as a starting point during analysis and information was often merged into this table. If patients existed in other standard tables, but not in the patient demographics table, these data would be dropped during analysis and information would be lost. The results of each unit test were added to a text file which recorded the name of the test, the date and time the test was performed, and the input source of the unit test function. This log created a searchable record of problems with newly parsed data.

Note S5: Jupyter Notebooks for ETL Quality Control

Jupyter notebooks were used after parsing but before integration in the CAR T cell safety database. Parsing notebooks allowed the testing of data extract, transform, and load (ETL) functions and identified errors that required further data management solutions. For each domain, custom functions were written to perform tasks such as looking for outliers in dosing information, identifying spelling errors in adverse event or medication terms, and checking integrity of patient identifiers across data tables. Custom parsing functions were frequently developed within the parsing notebooks as well. For example, if regular expression substitution was required to clean dose information, a function to perform this action would first be developed within the Jupyter parsing notebook. This function would then be implemented into a parsing tools library to be called during parsing. By checking the final parsing result against the function output in the parsing notebook, we could identify issues in the function implementation process.

Other examples of information derived from the parsing notebooks were missing data problems, which could impact reference table creation when multiple source files were used. If a patient had multiple product identification numbers but only one administered product, this information would cause issues when tables were merged to create a single reference table. A decision could then be made on how to handle this issue (usually the patient was dropped). Another example of the missing data problem identified by the parsing notebooks was that different companies treat death differently. Usually, the demographics table would contain information about all patients, as well as if they died. The death details table however may only contain patients that died after receiving treatment. Differences like these could be identified via parsing notebooks and allowed us to capture a more complete clinical picture.

Note S6: CRS Management Protocol Group Notes

Selection into the lower grade (LGI) vs higher grade (HGI) grade intervention group was based on the management plan described in the protocol instead of based on utilizing tocilizumab administration data for each patient. To alleviate concerns that selection based on described protocol may not coincide with actual tocilizumab treatment, we evaluated tocilizumab use in low-grade CRS to ensure that a substantial proportion of HGI subjects did not receive tocilizumab at lower grades. Among subjects who only experienced grade 1 or 2 CRS (n=633), LGI subjects received tocilizumab at a relatively high rate (38.9%, n=211/542) while HGI subjects rarely received tocilizumab (9.9%, n=9/91). As only 9.9% of HGI subjects received tocilizumab at lower grade, we decided that selection of subjects into the HGI and LGI groups solely based on the study protocol requirements was an acceptable surrogate for our exploratory analysis.

Note S7: Age Group Definitions

Adults were defined as age ≥ 21 years of age while pediatrics were defined as age < 21. Age cut off was defined at 21 as early studies targeting pediatric subjects included young adults in this age range.

Note S8: Multivariate Model Development

Model parameters were selected using forward selection using cross validation scores from a logistic regression estimator and selected parameters were fit using Logistic Regression with L1 regularization. Odds ratios and associated p values for significant variables were obtained from a logistic regression estimator. Parameters selected during sCRS model development included Indication, CRS intervention, vector design, temperature, IL4, IL8 and TNFA. For sNTX model development, indication, vector design, temperature, CCL2, IL8, IL1β, and potency (IFN-ɣ) were selected during parameter selection step.

Note S9: Cytokine Interpolation

Three timeseries interpolation models were developed (a mixed effects model, a statistical model, and a neural network model) and evaluated on a subset of timeseries data not used during model development. The mixed effects model was a semi-mechanistic pharmacokinetic model based on an assumed model-structure of a rapid increase in cytokine levels, followed by a rapid decline, and then a slow decline.[1] The statistical model used the Expectation-Maximization with Bootstrapping algorithm to first perform longitudinal interpolation for each cytokine, followed by cross-sectional interpolation across all cytokines. The neural network model used a radial basis function network with two interpolation layers to generate regularly sampled timeseries data.[2] The mixed effects model was developed using the software tool NONMEM; the statistical interpolation model was developed using the Amelia Package in R; and the neural network model was developed using the TensorFlow package in Python.[3-5] Three metrics were used to measure interpolation model performance: the root-mean-square deviation (RMSD), the median absolute deviation (MAD), and the percentage of predictions within a 35% confidence interval of observed values. The best performing interpolation model was then used for predictive modeling.

Supplemental Tables

Table S1: *Rates of neurotoxicity and cytokine release syndrome by vector design.* CAR T cells produced with gammaretrovirus vectors that included CD28 sequences in the CAR design had higher risk of severe neurotoxicity (NTX Grade ≥ 3) sNTX but not severe cytokine release syndrome (CRS Grade ≥ 3). Grade ≤ 2 counts includes subjects with Grade 0 (did not experience CRS or NTX).

**Vector Design**				**Maximum NTX**		**Maximum CRS**	
**Costimulatory Domain**	**Transmembrane**	**Hinge**	**Vector Type**	**Grade ≤ 2**	**Grade ≥ 3**	**Grade ≤ 2**	**Grade ≥ 3**
CD28	CD28	CD28	Gamma-retrovirus	65.5% (n=243)	34.5% (n=128)	83.3% (n=309)	16.7% (n=62)
CD137 (4-1BB)	CD8a	CD8a	Lentivirus	88.1 % (n=446)	11.9% (n=60)	80.0% (n=405)	20.0% (n=101)
CD137 (4-1BB)	CD28	IgG4	Lentivirus	87.4% (n=341)	12.6% (n=49)	97.2% (n=379)	2.8% (n=11)
CD28	CD28	CD28	Lentivirus	100.0% (n=10)	0% (n=0)	100.0% (n=10)	0% (n=0)

Table S2: *Differences in Cytokine Levels for Subjects with sCRS and sNTX*. Average maximum cytokines levels in subjects with severe cytokine release syndrome (sCRS) or neurological toxicities (sNTX) compared to subjects without sCRS or sNTX. For subjects who experienced sCRS, average maximum cytokine concentrations were calculated using three different time ranges after CAR T cell administration (within 36 hours, before the occurrence of sCRS, and within 28 days). For subjects who experienced sNTX, average maximum cytokine concentrations were calculated using two different time ranges after CAR T cell administration (within 36 hours and within 28 days). NSD = No Significant Difference at p=0.05.

Cytokine	sCRS (36 hours)	sCRS (Before CRS)	sCRS (28 Days)	sNTX (36 hours)	sNTX (28 Days)
CCL2	Higher (p<0.001, n=582)	Higher (p=0.01, n=787)	Higher (p=0.039, n=823)	Higher (p<0.001, n=582)	NSD
CCL3	NSD	NSD	Higher (p=0.001, n=863)	Lower (p<0.001, n=623)	NSD
CCL4	NSD	NSD	Higher (p<0.001, n=865)	Higher (p=0.04, n=627)	NSD
GMCSF	NSD	NSD	Higher (p=0.036, n=923)	NSD	Higher (p=0.036, n=923)
IFN-γ	Higher (p=0.01, n=925)	Higher (p=0.03, n=1133)	Higher (p<0.001, n=1177)	NSD	NSD
IL1β	NSD	NSD	NSD	NSD	NSD
IL2	Higher (p=0.04, n=913)	NSD	Higher (p=0.005, n=1169)	NSD	NSD
IL4	Lower (p<0.001, n=764)	Lower (p<0.001, n=1014)	NSD	Higher (p=0.014, n=764)	NSD
IL5	NSD	NSD	NSD	NSD	NSD
IL6	NSD	NSD	Higher (p<0.001, n=1178)	NSD	NSD
IL7	NSD	NSD	Higher (p=0.002, n=843)	Higher (p=0.048, n=600)	Higher (p=0.002, n=843)
IL8	Higher (p=0.001, n=925)	NSD	Higher (p<0.001, n=1178)	Higher (p=0.023, n=925)	Higher (p=0.009, n=1178)
IL10	NSD	NSD	NSD	NSD	NSD
IL12	NSD	Lower (p<0.001, n=1004)	NSD	NSD	NSD
IL13	NSD	NSD	Higher (p=0.04, n=1038)	NSD	Lower (p=0.047, n=1038)
IL15	NSD	NSD	NSD	NSD	NSD
TNFα	NSD	NSD	NSD	NSD	NSD

Table S3: *Top 10 most frequent adverse events classified under neurotoxicity*. Includes all MedDRA 20.1 preferred terms (with toxicity grade ≥ 1) under the neurologic disorders or psychiatric disorders system organ class. Percentage calculated using the total number of patients who received at least one administration of a CAR T cell product (n=1,277).

MedDRA Preferred Term	Count	Frequency (n=1,277)
Headache	392	30.8 %
Confusional state	211	16.5 %
Encephalopathy	180	14.1 %
Tremor	173	13.5 %
Dizziness	145	11.4 %
Aphasia	111	8.7 %
Insomnia	91	7.1 %
Anxiety	77	6.0 %
Somnolence	71	5.6 %
Agitation	52	4.1 %

Table S4: *Top 10 most frequent adverse events classified under severe neurotoxicity*. Includes all MedDRA 20.1 preferred terms (with toxicity grade ≥ 3) under the neurologic disorders or psychiatric disorders system organ class. Percentage calculated using the total number of patients who received at least one administration of a CAR T cell product (n=1,277).

MedDRA Preferred Term	Count	Frequency (n=1,277)
Encephalopathy	113	8.8 %
Confusional state	41	3.2 %
Aphasia	36	2.8 %
Headache	22	1.7 %
Somnolence	20	1.6 %
Agitation	16	1.3 %
Mental status changes	15	1.2 %
Seizure	14	1.1 %
Delirium	14	1.1 %
Neurotoxicity	12	0.9 %

Supplemental Figures

(A) Risk of sCRS in ALL subjects compared to NHL subjects

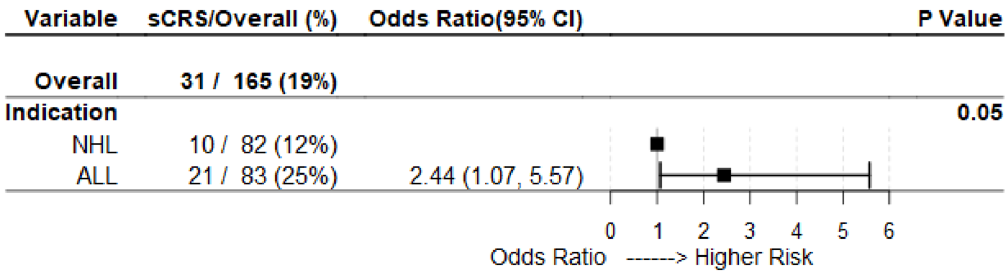

(B) Risk of sNTX in Adults with ALL vs NHL Given the Same Product

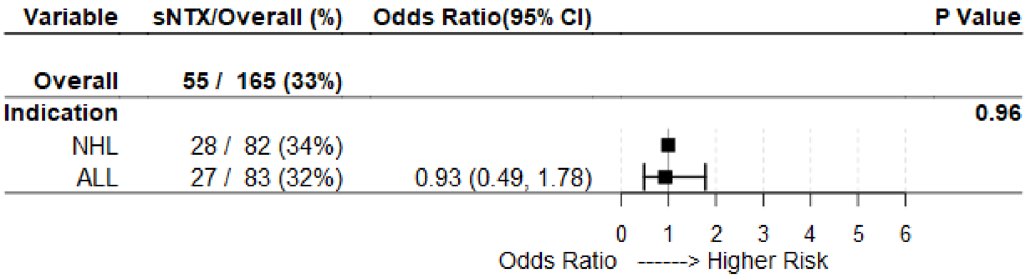

Figure S1: *Risk of sCRS and sNTX in adults with ALL vs NHL given the same product*. (A) Risk of severe CRS in ALL subjects compared to NHL subjects. (B) Risk of severe NTX in ALL subjects compared to NHL subjects. ALL = acute lymphocytic leukemia. NHL = non-Hodgkin's lymphoma. sCRS = severe (toxicity grade ≥ 3) cytokine release syndrome. sNTX = severe (toxicity grade ≥ 3) neurological toxicities.

(A) Subgroup Analysis of Age Group and sCRS

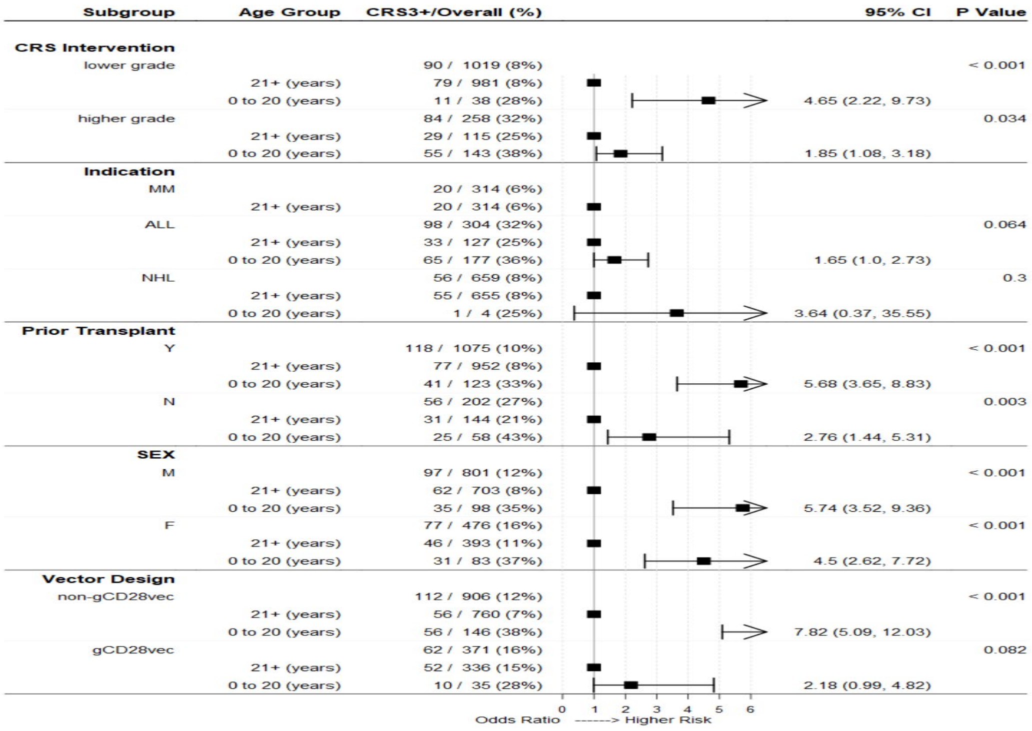

(B) Subgroup Analysis of Age Group and sNTX

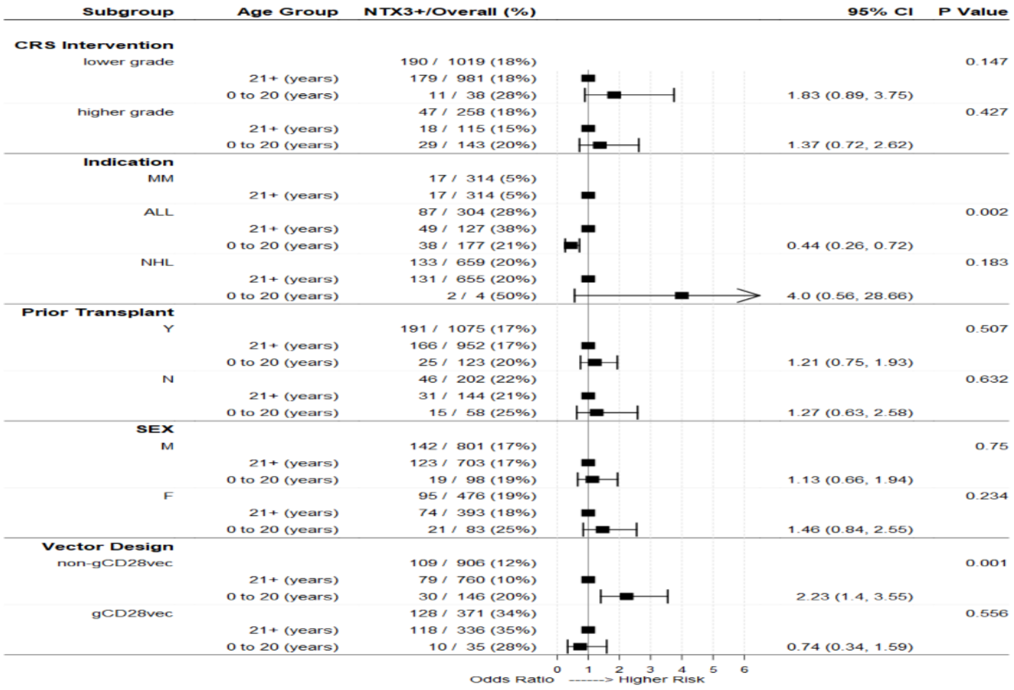

Figure S2: *Subgroup analysis of age group with sCRS and sNTX*. Within each subgroup (as defined in the first column), rates of severe (toxicity grade ≥ 3) cytokine release syndrome (sCRS) or neurological toxicities (sNTX) were calculated for adults (21+ (years)) and pediatrics (0 to 20 (years)). The fourth column contains an odds ratio and 95% confidence interval calculated using adults as a reference point within each subgroup. The fifth column contains forest plots to visually represent the odds ratio and 95% confidence interval. The sixth column contains p-values from chi-squared comparing severe toxicity rates in each group. (**A**) Subgroup analysis of age group and sCRS. (**B**) Subgroup analysis of age group and sNTX. ALL = acute lymphocytic leukemia. NHL = non-Hodgkin's lymphoma. MM = multiple myeloma. gCD28vec = products produced with gammaretroviral vectors with CD28 sequences in the transgene.

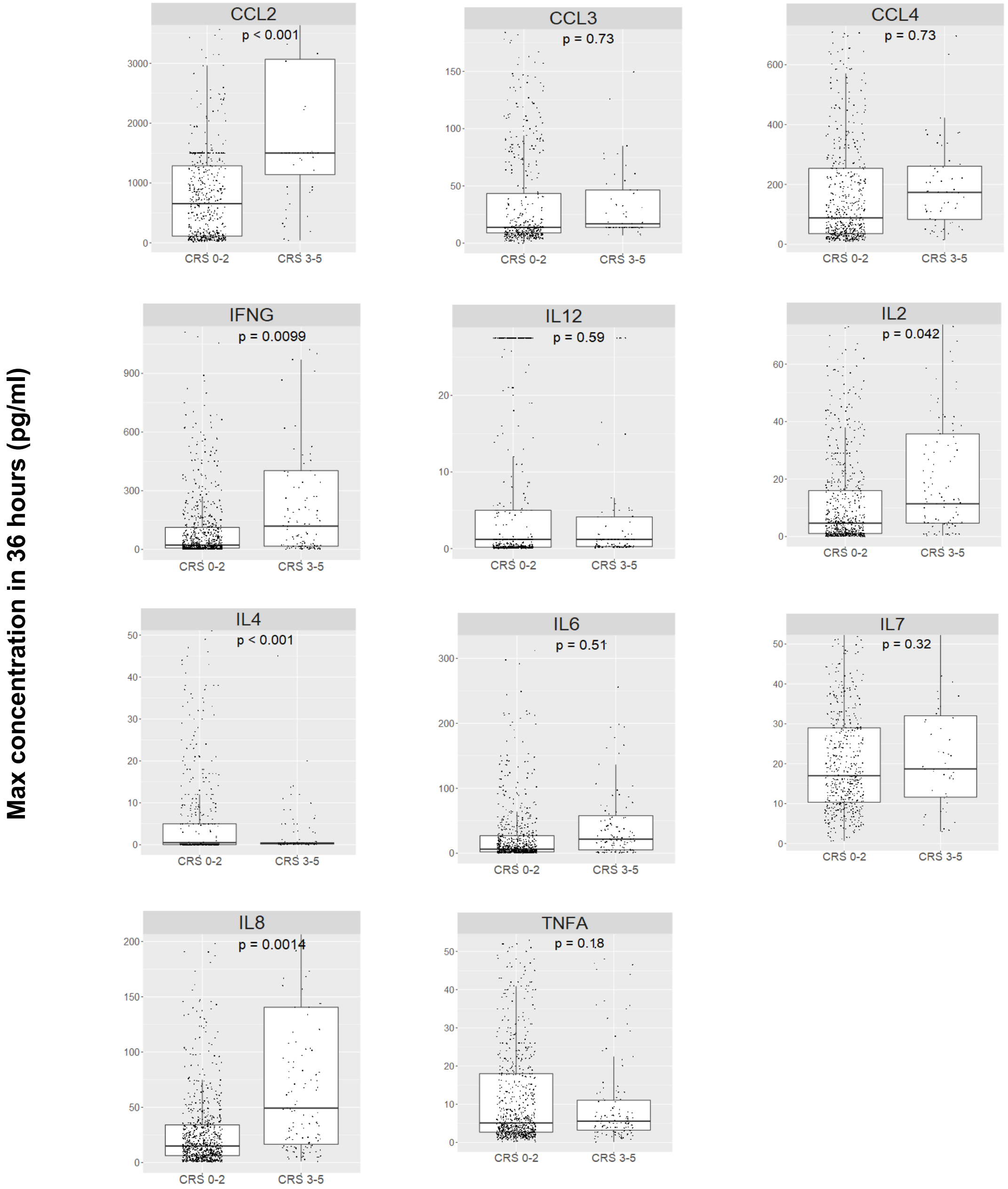

Figure S3: *Comparison of maximum cytokine concentrations between subjects with and without severe cytokine release syndrome*. Within 36 hours of CAR T cell product administration, subjects who experienced severe cytokine release syndrome (sCRS) had significantly higher maximum concentrations of IL2, CCL2, and IFN-γ and lower concentrations of IL4 compared with subjects with non-sCRS. CRS 0-2 = non-sCRS (cytokine release syndrome with toxicity grade 0, 1, or 2). CRS 3-5 = sCRS (cytokine release syndrome toxicity with toxicity grade 3, 4, or 5).

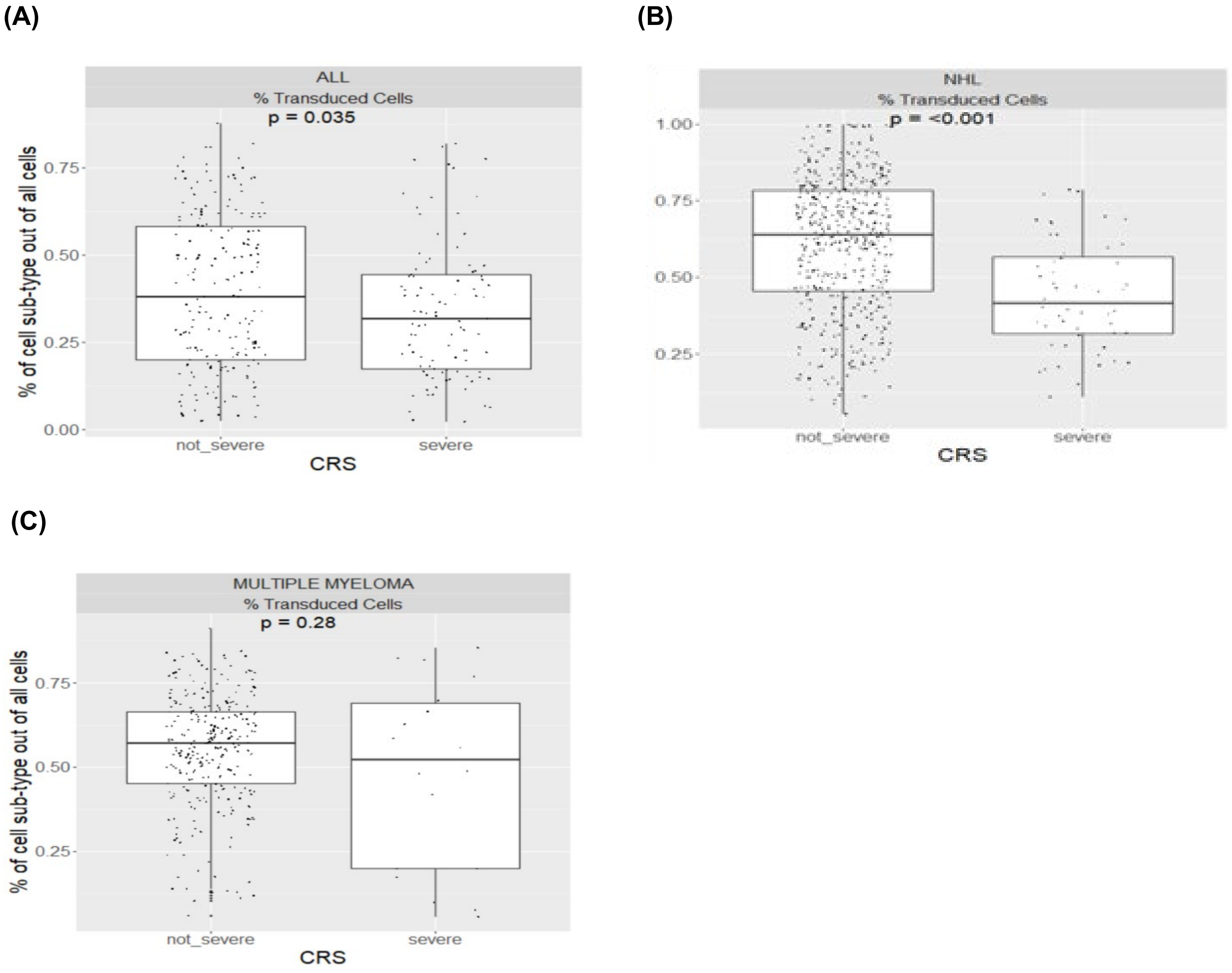

Figure S4: *Comparison of CAR T cell product transduction rates between subjects with and without sCRS by indication*. Transduction rates were lower among subjects who experienced severe (toxicity grade ≥ 3) cytokine release syndrome (sCRS) compared with subjects who did not for subjects with (A) ALL (32% vs. 38%, p=0.035, n=298) and (B) NHL (42% vs. 64%, p<0.001, n=546), but not for subjects with (C) Multiple Myeloma (52% vs. 57%, p=0.28, n=306). ALL = acute lymphocytic leukemia. NHL = non-Hodgkin’s lymphoma.

(A) Severe CRS Prediction Across Different Indications

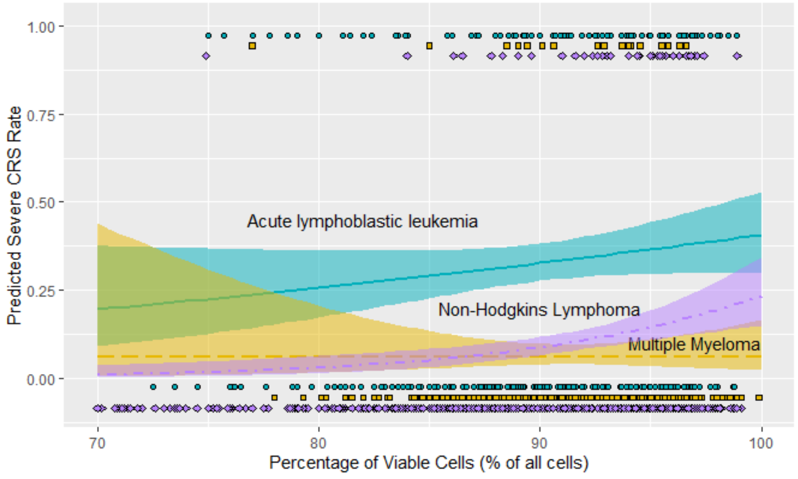

(B) Severe CRS Prediction in Different Intervention Strategies

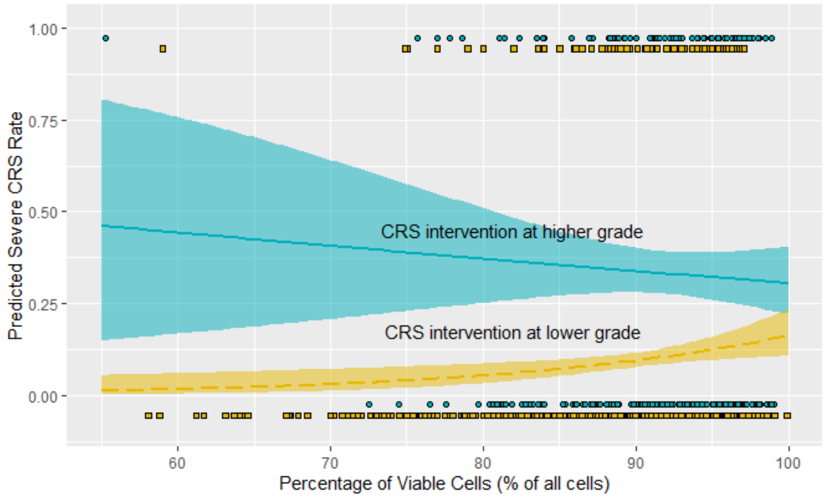

Figure S5: *Effect of Cellular composition of viable cells on the probability of sCRS*. (A) Subgroup analysis of probabilities of experiencing severe (toxicity grade ≥ 3) cytokine release syndrome (sCRS) at different levels of percentage of viable cells across different indication. Subgroup analysis by indication (underlying disease) found that among non-Hodgkin's lymphoma (NHL) subjects, percentages of viable cells were higher among subjects who experienced sCRS compared with subjects who did not (94.5%, n=52/493 vs. 89.3, n=493/545, p<0.001), although this difference was not significant in subjects with acute lymphocytic leukemia (ALL) (92.2%, n=97/298 vs. 90.0%, n=201/298, p=0.155) or multiple myeloma (MM) (92.8%, n=18/292 vs. 91.3%, n=274/292, p=0.974). (B) Subgroup analysis of probabilities of experiencing sCRS at different levels of percentage of Viable Cells across different CRS intervention groups. Subgroup analysis by CRS intervention groups found that among subjects in the lower grade intervention group, the percentage of viable cells was higher among subjects who experienced sCRS compared with subjects who did not (92.5%, n=84/886 vs. 89.8%, n=802/886, p=0.002).

Subgroup	CRS Intervention	sNTX/Overall (%)	Odds Ratio(95% CI)	P Value
**Age Group**				
Adult		49 / 127 (39%)		1
	LGI	44 / 115 (38%)		
	HGI	5 / 12 (41%)	1.15 (0.34, 3.86)	
Pediatric		38 / 177 (21%)		0.361
	LGI	10 / 35 (29%)		
	HGI	28 / 142 (19%)	0.61 (0.26, 1.42)	

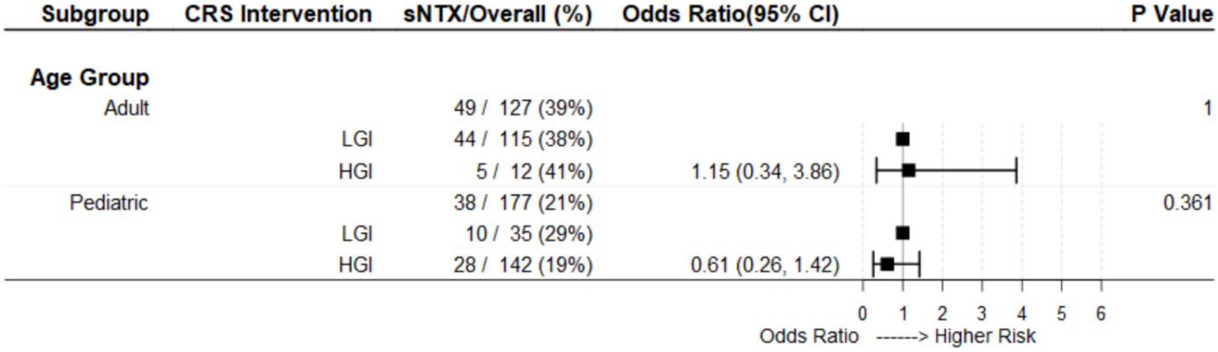

Figure S6: *Subgroup Analysis of CRS Intervention in Subjects with ALL*. For subjects with acute lymphocytic leukemia (ALL), there was no significant difference in severe (toxicity grade ≥ 3) neurological toxicity (sNTX) rates between higher grade and lower grade cytokine release syndrome (CRS) intervention strategies among either adults or pediatrics. HGI = higher grade intervention. LGI = lower grade intervention.

Subgroup	CRS Intervention	sNTX/Overall (%)	Odds Ratio(95% CI)	P Value
**Target Antigen**				
BCMA		17 / 314 (5%)		
	LGI	17 / 314 (5%)		
CD19		92 / 592 (16%)		0.143
	LGI	45 / 334 (13%)		
	HGI	47 / 258 (18%)	1.43 (0.92, 2.23)	

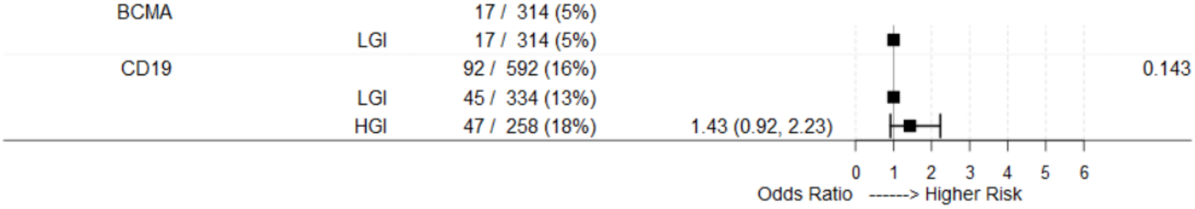

Figure S7: *Subgroup Analysis of CRS intervention in Subjects given non-gCD28vec products*. For subjects who did not receive CAR T cells with gammaretrovirus vectors containing CD28 sequences (non-gCD28vec), there was no significant difference in severe (toxicity grade ≥ 3) neurological toxicity (sNTX) rates between higher grade and lower grade cytokine release syndrome (CRS) intervention strategies among subjects who received CAR T cell products targeting CD19 antigens. For non-gCD28vec subjects, no subjects who received CAR T cell products targeting BCMA antigens were part of the higher grade intervention (HGI) group. LGI = lower grade intervention.

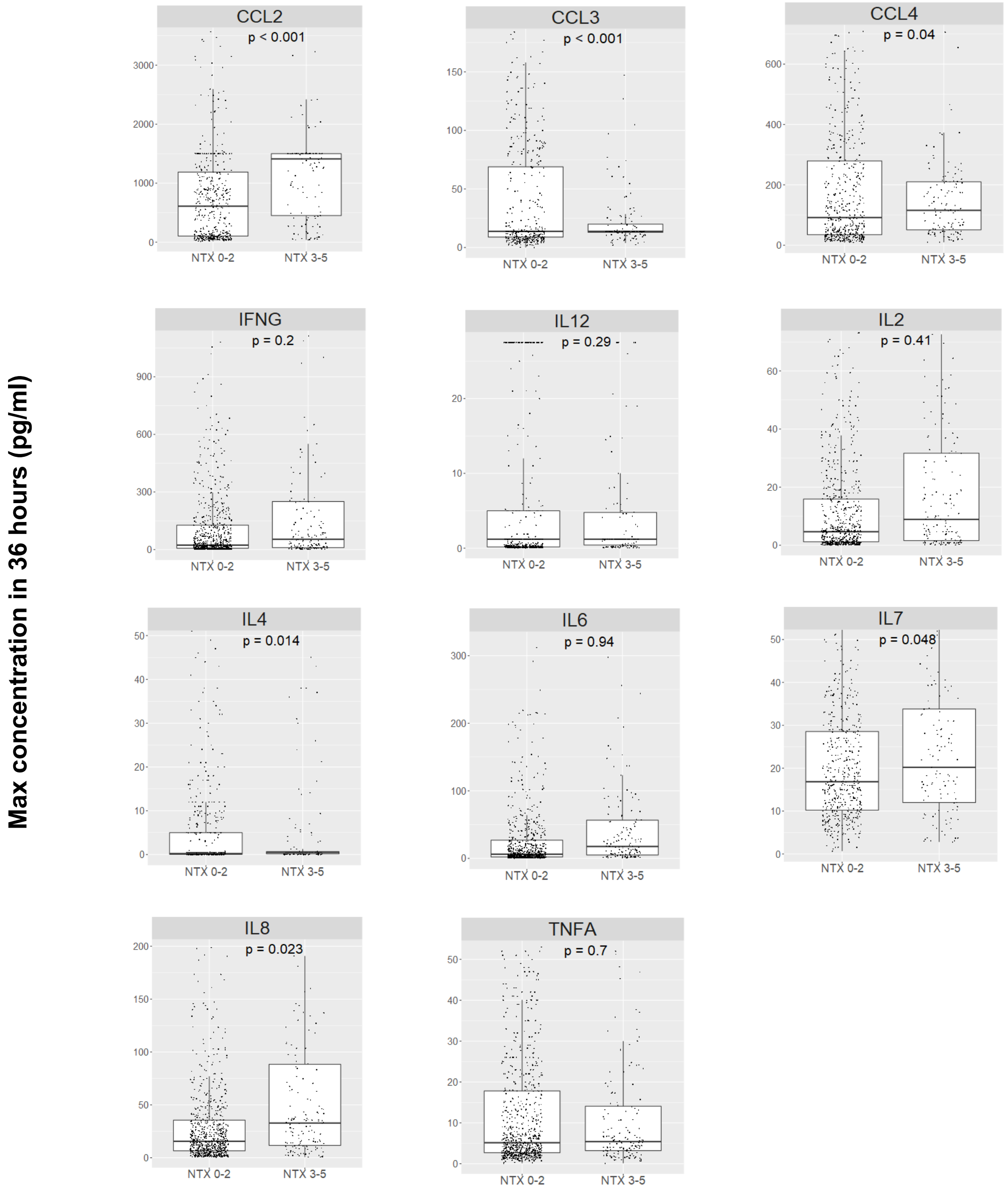

Figure S8: *Comparison of maximum cytokine concentrations between subjects with and without severe neurological toxicities***.** Within 36 hours of CAR T cell product administration, subjects who experienced severe neurological toxicities (sNTX) had significantly higher maximum concentrations of IL4, IL7, IL8, CCL2, and CCL4 and lower concentrations of CCL3 compared to subjects with non-sNTX. NTX 0-2 = non-sNTX (neurological toxicities with toxicity grade 0, 1, or 2). NTX 3-5 = sNTX (neurological toxicities with toxicity grade 3, 4, or 5).

Appendix I: CAR T Cell Data Schema

Demographics

Table Name: cart_demographics_adj

Description: Contains detailed patient information.

SDTM Domain: Demographics (DM)

Variable Name	Variable Label	Description
**USUBJID**	**Unique Subject Identifier**	Unique patient id.
**STUDYID**	**Study Identifier**	Unique study id.
**SITEID**	**Site Identifier**	Unique identifier for the site where the study occurs.
**INDID**	**IND identifier**	Investigational New Drug (IND) or Biologics License Application (BLA) number from which the data was parsed
**RFSTDTC**	**Date/Time of Initial Treatment**	CART infusion Start Date/Time. Use ISO 8601 format. All dates listed as YYYY-MM-DD T HH:MM: SS.
**RFENDTC**	**Date/Time when patient left study**	Date/Time when patient ended participation in the study. Use ISO 8601 format. All dates listed as YYYY-MM-DD T HH:MM: SS.
**BRTHDTC**	**Date/Time of Birth**	Date/Time of Birth of the subject. Use ISO 8601 format. All dates listed as YYYY-MM-DD T HH:MM: SS. Only year is parsed from patient data, all other info standardized to YYYY-01-01T00:00:00.
**AGE**	**Age**	Age of patient at the time of demographics data collection.
**AGEU**	**Age Units**	Unit for age.
**SEX**	**Sex**	Gender of patient. Use the following labels:<br>Label: F — Description: Female<br>Label: M — Description: Male
**RACE**	**Race**	Race of patient. Refer to "Collection of Race and Ethnicity Data in Clinical Trials. Guidance for Industry and Food and Drug Administration Staff" (FDA, October 2018) for guidance regarding the collection of race (https://www.fda.gov/media/75453/download).
**ETHNIC**	**Ethnicity**	Race of patient. Refer to "Collection of Race and Ethnicity Data in Clinical Trials. Guidance for Industry and Food and Drug Administration Staff" (FDA, October 2018) for guidance regarding the collection of ethnicity (https://www.fda.gov/media/75453/download).
**weight**	**Weight**	Initial weight of patient.
**weightu**	**Weight Units**	Unit for weight.
**COUNTRY**	**Country**	Country of study site.
**DMDTC**	**Date/Time of Collection**	Date of collection of demographics data.
**height**	**Height**	Initial height of patient.
**heightu**	**Height Units**	Unit for height.

<table>
<tr><td>prior_transplant</td><td>Prior Transplant</td><td>Has the patient received stem cell transplantation prior to treatment? Use the following labels:<table><tr><th>Label</th><th>Description</th></tr><tr><td>Y</td><td>Yes</td></tr><tr><td>N</td><td>No</td></tr></table></td></tr>
<tr><td>transplant_type</td><td>Transplant Type</td><td>Type of prior_transplant. Use the following labels:<table><tr><th>Label</th><th>Description</th></tr><tr><td>Autologous</td><td>Autologous</td></tr><tr><td>Allogeneic</td><td>Allogeneic</td></tr></table></td></tr>
</table>

Required entries:
Demographics information is required for all patients.

Non-Duplicate Key:
The following variables are used to ensure there are no duplicate data:

- USUBJID
- STUDYID

Additional Information: NA

Death Details

Table Name: DEATH_DETAILS

Description: Contains detailed information about death occurrences.

SDTM Domain: Death Details (DD)

<table>
<tr><th>Variable Name</th><th>Variable Label</th><th>Description</th></tr>
<tr><td>USUBJID</td><td>Unique Subject Identifier</td><td>Unique patient id.</td></tr>
<tr><td>STUDYID</td><td>Study Identifier</td><td>Unique study id.</td></tr>
<tr><td>SITEID</td><td>Site Identifier</td><td>Unique identifier for the site where the study occurs.</td></tr>
<tr><td>DDTESTCD</td><td>Death Detail Assessment Short Name</td><td>Name of assessment. See below for required values.
<table>
<tr><th>Label</th><th>Description</th></tr>
<tr><td>CAUSE_OF_DEATH</td><td>Text description of the cause of death.</td></tr>
<tr><td>AUTOPSY_FINDING</td><td>Text description of autopsy findings.</td></tr>
</table></td></tr>
<tr><td>DDTEST</td><td>Death Detail Assessment Name</td><td>Long name for assessment code found in ‘DDTESTCD’.</td></tr>
<tr><td>DDORRES</td><td>Result</td><td>Result of assessment.</td></tr>
<tr><td>DDDTC</td><td>Date/Time of Collection</td><td>Date/time of assessment. Use ISO 8601 format. All dates listed as YYYY-MM-DD T HH:MM: SS.</td></tr>
<tr><td>date_of_death</td><td>Date/Time of Death</td><td>Date/time when patient was declared dead. Use ISO 8601 format. All dates listed as YYYY-MM-DD T HH:MM: SS.</td></tr>
<tr><td>death_study_day</td><td>Study Day of Death</td><td>Study Day of death derived from the 'date_of_death' (preferred) or ‘DDDTC’ (if 'date_of_death' not available). Calculated by subtracting the date_of_death from ‘RFSTDTC’ variable in the Demographics table.</td></tr>
</table>

Required entries: NA

Non-Duplicate Key:
The following variables are used to ensure there are no duplicate data:

- USUBJID
- STUDYID
- SITEID
- DDTEST

Additional Information: NA

Disease Identification

Table Name: DISEASE_IDENTIFICATION

Description: Contains information about the patients underlying condition and date of diagnosis.

SDTM Domain: **Disease Response (RS)**

<table>
<tr><th>Variable Name</th><th>Variable Label</th><th>Description</th></tr>
<tr><td>USUBJID</td><td>Unique Subject Identifier</td><td>Unique patient id.</td></tr>
<tr><td>STUDYID</td><td>Study Identifier</td><td>Unique study id.</td></tr>
<tr><td>SITEID</td><td>Site Identifier</td><td>Unique identifier for the site where the study occurs.</td></tr>
<tr><td>indication</td><td>Indication</td><td>Disease for which patients are receiving CART treatment. Use the following labels:
<table>
<tr><th>Label</th><th>Description</th></tr>
<tr><td>ALL</td><td>Acute lymphoblastic leukemia</td></tr>
<tr><td>AML</td><td>Acute myeloid leukemia</td></tr>
<tr><td>CLL</td><td>Chronic lymphoid leukemia</td></tr>
<tr><td>CML</td><td>Chronic myeloid leukemia</td></tr>
<tr><td>HL</td><td>Hodgkin's lymphoma</td></tr>
<tr><td>MM</td><td>Multiple myeloma</td></tr>
<tr><td>NHL</td><td>Non-Hodgkin lymphoma</td></tr>
</table></td></tr>
<tr><td>indication_cat</td><td>Category of Indication</td><td>Used to identify different categories within a disease. See Additional Information for required categories.</td></tr>
<tr><td>indication_scat</td><td>Category of Indication</td><td>Subcategory used to identify different categories within a disease category. Use the following labels:
<table>
<tr><th>Label</th><th>Description</th></tr>
<tr><td>Relapsed</td><td>Relapsed</td></tr>
<tr><td>Refractory</td><td>Refractory</td></tr>
<tr><td>Relapsed/Refractory</td><td>Relapsed/Refractory</td></tr>
</table></td></tr>
<tr><td>diag_date</td><td>Date/Time of Diagnosis</td><td>Date time when diagnosis was made. Use ISO 8601 format. All dates listed as YYYY-MM-DD T HH:MM: SS.</td></tr>
<tr><td>diag_study_day</td><td>Study Day of Diagnosis</td><td>Study day of diagnosis derived from the ‘diag_date’ variable. Calculated by subtracting the diag_date from ‘RFSTDTC’ variable in the Demographics table.</td></tr>
</table>

Required entries: NA

Non-Duplicate Key:
The following variables are used to ensure there are no duplicate data:

- USUBJID
- STUDYID
- indication_cat
- SITEID
- indication
- diag_date

Additional Information:
The following table lists indications and their corresponding list of categories. Indications with associated categories are required to have their categories listed.

indication	indication_cat
ALL	NA
AML	NA
CLL	NA
CML	NA
HL	Lymphocyte depleted
HL	Lymphocyte-rich
HL	Mixed cellularity
HL	Nodular lymphocyte predominant Hodgkin lymphoma
HL	Nodular sclerosis
HL	Other
MM	NA
NHL	B Cell (Not Otherwise Specified)
NHL	Burkitt lymphoma
NHL	Diffuse Large B cell
NHL	Follicular
NHL	Mantle Cell Lymphoma
NHL	Marginal zone
NHL	Primary Mediastinal B Cell Lymphoma

Adverse Events

<u>Table Name</u>: ADVERSE_EVENTS

<u>Description</u>: Contains a list of adverse events experienced by patients during the course of the study, or present at the beginning of the study.

<u>SDTM Domain</u>**: Adverse Events (AE), Clinical Events (CE)**

<table>
<tr><th>Variable Name</th><th>Variable Label</th><th>Description</th></tr>
<tr><td>USUBJID</td><td>Unique Subject Identifier</td><td>Unique patient id.</td></tr>
<tr><td>STUDYID</td><td>Study Identifier</td><td>Unique study id.</td></tr>
<tr><td>SITEID</td><td>Site Identifier</td><td>Unique identifier for the site where the study occurs.</td></tr>
<tr><td>AECAT</td><td>Category for Adverse Event</td><td>Category used to group related adverse event. Use the following labels:
<table>
<tr><th>Label</th><th>Description</th></tr>
<tr><td>PRE_TRT</td><td>Adverse event that manifests before treatment administration.</td></tr>
<tr><td>CRS</td><td>Adverse event that is attributable to treatment induced CRS (Cytokine Release Syndrome).</td></tr>
<tr><td>POST_TRT</td><td>Adverse events not including CRS that manifests during and after treatment administration.</td></tr>
<tr><td>LT</td><td>Adverse events found upon long term follow-up.</td></tr>
</table></td></tr>
<tr><td>AESCAT</td><td>Subcategory for Adverse Event</td><td>Further sub-groups within category. Use the following labels:
<table>
<tr><th>Label</th><th>Description</th></tr>
<tr><td>CRS_TOX</td><td>Cumulative toxicity of CRS event.</td></tr>
<tr><td>NEURO_TOX</td><td>Adverse event with CNS involvement.</td></tr>
<tr><td>CARDIAC_TOX</td><td>Adverse event with cardiac involvement.</td></tr>
<tr><td>RENAL_TOX</td><td>Adverse event with renal involvement.</td></tr>
<tr><td>PULMONARY_TOX</td><td>Adverse event with pulmonary involvement.</td></tr>
<tr><td>OTHER_TOX</td><td></td></tr>
</table></td></tr>
<tr><td>AESCAT_MAP</td><td>Mapped Subcategory for Adverse Event</td><td>Sub-group determined using the custom adverse event mapping function. The user selected adverse event map will determine the potential values for this variable.</td></tr>
<tr><td>AETERM</td><td>Reported Term for Adverse Event</td><td>Name of adverse event.</td></tr>
<tr><td>AEDECOD</td><td>Dictionary-Derived Term</td><td>Standardized text description of Adverse Event term using a standard dictionary such as MedDRA.</td></tr>
<tr><td>AEDECOD_MAP</td><td>Mapped Dictionary-Derived Term</td><td>Dictionary derived term derived using the adverse event reference table. The adverse event reference table was created using MedDRA 20.1. Users can map at the preferred term, lower level term, and system organ class level.</td></tr>
<tr><td>AEDICT</td><td>Adverse Event Term Dictionary</td><td>Standard used for AEDECOD (e.g. MedDRA).</td></tr>
<tr><td>AEVER</td><td>Version of Adverse Event Term Dictionary</td><td>Version of standard used for AEDECOD.</td></tr>
</table>

**AETOXGR**	**Standard Toxicity Grade**	Toxicity grade for adverse events based on given criteria.
**GRCRIT**	**Standard used for Toxicity Grade**	Standard used for grading adverse event (e.g. CTCAE).
**GRCRIVER**	**Version of Standard used for Toxicity Grade**	Version of grading standard used.
**AESTDTC**	**Start Date/Time of Adverse Event**	Date and time when adverse event began manifesting. Use ISO 8601 format. All dates listed as YYYY-MM-DD T HH:MM: SS.
**AEENDTC**	**End Date/Time of Adverse Event**	Date and time when adverse event ended due to resolution or censoring. Use ISO 8601 format. All dates listed as YYYY-MM-DD T HH:MM: SS.
**ae_study_day**	**Study Day of the Start of Adverse Event**	Study day of the start of the adverse event derived from the 'AESTDTC' variable. Calculated by subtracting the AESTDTC from 'RFSTDTC' variable in the Demographics table.
**ae_end_study_day**	**Study Day of the End of Adverse Event**	Study day of the end of the adverse event derived from the 'AEENDTC' variable. Calculated by subtracting the AEENDTC from 'RFSTDTC' variable in the Demographics table.

Required entries:

NA

Non-Duplicate Key:

The following variables are used to ensure there are no duplicate data:

- USUBJID
- SITEID
- STUDYID
- AESCAT
- AETERM
- AEDECOD
- AEDICT
- AEVER
- AETOXGR
- GRCRIT
- GRCRIVER
- AESTDTC
- AEENDTC

Additional Information:

NA

Treatment

Table Name: TREATMENT

Description: Contains a list of non-CART treatments administered to patients. Each administration should be in a separate row.

SDTM Domain: **Concomitant/Prior Medications (CM)**

<table>
<tr><th>Variable Name</th><th>Variable Label</th><th>Description</th></tr>
<tr><td>USUBJID</td><td>Unique Subject Identifier</td><td>Unique patient id.</td></tr>
<tr><td>STUDYID</td><td>Study Identifier</td><td>Unique study id.</td></tr>
<tr><td>SITEID</td><td>Site Identifier</td><td>Unique identifier for the site where the study occurs.</td></tr>
<tr><td>CMCAT</td><td>Category of treatment</td><td>Category used to group related adverse event. Use the following labels:
<table>
<tr><th>Label</th><th>Description</th></tr>
<tr><td>PRE_TRT</td><td>Treatments administered before CART administration.</td></tr>
<tr><td>POST_TRT</td><td>Treatments administered after CART administration.</td></tr>
</table></td></tr>
<tr><td>CMSCAT</td><td>Subcategory of treatment</td><td>Further sub-groups within category. Use the following labels:
<table>
<tr><th>Label</th><th>Description</th></tr>
<tr><td>OTHER_CHEMO</td><td>Chemotherapy other than Lymphodepletion.</td></tr>
<tr><td>LD</td><td>Treatments that are part of the pre-CART Lymphodepletion regiment.</td></tr>
<tr><td>CRS_MGMT</td><td>Treatments administered to manage CRS.</td></tr>
<tr><td>CONCOMITANT</td><td>All other non-CART treatments.</td></tr>
</table></td></tr>
<tr><td>CMFLEX_MAP</td><td>Flexible mapping category for Treatment</td><td>Category determined using the custom concomitant medication mapping function. Users can categorize by anatomic therapeutic class (ATC), active ingredient, chemical abstracts service (CAS) number, and unique ingredient identifier (UNII) number. The user selected adverse event map will determine the potential values for this variable.</td></tr>
<tr><td>CMTRT</td><td>Reported Name of Treatment</td><td>Name of treatment. Use generic name when possible.</td></tr>
<tr><td>CMDECOD</td><td>Standardized Name of Treatment</td><td>Standardized identification for treatment (If available).</td></tr>
<tr><td>CMDECOD_MAP</td><td>Mapped Dictionary-Derived Treatment</td><td>Dictionary derived term derived using the concomitant medication reference table. The adverse event reference table was created using the World Health Organization (WHO) B3 Drug Dictionary from March 2020.</td></tr>
<tr><td>CMDECOD_MAPID</td><td>Mapped Dictionary-Derived Treatment ID</td><td>Reference ID for the reference table derived term in CMDECOD_MAP.</td></tr>
<tr><td>CMDICT</td><td>Dictionary used for Standardized Treatment Name</td><td>Criteria used for CMDECOD (e.g. NDC).</td></tr>
</table>

**CMVER**	**Version of Dictionary used for Standardized Treatment Name**	Version of criteria used for CMDECOD.
**CMDOSE**	**Dose per Administration**	Dose per administration of treatment.
**CMDOSU**	**Dose Units**	Unit of dose (e.g. mg, mg/ml).
**CMDOSFRQ**	**Dose Frequency**	Dosing Frequency per interval (e.g. bid).
**CMROUTE**	**Route of Administration**	Route of Administration (e.g. oral, I.V.).
**CMSTDTC**	**Start Date/Time of Treatment**	Date and time when treatment administration began. Use ISO 8601 format. All dates listed as YYYY-MM-DD T HH:MM: SS.
**CMENDTC**	**End Date/Time of Treatment**	Date and time when treatment administration ended. Use ISO 8601 format. All dates listed as YYYY-MM-DD T HH:MM: SS.
**cm_study_day**	**Study Day of the Start of Treatment**	Study day of the start of the treatment derived from the 'CMSTDTC' variable. Calculated by subtracting the CMSTDTC from 'RFSTDTC' variable in the Demographics table.
**cm_end_study_day**	**Study Day of the End of Treatment**	Study day of the end of the treatment derived from the 'CMENDTC' variable. Calculated by subtracting the CMENDTC from 'RFSTDTC' variable in the Demographics table.

Required entries:

NA

Non-Duplicate Key:

The following variables are used to ensure there are no duplicate data:

- USUBJID
- CMSCAT
- CMVER
- CMROUTE
- SITEID
- CMTRT
- CMDOSE
- CMSTDTC
- STUDYID
- CMDECOD
- CMDOSU
- CMENDTC
- CMCAT
- CMDICT
- CMDOSFRQ

Additional Information:

NA

CAR T Treatment

Table Name: CART_TRT

Description: Contains a list of CART infusions administered to patients. Each infusion should be in a separate row.

SDTM Domain: **Exposure (EX), Exposure as Collected (EC)**

Variable Name	Variable Label	Description
**USUBJID**	**Unique Subject Identifier**	Unique patient id.
**STUDYID**	**Study Identifier**	Unique study id.
**SITEID**	**Site Identifier**	Unique identifier for the site where the study occurs.
**PRODUCTID**	**Product Identifier**	Unique Product Identifier.
**dose_num**	**Dose Number**	Each dose administered to a patient is sequentially numbered starting from 1.
**infusion_num**	**Infusion Number**	For each dose, each infusion (split) is sequentially numbered starting from 1.
**trans_cell_cnt**	**Transduced Cell Count**	Total transduced cell count.
**nucl_cell_cnt**	**Nucleated Cell Count**	Total nucleated cell count.
**start_date_time**	**Start Date/Time of Treatment**	Date and time when treatment administration began. Use ISO 8601 format. All dates listed as YYYY-MM-DD T HH:MM: SS.
**infusion_study_day**	**Study day of Treatment**	Study day of CART product administration derived from the 'start_date_time' variable. Calculated by subtracting the start_date_time from the 'RFSTDTC' variable in the Demographics table.

Required entries:
All infusions are required to be entered into this table.

Non-Duplicate Key:
The following variables are used to ensure there are no duplicate data:

- USUBJID
- STUDYID
- infusion_num
- SITEID
- dose_num
- start_date_time

Additional Information: NA

Lab Results

Table Name: LAB_RESULTS

Description: Contains information related to cytokine level measurements, persistence of CAR T cells after administration, temperature data, and other laboratory findings.

SDTM Domain: **Laboratory Test Results (LB), Vital Signs (VS), Pharmacokinetic Concentrations (PC)**

<table>
<tr><th>Variable Name</th><th>Variable Label</th><th>Description</th></tr>
<tr><td>USUBJID</td><td>Unique Subject Identifier</td><td>Unique patient id.</td></tr>
<tr><td>STUDYID</td><td>Study Identifier</td><td>Unique study id.</td></tr>
<tr><td>SITEID</td><td>Site Identifier</td><td>Unique identifier for the site where the study occurs.</td></tr>
<tr><td>LABCAT</td><td>Category of Lab Test</td><td>Category for lab test data. Use the following labels:
<table>
<tr><th>Label</th><th>Description</th></tr>
<tr><td>CYTOKINE</td><td>Cytokine measurements from blood</td></tr>
<tr><td>CYTOKINE_CSF</td><td>Cytokine measurements from cerebrospinal fluid</td></tr>
<tr><td>LAB</td><td></td></tr>
<tr><td>PERSISTENCE</td><td>Persistence measurements from blood</td></tr>
<tr><td>PERSISTENCE_BM</td><td>Persistence measurements from bone marrow</td></tr>
<tr><td>TEMP</td><td>Temperature data in degrees Celsius (˚C)</td></tr>
</table></td></tr>
<tr><td>LBTEST</td><td>Lab Test</td><td>Name of laboratory test being measured.</td></tr>
<tr><td>LBTEST_MAPPED</td><td>Mapped Lab Test</td><td>Dictionary derived term derived using the cytokine reference table.</td></tr>
<tr><td>LBORRES</td><td>Amount of Cytokine</td><td>Result of the laboratory test</td></tr>
<tr><td>LBORRESU</td><td>Unit for Amount of Cytokine</td><td>Unit for the result of the laboratory test.</td></tr>
<tr><td>LBDTC</td><td>Date/time of Sample Collection</td><td>Date/time of sample collection. Use ISO 8601 format. All dates listed as YYYY-MM-DD T HH:MM: SS.</td></tr>
<tr><td>lab_study_day</td><td>Study Day of Sample Collection</td><td>Study day of lab test derived from the ‘LBDTC’ variable. Calculated by subtracting the LBDTC from the ‘RFSTDTC’ variable in the Demographics table</td></tr>
<tr><td>lab_type</td><td>Lab type</td><td>Name of laboratory where test was conducted.</td></tr>
</table>

Required entries:

All measured cytokines, persistence of CAR T cells, and temperature data are required to be entered into this table. Other laboratory information may or may not be included.

Non-Duplicate Key:
The following variables are used to ensure there are no duplicate data:

- USUBJID
- STUDYID
- LBCAT
- LBTEST
- LBORRES
- LBORRESU
- LBDTC
- lab_type

Additional Information: NA

Disease Status

Table Name: DISEASE_STATUS

Description: Contains information about the status of disease at different points of assessment. If available, disease related pre-study assessments should be placed in this table.

SDTM Domain: **Disease Response (RS)**

Variable Name	Variable Label	Description
**USUBJID**	**Unique Subject Identifier**	Unique patient id.
**STUDYID**	**Study Identifier**	Unique study id.
**SITEID**	**Site Identifier**	Unique identifier for the site where the study occurs.
**RSTESTCD**	**Response Assessment Short Name**	Short name of disease assessment test. See below for required assessments.
**RSTEST**	**Response Assessment Name**	Verbatim name of the response assessment.
**RSTEST_MAPPED**	**Mapped Response Assessment Name**	Dictionary derived term derived using the disease response reference table.
**location**	**Anatomical Location of Assessment**	Anatomical location of assessment. See below for required locations.
**criteria**	**Criteria used for Response Assessment Result**	Criteria used for assessment. See below for required criteria.
**RSORRES**	**Response Assessment Result**	Result of assessment.
**RSDTC**	**Date/Time of Response Assessment**	Date time when assessment was made. Use ISO 8601 format. All dates listed as YYYY-MM-DD T HH:MM: SS.
**rs_study_day**	**Study Day of Response Assessment**	Study day of response assessment derived from the ‘RSDTC’ variable. Calculated by subtracting the RSDTC from the ‘RFSTDTC’ variable in the Demographics table.

Required entries: NA

Non-Duplicate Key:
The following variables are used to ensure there are no duplicate data:

- USUBJID
- STUDYID
- RSTESTCD
- RSTEST
- RSORRES
- location
- RSDTC
- criteria

Additional Information: NA

Medical History

Table Name: MEDICAL_HISTORY

Description: Contains patient's medical history information.

SDTM Domain: **Medical History (MH)**

Variable Name	Variable Label	Description
**USUBJID**	**Unique Subject Identifier**	Unique patient id.
**STUDYID**	**Study Identifier**	Unique study id.
**SITEID**	**Site Identifier**	Unique identifier for the site where the study occurs.
**MHTERM**	**Reported Term**	Term for describing medical condition.
**MHDECOD**	**Dictionary-Derived Term**	Dictionary derived text description of term. Equivalent to PT in MedDRA. Dictionary name and version should be provided in **term_std** and **term_std_ver**.
**term_std**	**Dictionary used for Dictionary-Derived Term**	Dictionary used for Medical History term (e.g. MedDRA).
**term_std _ver**	**Version of Dictionary used for Dictionary-Derived Term**	Version of Dictionary used for Medical History term.
**MHTOXGR**	**Medical History Toxicity Grade**	Standard Toxicity Grade for Medical History term.
**GRCRIT**	**Toxicity Grading Criteria**	Grading criteria used for Medical History term toxicity grade.
**GRCRIVER**	**Toxicity Grading Criteria Version**	Version of grading criteria used for Medical History term toxicity grade.
**MHSTDTC**	**Start Date/Time of Medical History Event**	Date of diagnosis / date of event. Use ISO 8601 format. All dates listed as YYYY-MM-DD T HH:MM: SS.
**MHENDTC**	**End Date/Time of Medical History Event**	Date of resolution / end date of event. Use ISO 8601 format. All dates listed as YYYY-MM-DD T HH:MM: SS.
**mh_study_day**	**Study day of Start of Medical History Event**	Study day of medical history start date derived from the 'MHSTDTC' variable. Calculated by subtracting the MHSTDTC from the 'RFSTDTC' variable in the Demographics table.
**mh_end_study_day**	**Study Day of Medical History Event**	Study day of medical history end date derived from the 'MHENDTC' variable. Calculated by subtracting the MHENDTC from the 'RFSTDTC' variable in the Demographics table.
**MHONGO**	**Medical History Ongoing**	Binary variable to determine if medical history event is ongoing at start of the study period.

Required entries:

All disease related pre-study medical history is required. This includes date of onset and anatomical location of each relapse. For example, we are interested in knowing if the patient had a prior history of CNS relapse pre-study, whether in remission or not.

Non-Duplicate Key:
The following variables are used to ensure there are no duplicate data:

- USUBJID
- STUDYID
- MHTERM
- MHDECOD
- term_std
- term_std_ver
- GRCRIT
- GRCRIVER
- MHSTDTC
- MHONGO

Additional Information: NA

Product

Table Name: PRODUCT

Description: Contains detailed product information.

SDTM Domain: Custom

Variable Name	Variable Label	Description
**USUBJID**	**Unique Subject Identifier**	Unique patient id.
**STUDYID**	**Study Identifier**	Unique study id.
**SITEID**	**Site Identifier**	Unique identifier for the site where the study occurs.
**product_id**	**Product Id**	Unique identifier for the product.
**product_name**	**Product Name**	Name of the product.
**PRODCAT**	**Product Category**	Type of Product (e.g. CD4, CD8, Mixed)
**donor_type**	**Donor Type**	Source of CART cells (e.g. Use Autologous or Allogeneic)
**VECID**	**Vector Identifier**	Unique Product Vector Identifier
**VECLOTID**	**Vector Lot Identifier**	Unique Product Vector Lot Identifier
**MANUFDT**	**Manufacturing Date**	Date Product Manufacturing was completed. Use ISO 8601 format. All dates listed as YYYY-MM-DD T HH:MM: SS.
**prod_manuf_study_day**	**Study Day of Manufacturing Date**	Study day of manufacturing date derived from the 'MANUFDT' variable. Calculated by subtracting the MANUFDT from the 'RFSTDTC' variable in the Demographics table.

Required entries: NA.

Non-Duplicate Key:
The following variables are used to ensure there are no duplicate data:

- PRODUCTID

Additional Information: NA

Cell Product

Table Name: CELL_PRODUCT

Description: Descriptions or measurements of CAR T cell product attributes typically contained in the product Certificate of Analysis. Example attributes include appearance, measures of potency, vector copy number (VCN), and off target rate (for genome edited products).

SDTM Domain: Custom

Variable Name	Variable Label	Description
**PRODUCTID**	**Unique Product Identifier**	Unique Product Identifier.
**CLPRDCAT**	**Category of Cell Product attribute**	Category used to group related cell product attributes.
**CLPRDTEST**	**Name of cell product attribute**	Name of cell product attribute.
**CLPRDORRES**	**Result of cell product attribute assessment**	Result of cell product attribute assessment.
**CLPRDORRESU**	**Unit of assessment**	Unit of assessment.
**CLPRDDTC**	**Date/Time of Assessment**	Date/Time of Assessment. Use ISO 8601 format. All dates listed as YYYY-MM-DD T HH:MM: SS.
**clprd_study_day**	**Study Day of Date of Assessment**	Study day of date of assessment derived from the 'CLPRDDTC' variable. Calculated by subtracting the CLPRDDTC from the 'RFSTDTC' variable in the Demographics table.

Required entries: NA

Non-Duplicate Key:
The following variables are used to ensure there are no duplicate data:

- PRODUCTID
- CLPRDTEST

Additional Information: NA

Cell Components

Table Name: CELL_COMPONENTS

Description: Measurements of cellular constituents during product manufacturing. Cell measurements at any point during manufacturing (including during cell expansion and in the final product) should be included in this table. Cell types and associated surface markers should be indicated in the CELL and MARKER columns. The type of measurements, for example cell count or percentage of cells, should be indicated in the CLCPTEST column. If the measurement was performed on a subset of cell types, this parent cell population should be identified using the DENOM_CELL and DENOM_MARKER columns. For cell expansion, measurements of different cell types, including CAR+ cells, from any in-process time point should be included in this table. Measurements at the start of expansion, as well as at harvest time, should also be included. Mean and median fluorescence for the CAR+ population should also be included in this table.

SDTM Domain: Custom.

Variable Name	Variable Label	Description
**PRODUCTID**	**Unique Product Identifier**	Unique Product Identifier.
**CLCMPCAT**	**Stage of manufacturing**	Stage of manufacturing when measurements of cellular constituents were made (e.g. Release, Apheresis, Cryopreserved).
**CELL**	**Cell Type**	Cell type being measured.
**CELLID**	**Cell Type Identifier**	Identification number from the cell type standard table for the cell type being measured.
**STD**	**Standard**	Standard dictionary from the cell type standard table for the cell type being measured.
**MARKER**	**Cell Marker**	Cell surface markers used for staining.
**MARKERID**	**Cell Marker Identifier**	Identification number from the cell marker standard table for the cell marker being measured.
**CLCMPTEST**	**Test type**	The type of measurement performed (count, percentage, volume, mean/median fluorescent intensity etc.).
**DENOM_CELL**	**Denominator cell type**	Parent cell population from which measurement was taken (if any).
**DENOM_CELLID**	**Denominator cell type Identifier**	Identification number from the cell type standard table for the parent cell population from which measurement was taken (if any).
**DENOM_MARKER**	**Denominator markers**	Cell surface markers of parent cell population (if any).
**DENOM_MARKERID**	**Denominator markers Identifier**	Identification number from the cell marker standard table for the cell surface markers of parent cell population (if any).
**CLCMPORRES**	**Result of cell component measurement**	Result of cell component measurement.
**CLCMPORRESU**	**Unit of cell component measurement**	Unit of cell component measurement.
**METHOD**	**Method of measurement**	Method used for cell component measurement.
**CLCMPSTDTC**	**Date/Time of measurement**	Date/Time of measurement. Use ISO 8601 format. All dates listed as YYYY-MM-DD T HH:MM: SS.
**clcmp_study_day**	**Study Day of Date/Time of measurement**	Study day of date of measurement derived from the ‘CLCMPSTDTC’ variable. Calculated by subtracting the

		CLCMPSTDTC from the 'RFSTDTC' variable in the Demographics table.

Required entries: NA

Non-Duplicate Key:
The following variables are used to ensure there are no duplicate data:

- PRODUCTID
- CLCMPCAT
- CELLID
- STD
- MARKERID
- CLCMPTEST
- DENOM_CELLID
- DENOM_MARKERID
- CLCMPORRESU
- METHOD
- CLCMPSTDTC

Additional Information: NA

Vector

Table Name: VECTOR

Description: Table describing the design of the vector encoding the CAR transgene. Example attributes include vector type (lentivirus, plasmid, etc.), backbone, costimulatory domain, and svFc clone.

SDTM Domain: Custom.

Variable Name	Variable Label	Description
**VECID**	**Unique vector identifier**	Unique vector identifier.
**VECNAME**	**Name of Vector**	Name of the vector
**VECTEST**	**Name of vector attribute**	Name of vector attribute being described or measured (e.g., vector type, backbone, costimulatory domain, antigen species).
**VECORRES**	**Result of vector assessment**	Result of vector assessment.
**VECORRESU**	**Unit of assessment**	Unit of assessment if relevant.
**VECSTDTC**	**Date/Time of Assessment**	Date/Time of Assessment. Use ISO 8601 format. All dates listed as YYYY-MM-DD T HH:MM: SS.

Required entries: NA

Non-Duplicate Key:
The following variables are used to ensure there are no duplicate data:

- VECNAME
- VECTEST

Additional Information: NA

Vector Lot

Table Name: VECTORLOT

Description: Table describing lot release information for the CAR transgene vector. Attributes of the vector lot which are typically contained in the Certificate of Analysis should be included in this table. Example attributes include transducing units (TU), and residual host cell DNA+.

SDTM Domain: Custom.

Variable Name	Variable Label	Description
**VECLOTID**	**Unique vector lot identifier**	Unique vector lot identifier.
**VECLOTNAME**	**Vector Lot Name**	Name of the vector lot.
**VECLOTTEST**	**Name of vector lot attribute**	Name of vector lot attribute being described or measured.
**VECLOTORRES**	**Result of vector lot assessment**	Result of vector lot assessment.
**VECLOTORRESU**	**Unit of assessment**	Unit of assessment.
**VECLOTSTDTC**	**Date/Time of Assessment**	Date/Time of Assessment. Use ISO 8601 format. All dates listed as YYYY-MM-DD T HH:MM: SS.

Required entries: NA

Non-Duplicate Key:
The following variables are used to ensure there are no duplicate data:
- VECLOTNAME
- VECLOTTEST

Additional Information: NA

Genome Editing Tool

Table Name: GENOME_EDITING_TOOL

Description: Table describing the design of the agent used to edit the T cell genome. This table should be filled if genome editing was performed on the product other than the transfer of the CAR transgene. The table should include the attributes of the genome editing tools including the genome target, the components of the genome editing tool, the mechanism by which the tools are introduced into the cells (e.g., electroporation, AAV vector) and the DNA repair pathway (e.g. NHEJ, HDR) being utilized for the given target.

SDTM Domain: Custom.

<table>
<tr><th>Variable Name</th><th>Variable Label</th><th>Description</th></tr>
<tr><td>GEID</td><td>Unique identifier for gene editing agent</td><td>Unique identifier for gene editing agent.</td></tr>
<tr><td>GECAT</td><td>Category of gene editing agent</td><td>Category of gene editing agent:<table><tr><th>Category</th></tr><tr><td>TALEN</td></tr><tr><td>ZFN</td></tr><tr><td>CRISPR-CAS</td></tr></table></td></tr>
<tr><td>GETEST</td><td>Name of measured/assessed attribute</td><td>Name of measured/assessed attribute.</td></tr>
<tr><td>PRODUCTID</td><td>Unique Product Identifier</td><td>Unique Product Identifier.</td></tr>
<tr><td>GEORRES</td><td>Result of assessment</td><td>Result of assessment</td></tr>
<tr><td>GEORRESU</td><td>Unit of assessment</td><td>Unit of assessment.</td></tr>
</table>

Required entries: NA

Non-Duplicate Key: NA

Additional Information: NA

Supplemental References

1. Stein, A.M., S.A. Grupp, J.E. Levine, T.W. Laetsch, M.A. Pulsipher, M.W. Boyer, K.J. August, B.L. Levine, L. Tomassian, S. Shah, et al., *Tisagenlecleucel Model-Based Cellular Kinetic Analysis of Chimeric Antigen Receptor-T Cells.* CPT Pharmacometrics Syst Pharmacol, 2019. **8**(5): p. 285-295.
2. Shukla, S.N. and B.M. Marlin, *Modeling Irregularly Sampled Clinical Time Series.* arXiv preprint arXiv:1812.00531, 2018.
3. Beal, S., L. Sheiner, A. Boeckmann, and R. Bauer, *NONMEM 7.4 users guides.* ICON plc, Gaithersburg, MD, 1989. **2018**.
4. Honaker, J., G. King, and M. Blackwell, *Amelia II: A program for missing data.* Journal of statistical software, 2011. **45**(7): p. 1-47.
5. Pedregosa, F., G. Varoquaux, A. Gramfort, V. Michel, B. Thirion, O. Grisel, M. Blondel, P. Prettenhofer, R. Weiss, and V. Dubourg, *Scikit-learn: Machine learning in Python.* the Journal of machine Learning research, 2011. **12**: p. 2825-2830.
