# Supplementary material for: Cross-study safety analysis of risk factors in CAR T cell clinical trials: An FDA database pilot project
Source: Mol Ther Oncolytics. 2022 Oct 20;27:182–94. doi: 10.1016/j.omto.2022.10.006 (PMC9643340; doi:10.1016/j.omto.2022.10.006)
Supplement: Document S2. Article plus supplemental information [file mmc2.pdf]

Molecular Therapy
Oncolytics
Original Article

Cross-study safety analysis of risk factors in CAR T cell clinical trials: An FDA database pilot project

Matthew Foster,[1] Yonatan Negash,[1] Leslie Eberhardt,[1] Wilson W. Bryan,[2] Kimberly Schultz,[2] Xiaofei Wang,[2] Yuan Xu,[3] and Bindu George[2]

[1]Science Applications International Corporation (SAIC), Reston, VA 20190, USA; [2]Office of Tissues and Advanced Therapies (OTAT), Center for Biologics Evaluation and Research (CBER), U.S. Food and Drug Administration (FDA), Silver Spring, MD 20993, USA; [3]Office of Translational Sciences (OTS), Center for Drug Evaluation and Research (CDER), U.S. Food and Drug Administration (FDA), Silver Spring, MD 20993, USA

The Chimeric Antigen Receptor (CAR) T Cell Safety Database Project explored the use of cross-study safety data to identify risk factors associated with severe cytokine release syndrome (sCRS) and severe neurological toxicities (sNTX) after CAR T cell administration. Sponsors voluntarily submitted data for 1,926 subjects from 17 phases 1 and 2 studies (six acute lymphocytic leukemia [ALL], five non-Hodgkin's lymphoma [NHL], and six multiple myeloma [MM] studies). Subjects with ALL had a higher risk for developing sCRS and sNTX compared with subjects with NHL or MM. Subjects who received CAR T cells produced with gammaretrovirus vectors including CD28 sequences had higher rates of sNTX compared with subjects who received products produced with other vector designs included in the database. Use of cytokine-directed therapies and corticosteroids at lower toxicity grades were associated with lower rates of sCRS. Although this exploratory study was limited by unadjusted cross-study comparisons, it independently reproduced known risk factors for CAR T cell toxicity. Findings provide stakeholders in the CAR T cell clinical development community information on safety trends for consideration in early phase clinical trial design, as well as avenues for additional research.

INTRODUCTION

Despite the approval of six chimeric antigen receptor (CAR) T cell products, cytokine release syndrome (CRS), a cytokine-mediated systemic inflammatory response, and neurological toxicities (NTX) continue to be life-threatening adverse events associated with CAR T cell administration.[1–7] The clinical presentation of CRS varies from mild flu-like symptoms to high fevers, sinus tachycardia, hypotension, hypoxia, depressed cardiac function, and other signs of organ dysfunction, while NTX presents as a cluster of neurological symptoms ranging from mild confusion, headaches, and hallucinations to aphasia, seizures, and somnolence.[8] CRS pathophysiology is postulated to be related to CAR T cell activation, expansion, and recruitment of other immune mediator cells, resulting in cascading cytokine production. Although the pathophysiology of NTX remains unknown, the leading hypothesis is a cytokine-mediated disruption of the blood-brain barrier.[9] Symptoms of CRS and NTX often occur concurrently, and groups such as the American Society for Transplantation and Cellular Therapy (ASTCT) have published guidelines to better harmonize the definitions and grading systems for these adverse events.[10] Treatment of CRS consists primarily of symptomatic treatment at lower toxicity grades but may involve the use of supplemental oxygen, high-dose vasopressor therapy, and tocilizumab (a humanized anti-human IL-6 receptor monoclonal antibody) with or without concomitant corticosteroid treatment for worsening CRS.[7,11] Treatment strategies for NTX vary among different centers but consist primarily of corticosteroid therapy with or without cytokine-directed therapies and supportive care.

Many groups have attempted to identify risk factors associated with the development of CRS and NTX after CAR T cell product administration. Teachey et al.[12] measured cytokines and clinical biomarkers in 51 subjects and found that peak levels of 24 cytokines (including IFN-γ, IL-6, sgp130, and sIL-6R) in the first month after infusion were highly associated with severe CRS. In addition, they developed multiple predictive models, including a logistic regression model, which used a combination of IFN-γ and CCL3 to predict which subjects would develop severe CRS (sensitivity 82%, specificity 93%). Hay et al.[13] performed multivariate analysis of baseline characteristics in 133 adult subjects who received CD19 CAR T cells and identified high marrow tumor burden, lymphodepletion using fludarabine and cyclophosphamide, higher CAR T cell dose, thrombocytopenia before lymphodepletion, and manufacturing of CAR T cells without selection of $CD8^+$ central memory T cells as independent predictors of CRS. Brudno and Kochenderfer[14] performed a review of factors contributing to CRS and NTX and found that higher peak *in vivo* proliferation of

Received 24 February 2022; accepted 18 October 2022;
https://doi.org/10.1016/j.omto.2022.10.006.

Correspondence: Bindu George, Office of Tissues and Advanced Therapies (OTAT), Center for Biologics Evaluation and Research (CBER), U.S. Food and Drug Administration (FDA), Silver Spring, MD 20993, USA
E-mail: bindu.george@fda.hhs.gov

This is an open access article under the CC BY license (http://creativecommons.org/licenses/by/4.0/).

CAR T cells, higher cell doses, conditioning chemotherapy containing fludarabine, acute lymphocytic leukemia (ALL) rather than non-Hodgkin's lymphoma (NHL), higher burden of disease, baseline thrombocytopenia, and baseline elevated markers of endothelial activation (e.g., angiopoietin-2 and von Willebrand factor) were all risk factors for both CRS and NTX. In addition, they found that the CAR structure may contribute to patterns of toxicity. Tedesco and Mohan[15] performed a systematic review of 33 CAR T cell clinical trials to identify biomarkers predictive of post-treatment CRS and NTX and found that circulating IL-6, IFN-ɣ, IL-10, and IL-15 appear to be associated with the severity of CAR T cell therapy toxicities in both leukemia and lymphoma subjects. Greenbaum et al.[16] found that endothelial activation and stress index, a clinical surrogate for endothelial dysfunction, combined with ferritin and C-reactive protein was associated with both the incidence and severity of CRS and immune effector cell-associated neurotoxicity syndrome (ICANS) in subjects treated with CAR T cell products.

Although academic researchers have made important contributions to the current understanding of risk factors associated with CAR T cell-induced CRS and NTX, many groups are limited by either relatively small sample sizes or limited access to patient-level data. In order to address these limitations and increase the statistical power to detect risk factors associated with CAR T cell product administration, the FDA Center for Biologics Evaluation and Research (CBER), Office of Tissues and Advanced Therapies (OTAT), initiated a project to (1) assess the feasibility of integrating cross-study CAR T cell product safety data into a central CAR T cell safety database and (2) perform exploratory analyses to validate the use of cross-study data for risk factor identification and predictive modeling.[17] In this project, clinical safety and chemistry manufacturing and control (CMC) data from 17 studies were voluntarily provided by sponsors and incorporated into a CAR T cell safety database. Maximum CRS and NTX grades within 28 days of CAR T cell product administration were calculated for each subject, and rates of severe (toxicity grade ≥ 3) CRS and NTX were compared among various demographic, clinical, and manufacturing groups to identify risk factors associated with severe CRS and NTX after CAR T cell product administration.

RESULTS

The CAR T cell safety database contains data for 1,926 subjects from 17 studies received prior to September 1, 2020. Six studies were phase 1, five were phase 2, and six were phases 1 and 2. Six studies contained primarily ALL subjects, five studies contained NHL subjects, and six studies contained multiple myeloma (MM) subjects. Four studies contained primarily pediatric subjects, while 13 studies contained primarily adult subjects.

A total of 1,277 subjects received at least one administration of a CAR T cell product, while the remaining 649 subjects did not receive treatment because of failure to meet the sponsor's study requirements. Of the 1,277 subjects, 963 (75.4%) received CD19-targeting products, while 314 (24.6%) received B cell maturation antigen (BCMA)-targeting products. Median treated patient count per study was 61 (interquartile range [IQR]: 29–93) across all studies, 49 (IQR: 29–70) for ALL studies, 92 (IQR: 19–182) for NHL studies, and 47 (IQR: 18–79) for MM studies.

A summary table of group sizes, rates of severe CRS and NTX, and odds ratios comparing select variables from pooled analysis are shown in Figure 1. Further results for indication, CRS management protocol, vector design, age group, cytokines, expansion *in vivo*, dosing parameters, and CMC characteristics are reported below.

CRS

Within the first 28 days after CAR T cell administration, 807 subjects (63.2%) experienced CRS and 174 subjects (13.6%) experienced severe CRS (sCRS) (toxicity grade ≥ 3). Median onset time for the first sCRS was 3.9 days post-infusion.

Indication

Of 1,277 treated subjects, 304 (23.8%) had ALL, 659 (51.6%) had NHL, and 314 (24.6%) had multiple myeloma. Subtypes of NHL were not analyzed. ALL subjects had higher rates of sCRS than NHL subjects, while there was no statistically significant difference between NHL and MM subjects in the frequency of sCRS (Figure 1, "Indication"). ALL subjects had higher rates of sCRS than NHL subjects in all subgroups except in subjects with no prior transplantation (Figure 2, "Prior Transplant: No"). An inadequate pediatric NHL sample size (n = 4) did not allow comparisons among pediatrics subjects (Figure 2, "Age Group: Pediatric: NHL"). For products administered to both adult ALL and adult NHL subjects, ALL subjects had higher rates of sCRS (Figure S1A). There was no statistically significant difference in sCRS rates between NHL and MM subjects (Figure 2), except in subjects with no prior transplantation (Figure 2, "Prior Transplant: No").

CRS management protocol

CRS management protocols could be divided into two groups on the basis of the timing of intervention with tocilizumab. In the high-grade intervention (HGI) group, intervention with tocilizumab was generally reserved for sCRS while in the low-grade intervention (LGI) group, tocilizumab intervention could also be used as needed for grade 1 or 2 CRS. Only the rate of sCRS (using maximum CRS grade per subject) was considered in our analysis, not progression of CRS from lower to higher grades. HGI subjects experienced greater rates of sCRS than LGI subjects (Figure 1, "CRS Intervention"). Subgroup analyses supported this finding in all groups except pediatrics (Figure 3, "Age: Pediatric"). Because almost all pediatric subjects had ALL (n = 177 of 181), results from this subgroup analysis may not be applicable in other indications.

Vector design

Four different combinations of vector type (gammaretrovirus or lentivirus) and costimulatory domain (4-1BB or CD28) were used across 17 studies to manufacture CAR T cell products (Table S1). Although subjects receiving CAR T cells with gammaretrovirus

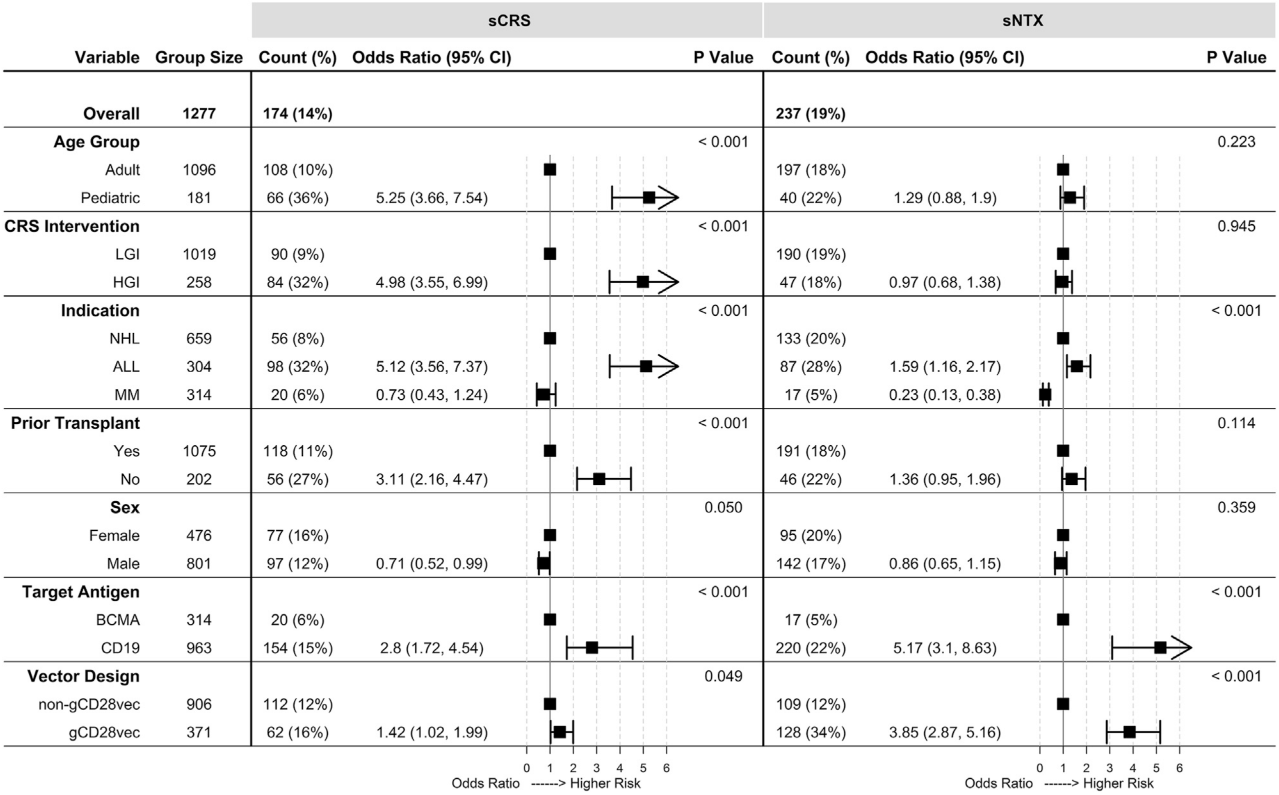

Variable	Group Size	sCRS Count (%)	sCRS Odds Ratio (95% CI)	sCRS P Value	sNTX Count (%)	sNTX Odds Ratio (95% CI)	sNTX P Value
**Overall**	**1277**	**174 (14%)**			**237 (19%)**		
**Age Group**				< 0.001			0.223
Adult	1096	108 (10%)			197 (18%)		
Pediatric	181	66 (36%)	5.25 (3.66, 7.54)		40 (22%)	1.29 (0.88, 1.9)	
**CRS Intervention**				< 0.001			0.945
LGI	1019	90 (9%)			190 (19%)		
HGI	258	84 (32%)	4.98 (3.55, 6.99)		47 (18%)	0.97 (0.68, 1.38)	
**Indication**				< 0.001			< 0.001
NHL	659	56 (8%)			133 (20%)		
ALL	304	98 (32%)	5.12 (3.56, 7.37)		87 (28%)	1.59 (1.16, 2.17)	
MM	314	20 (6%)	0.73 (0.43, 1.24)		17 (5%)	0.23 (0.13, 0.38)	
**Prior Transplant**				< 0.001			0.114
Yes	1075	118 (11%)			191 (18%)		
No	202	56 (27%)	3.11 (2.16, 4.47)		46 (22%)	1.36 (0.95, 1.96)	
**Sex**				0.050			0.359
Female	476	77 (16%)			95 (20%)		
Male	801	97 (12%)	0.71 (0.52, 0.99)		142 (17%)	0.86 (0.65, 1.15)	
**Target Antigen**				< 0.001			< 0.001
BCMA	314	20 (6%)			17 (5%)		
CD19	963	154 (15%)	2.8 (1.72, 4.54)		220 (22%)	5.17 (3.1, 8.63)	
**Vector Design**				0.049			< 0.001
non-gCD28vec	906	112 (12%)			109 (12%)		
gCD28vec	371	62 (16%)	1.42 (1.02, 1.99)		128 (34%)	3.85 (2.87, 5.16)	

Figure 1. Pooled analysis for select clinical and manufacturing variables

The first column lists select variables, while the second column lists the group size. For both severe cytokine release syndrome (sCRS) and severe neurological toxicities (sNTX), the "Count (%)" column gives the number of subjects who experienced sCRS/sNTX as well as the percentage relative to the group size. The "Odds Ratio (95% CI)" columns contain odds ratios and 95% confidence intervals calculated using the first variable in each group as a reference point (reference points: adult for age group, LGI for CRS intervention, NHL for indication, yes for prior transplantation, female for sex, BCMA for target antigen, and non-gCD28vec for vector design). Forest plots are displayed next to the "Odds Ratio (95% CI)" column to visually represent relative risk. The "P Value" column lists p values from chi-square tests comparing severe toxicity rates in each group. ALL, acute lymphocytic leukemia; NHL, non-Hodgkin's lymphoma; MM, multiple myeloma; BCMA, B cell maturation antigen; gCD28vec, products produced with gammaretrovirus vectors with CD28 sequences in the transgene; LGI, low-grade intervention; HGI, high-grade intervention.

vectors containing CD28 sequences (gCD28vec) had higher rates of sCRS (Figure 1, "Vector Design"), this result was inconclusive upon subgroup analysis (Figure 4).

Age group

Although pediatric subjects experienced higher rates of sCRS compared with adults (Figure 1, "Age Group"), this difference did not persist upon indication and CRS management protocol subgroup analyses (Figure S2A). As there were few pediatric subjects with NHL or MM (n = 4 of 181), subgroup analysis of age by indication could not be performed for these indications. Among ALL subjects, there was no statistically significant difference in sCRS rates between adults (26.0% [n = 33 of 127]) and pediatrics (36.7% [n = 65 of 177]) ($p = 0.06$). Further subgroup analysis of ALL subjects by CRS management protocol found no statistically significant difference in sCRS rates between pediatrics and adults in either the LGI (28.6% [n = 10 of 35] versus 23.5% [n = 27 of 115], $p = 0.70$) or the HGI (38.7% [n = 55 of 142] versus 50.0% [n = 6 of 12], $p = 0.54$) groups.

Cytokines

Of 116 cytokines/biomarkers in the CAR T cell safety database, there were data for 17 (IFN-γ, IL-1β, IL-2, IL-4, IL-5, IL-6, IL-7, IL-8, IL-10, IL-12, IL-13, IL-15, CCL2, CCL3, CCL4, GMCSF, and TNF-α) of these for at least 60% of treated subjects. Differences in cytokine levels between subjects who experienced sCRS and those who did not are reported in Tables 1 and S2 and Figure S3.

***Expansion* in vivo**

Higher peak CAR T cell expansion has been shown to be associated with CRS.[18,19] Expansion *in vivo* was evaluated as the peak level (Cmax) of CAR T cell transgene copies in blood within 28 days after CAR T cell product administration. Data were available for 8 of 17 studies and 661 of 1,277 treated subjects. Median maximum transgene concentration was greater in subjects who experienced sCRS (57,988 versus 19,260 transgene copies/μg DNA, $p < 0.001$). Median maximum transgene concentration was greatest for ALL subjects (50,679 transgene copies/μg DNA, n = 243), followed by NHL (15,870 transgene copies/μg DNA, n = 410) and MM (243 transgene

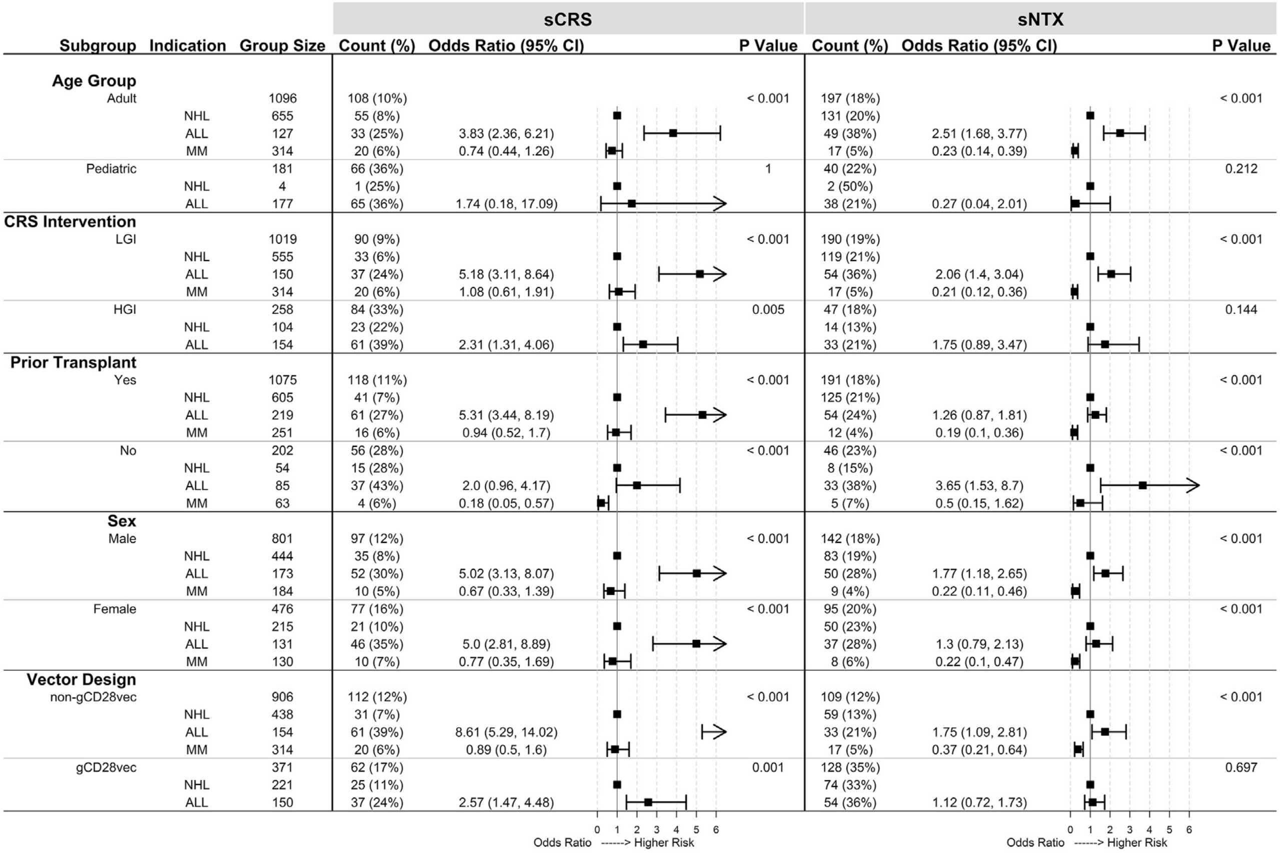

Subgroup	Indication	Group Size	sCRS Count (%)	sCRS Odds Ratio (95% CI)	sCRS P Value	sNTX Count (%)	sNTX Odds Ratio (95% CI)	sNTX P Value
**Age Group**								
Adult		1096	108 (10%)		< 0.001	197 (18%)		< 0.001
	NHL	655	55 (8%)			131 (20%)		
	ALL	127	33 (25%)	3.83 (2.36, 6.21)		49 (38%)	2.51 (1.68, 3.77)	
	MM	314	20 (6%)	0.74 (0.44, 1.26)		17 (5%)	0.23 (0.14, 0.39)	
Pediatric		181	66 (36%)		1	40 (22%)		0.212
	NHL	4	1 (25%)			2 (50%)		
	ALL	177	65 (36%)	1.74 (0.18, 17.09)		38 (21%)	0.27 (0.04, 2.01)	
**CRS Intervention**								
LGI		1019	90 (9%)		< 0.001	190 (19%)		< 0.001
	NHL	555	33 (6%)			119 (21%)		
	ALL	150	37 (24%)	5.18 (3.11, 8.64)		54 (36%)	2.06 (1.4, 3.04)	
	MM	314	20 (6%)	1.08 (0.61, 1.91)		17 (5%)	0.21 (0.12, 0.36)	
HGI		258	84 (33%)		0.005	47 (18%)		0.144
	NHL	104	23 (22%)			14 (13%)		
	ALL	154	61 (39%)	2.31 (1.31, 4.06)		33 (21%)	1.75 (0.89, 3.47)	
**Prior Transplant**								
Yes		1075	118 (11%)		< 0.001	191 (18%)		< 0.001
	NHL	605	41 (7%)			125 (21%)		
	ALL	219	61 (27%)	5.31 (3.44, 8.19)		54 (24%)	1.26 (0.87, 1.81)	
	MM	251	16 (6%)	0.94 (0.52, 1.7)		12 (4%)	0.19 (0.1, 0.36)	
No		202	56 (28%)		< 0.001	46 (23%)		< 0.001
	NHL	54	15 (28%)			8 (15%)		
	ALL	85	37 (43%)	2.0 (0.96, 4.17)		33 (38%)	3.65 (1.53, 8.7)	
	MM	63	4 (6%)	0.18 (0.05, 0.57)		5 (7%)	0.5 (0.15, 1.62)	
**Sex**								
Male		801	97 (12%)		< 0.001	142 (18%)		< 0.001
	NHL	444	35 (8%)			83 (19%)		
	ALL	173	52 (30%)	5.02 (3.13, 8.07)		50 (28%)	1.77 (1.18, 2.65)	
	MM	184	10 (5%)	0.67 (0.33, 1.39)		9 (4%)	0.22 (0.11, 0.46)	
Female		476	77 (16%)		< 0.001	95 (20%)		< 0.001
	NHL	215	21 (10%)			50 (23%)		
	ALL	131	46 (35%)	5.0 (2.81, 8.89)		37 (28%)	1.3 (0.79, 2.13)	
	MM	130	10 (7%)	0.77 (0.35, 1.69)		8 (6%)	0.22 (0.1, 0.47)	
**Vector Design**								
non-gCD28vec		906	112 (12%)		< 0.001	109 (12%)		< 0.001
	NHL	438	31 (7%)			59 (13%)		
	ALL	154	61 (39%)	8.61 (5.29, 14.02)		33 (21%)	1.75 (1.09, 2.81)	
	MM	314	20 (6%)	0.89 (0.5, 1.6)		17 (5%)	0.37 (0.21, 0.64)	
gCD28vec		371	62 (17%)		0.001	128 (35%)		0.697
	NHL	221	25 (11%)			74 (33%)		
	ALL	150	37 (24%)	2.57 (1.47, 4.48)		54 (36%)	1.12 (0.72, 1.73)	

Figure 2. Analysis of indication within select subgroups

Within each subgroup (as defined in the first column), rates of severe cytokine release syndrome (sCRS) and severe neurological toxicities (sNTX) were calculated for various indications (non-Hodgkin's lymphoma [NHL], acute lymphocytic leukemia [ALL], and multiple myeloma [MM]) and presented in the "Count (%)" column. The "Odds Ratio (95% CI)" columns contain odds ratios and 95% confidence intervals calculated using NHL as a reference point within each subgroup. Forest plots are displayed next to the "Odds Ratio (95% CI)" column to visually represent relative risk. The "P Value" column lists p values from chi-square tests comparing severe toxicity rates in each group. gCD28vec, products produced with gammaretrovirus vectors with CD28 sequences in the transgene; LGI, low- grade intervention; HGI, high-grade intervention.

copies/μg DNA, n = 8). Because of differences in measurement units, not all MM expansion data could be included in the analysis. Among ALL subjects, median maximum transgene concentration was higher in adult (62,532 transgene copies/μg DNA, n = 78) compared with pediatric (44,652 transgene copies/μg DNA, n = 243) subjects (p=0.004). However, in subgroup analysis, differences between adult and pediatric ALL subjects receiving products with the same costimulatory domain were not significant. Transgene concentration measurements during the first 36 h after CAR T cell administration were not available for most subjects and were therefore not examined.

Dosing parameters

Three dosing parameters (transduced cell count, transduction frequency, and total cell count) were analyzed for correlation with sCRS. Transduced cell count is the quantity of T cells in the final CAR T cell product that have been transduced with the CAR, transduction frequency is the ratio of transduced cells to total cells in the final CAR T cell product, and total cell count is the quantity of all cells in the final CAR T cell product. Although products within a study are manufactured to meet the sponsors' specified transduced cell dose, transduction frequency may vary widely among products because of the transduction rate of the patient-specific lot.

There was no statistically significant difference in transduced cell counts between subjects who experienced sCRS versus non-sCRS (Figure 5Ai). Subjects who experienced sCRS received products with lower transduction frequencies (Figure 5Aii). Subgroup analysis by indication found that for subjects with ALL or NHL, but not MM, transduction frequencies were significantly lower among subjects who experienced sCRS (Figure S4). Subgroup analysis indicated that for LGI subjects, transduction frequencies were lower for those who experienced sCRS, while there was no statistically significant difference among HGI subjects (results not shown). Although subjects who experienced sCRS received products with greater total cell counts (Figure 5Aiii), this may be driven by subjects in the HGI group (which

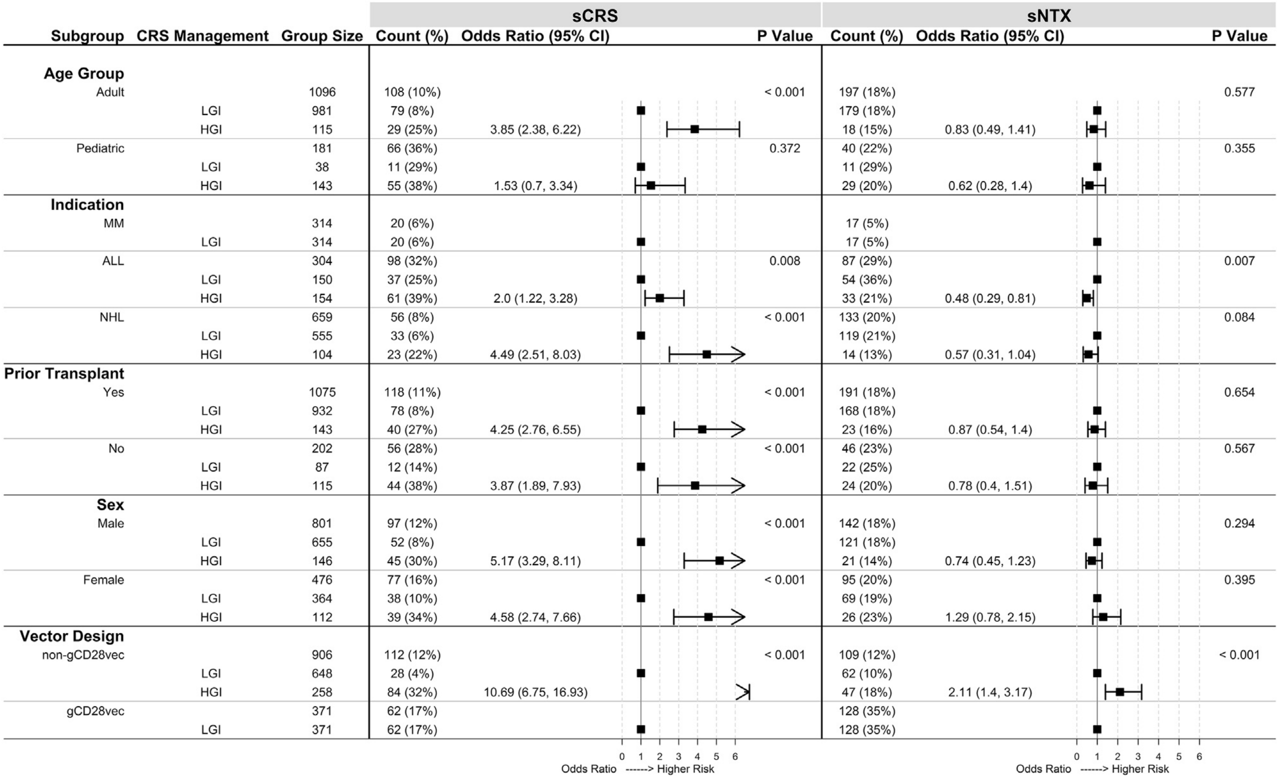

Subgroup	CRS Management	Group Size	sCRS Count (%)	sCRS Odds Ratio (95% CI)	sCRS P Value	sNTX Count (%)	sNTX Odds Ratio (95% CI)	sNTX P Value
**Age Group**								
Adult		1096	108 (10%)		< 0.001	197 (18%)		0.577
	LGI	981	79 (8%)			179 (18%)		
	HGI	115	29 (25%)	3.85 (2.38, 6.22)		18 (15%)	0.83 (0.49, 1.41)	
Pediatric		181	66 (36%)		0.372	40 (22%)		0.355
	LGI	38	11 (29%)			11 (29%)		
	HGI	143	55 (38%)	1.53 (0.7, 3.34)		29 (20%)	0.62 (0.28, 1.4)	
**Indication**								
MM		314	20 (6%)			17 (5%)		
	LGI	314	20 (6%)			17 (5%)		
ALL		304	98 (32%)		0.008	87 (29%)		0.007
	LGI	150	37 (25%)			54 (36%)		
	HGI	154	61 (39%)	2.0 (1.22, 3.28)		33 (21%)	0.48 (0.29, 0.81)	
NHL		659	56 (8%)		< 0.001	133 (20%)		0.084
	LGI	555	33 (6%)			119 (21%)		
	HGI	104	23 (22%)	4.49 (2.51, 8.03)		14 (13%)	0.57 (0.31, 1.04)	
**Prior Transplant**								
Yes		1075	118 (11%)		< 0.001	191 (18%)		0.654
	LGI	932	78 (8%)			168 (18%)		
	HGI	143	40 (27%)	4.25 (2.76, 6.55)		23 (16%)	0.87 (0.54, 1.4)	
No		202	56 (28%)		< 0.001	46 (23%)		0.567
	LGI	87	12 (14%)			22 (25%)		
	HGI	115	44 (38%)	3.87 (1.89, 7.93)		24 (20%)	0.78 (0.4, 1.51)	
**Sex**								
Male		801	97 (12%)		< 0.001	142 (18%)		0.294
	LGI	655	52 (8%)			121 (18%)		
	HGI	146	45 (30%)	5.17 (3.29, 8.11)		21 (14%)	0.74 (0.45, 1.23)	
Female		476	77 (16%)		< 0.001	95 (20%)		0.395
	LGI	364	38 (10%)			69 (19%)		
	HGI	112	39 (34%)	4.58 (2.74, 7.66)		26 (23%)	1.29 (0.78, 2.15)	
**Vector Design**								
non-gCD28vec		906	112 (12%)		< 0.001	109 (12%)		< 0.001
	LGI	648	28 (4%)			62 (10%)		
	HGI	258	84 (32%)	10.69 (6.75, 16.93)		47 (18%)	2.11 (1.4, 3.17)	
gCD28vec		371	62 (17%)			128 (35%)		
	LGI	371	62 (17%)			128 (35%)		

Figure 3. Analysis of CRS management protocol within select subgroups

Within each subgroup (as defined in the first column), rates of severe cytokine release syndrome (sCRS) and severe neurological toxicities (sNTX) were calculated for the low-grade intervention (LGI) protocol and the high-grade intervention (HGI) protocol and presented in the "Count (%)" column. The "Odds Ratio (95% CI)" column contains odds ratios and 95% confidence intervals calculated using the LGI group as a reference point within each subgroup. Forest plots are displayed next to the "Odds Ratio (95% CI)" column to visually represent relative risk. The "P Value" column lists p values from chi-square tests comparing severe toxicity rates in each group. ALL, acute lymphocytic leukemia; NHL, non-Hodgkin's lymphoma; MM, multiple myeloma; gCD28vec, products produced with gammaretrovirus vectors with CD28 sequences in the transgene.

reported higher rates of sCRS) who received products with greater total cell counts than LGI subjects (7.90×10^8 versus 1.92×10^8, $p < 0.001$, n = 1,254). Subgroup analysis within CRS management protocols (LGI versus HGI) revealed no statistically significant difference in median total cell count between subject who experienced sCRS versus non-sCRS.

Percentage T cells

Percentage T cells was not a differentiating factor for sCRS (Figure 6Ai).

Percentage viable cells

Although cell viability was >90% for both groups, subjects who experienced sCRS received products with higher percentages of viable cells (Figure 6Aii). Subgroup analysis by indication revealed that among NHL subjects, percentages of viable cells were higher among subjects who experienced sCRS (94.5% [n = 52 of 493] versus 89.3% [n = 493 of 545], $p < 0.001$), whereas there was no statistically significant difference among subjects with ALL (92.2% [n = 97 of 298] versus 90.0% [n = 201 of 298], p = 0.16) or MM (92.8% [n = 18 of 292] versus 91.3% [n = 274 of 292], p = 0.97). Subgroup analysis by CRS management protocol found that among LGI subjects, subjects who experienced sCRS received products with a higher percentage of viable cells compared with subjects who did not experience sCRS (92.5% [n = 84 of 886] versus 89.8% [n = 802 of 886], p = 0.002). Figure S5 provides additional analysis of the relationship between cell viability and sCRS for both indication and CRS management protocol.

Vector copy number

Because of different underlying assay methods and measurement units (e.g., per cell, per transduced cell, per amount of DNA), vector copy number (VCN) data were rank-normalized; VCN levels in each study were assigned a value between 0 and 1. VCN rank was not a differentiating factor for sCRS (Figure 6Aiii).

Potency

Because of different underlying assay methods and measurement units, product potency results (as measured by IFN-ɣ secretion assay) were rank-normalized in each study by assigning a value between 0 and 1. Product potency rank was not a differentiating factor for sCRS (Figure 6Aiv).

Subgroup	Vector Design	Group Size	sCRS Count (%)	sCRS Odds Ratio (95% CI)	sCRS P Value	sNTX Count (%)	sNTX Odds Ratio (95% CI)	sNTX P Value
**Age Group**								
Adult		1096	108 (10%)		< 0.001	197 (18%)		< 0.001
	non-gCD28vec	760	56 (7%)			79 (10%)		
	gCD28vec	336	52 (15%)	2.3 (1.54, 3.44)		118 (35%)	4.67 (3.38, 6.45)	
Pediatric		181	66 (36%)		0.376	40 (22%)		0.423
	non-gCD28vec	146	56 (38%)			30 (21%)		
	gCD28vec	35	10 (28%)	0.64 (0.29, 1.44)		10 (28%)	1.55 (0.67, 3.57)	
**CRS Intervention**								
LGI		1019	90 (9%)		< 0.001	190 (19%)		< 0.001
	non-gCD28vec	648	28 (4%)			62 (10%)		
	gCD28vec	371	62 (16%)	4.44 (2.79, 7.08)		128 (34%)	4.98 (3.55, 6.98)	
HGI		258	84 (33%)			47 (18%)		
	non-gCD28vec	258	84 (33%)			47 (18%)		
**Indication**								
MM		314	20 (6%)			17 (5%)		
	non-gCD28vec	314	20 (6%)			17 (5%)		
ALL		304	98 (32%)		0.008	87 (29%)		0.007
	non-gCD28vec	154	61 (40%)			33 (21%)		
	gCD28vec	150	37 (24%)	0.5 (0.31, 0.82)		54 (36%)	2.06 (1.24, 3.43)	
NHL		659	56 (8%)		0.091	133 (20%)		< 0.001
	non-gCD28vec	438	31 (7%)			59 (13%)		
	gCD28vec	221	25 (11%)	1.67 (0.96, 2.91)		74 (33%)	3.23 (2.19, 4.78)	
**Prior Transplant**								
Yes		1075	118 (11%)		0.001	191 (18%)		< 0.001
	non-gCD28vec	728	64 (9%)			80 (11%)		
	gCD28vec	347	54 (15%)	1.91 (1.3, 2.82)		111 (31%)	3.81 (2.76, 5.27)	
No		202	56 (28%)		0.681	46 (23%)		< 0.001
	non-gCD28vec	178	48 (27%)			29 (16%)		
	gCD28vec	24	8 (33%)	1.35 (0.54, 3.37)		17 (70%)	12.48 (4.75, 32.78)	
**Sex**								
Male		801	97 (12%)		0.309	142 (18%)		< 0.001
	non-gCD28vec	552	62 (11%)			57 (10%)		
	gCD28vec	249	35 (14%)	1.29 (0.83, 2.02)		85 (34%)	4.5 (3.08, 6.58)	
Female		476	77 (16%)		0.054	95 (20%)		< 0.001
	non-gCD28vec	354	50 (14%)			52 (15%)		
	gCD28vec	122	27 (22%)	1.73 (1.03, 2.91)		43 (35%)	3.16 (1.97, 5.08)	

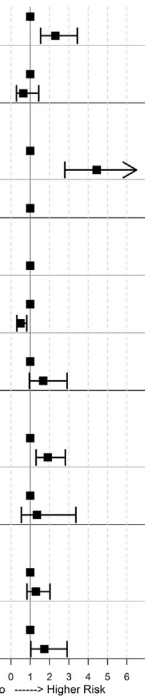

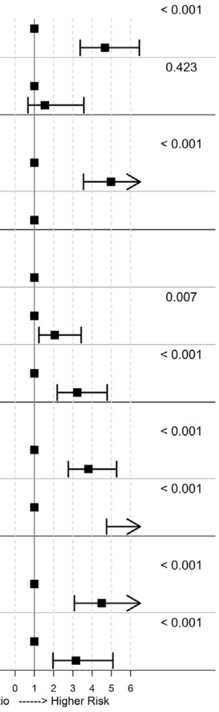

Figure 4. Analysis of vector design within select subgroups

Within each subgroup (as defined in the first column), rates of severe cytokine release syndrome (sCRS) and severe neurological toxicities (sNTX) were calculated for products produced with gammaretrovirus vectors with CD28 sequences in the transgene (gCD28vec) and products not produced with this vector design (non-gCD28vec) and presented in the "Count (%)" column. The "Odds Ratio (95% CI)" column contains odds ratios and 95% confidence intervals calculated using the non-gCD28vec group as a reference point within each subgroup. Forest plots are displayed next to the "Odds Ratio (95% CI)" column to visually represent relative risk. The "P Value" column lists p values from chi-square tests comparing severe toxicity rates in each group. ALL, acute lymphocytic leukemia; NHL, non-Hodgkin's lymphoma; MM, multiple myeloma; LGI, low-grade intervention; HGI, high-grade intervention.

NTX

Within the first 28 days after CAR T cell administration, 851 subjects (66.6%) experienced NTX, and 237 subjects (18.6%) experienced severe NTX (sNTX) (toxicity grade $\geq$ 3). Median onset time for the first sNTX was 5.6 days post-infusion.

Indication

Compared with subjects with NHL, subjects with ALL had higher rates of sNTX, while subjects with MM had lower rates of sNTX (Figure 1, "Indication"). Subgroup analyses indicated that ALL subjects had increased risk of sNTX compared with NHL subjects in certain subgroups (adults, LGI CRS management protocol, no prior transplantation, males, and subjects receiving gCD28vec; Figure 2). There was no statistically significant difference in sNTX rate between adult ALL and NHL subjects administered the same CD19-targetting CAR T cell product (Figure S1B). MM subjects had lower rates of sNTX than NHL subjects (Figure 2), except in subjects with no prior transplantation (Figure 2, "Prior Transplant: No").

CRS management protocol

There was no statistically significant difference in rates of sNTX between HGI and LGI subjects (Figure 1, "CRS Intervention"). Subgroup analysis supported this finding in all groups except ALL (Figure 3, "Indication: ALL") and non-gCD28vec (Figure 3, "Vector Design: non-gCD28vec"). In the ALL subgroup, this was an artifact of HGI subjects' consisting mostly of pediatrics who had lower rates of sNTX than adults (Figure S2B). Lower sNTX rates in the non-gCD28vec subgroup may be an artifact of LGI subjects' receiving a disproportionate number of BCMA products, which had lower rates of sNTX than CD19 products (Figure 1). After controlling for age group and target antigen in the ALL group and non-CD28vec group, respectively, there were no statistically significant differences in sNTX rate between HGI and LGI subjects (Figures S6 and S7).

Vector design

Subjects in the gCD28vec group had higher rates of sNTX (Figure 1, "Vector Design"). Higher rates of sNTX were maintained regardless of sex, prior transplant status, CRS management protocol, or

Table 1. Differences in cytokine levels for subjects with sCRS and sNTX

Cytokine	sCRS (36 h)	sCRS (before CRS)	sNTX (36 h)
CCL2	higher ($p < 0.001$, n = 582)	higher (p = 0.01, n = 787)	higher ($p < 0.001$, n = 582)
CCL3	NSD	NSD	lower ($p < 0.001$, n = 623)
CCL4	NSD	NSD	higher (p = 0.040, n = 627)
GMCSF	NSD	NSD	NSD
IFN-ɣ	higher (p = 0.01, n = 925)	higher (p = 0.03, n = 1,133)	NSD
IL-1β	NSD	NSD	NSD
IL-2	higher (p = 0.04, n = 913)	NSD	NSD
IL-4	lower ($p < 0.001$, n = 764)	lower ($p < 0.001$, n = 1,014)	higher (p = 0.014, n = 764)
IL-5	NSD	NSD	NSD
IL-6	NSD	NSD	NSD
IL-7	NSD	NSD	higher (p = 0.048, n = 600)
IL-8	higher (p = 0.001, n = 925)	NSD	higher (p = 0.023, n = 925)
IL-10	NSD	NSD	NSD
IL-12	NSD	lower ($p < 0.001$, n = 1,004)	NSD
IL-13	NSD	NSD	NSD
IL-15	NSD	NSD	NSD
TNF-α	NSD	NSD	NSD

Average maximum cytokines levels within 36 h after CAR T cell product administration in subjects with severe cytokine release syndrome (sCRS) and severe neurological toxicities (sNTX) compared with subjects with non-sCRS/non-sNTX. For subjects who experienced sCRS, average maximum cytokine concentrations were also calculated using all cytokine concentrations before CRS. NSD, no significant difference at p = 0.05.

indication (Figure 4). Among adults, the gCD28vec group had higher rates of sNTX; while among pediatric subjects, there was no difference in sNTX rate between the gCD28vec and non-gCD28vec group (Figure 4, "Age Group"). As noted above, most pediatric subjects (n = 177 of 181) had underlying ALL, and the pediatric gCD28vec group was relatively small (n = 35), therefore we cannot rule out product- or indication-specific factors.

Age group

Although pooled analysis revealed no statistically significant difference in sNTX rates between adults and pediatrics (Figure 1, "Age Group"), subgroup analysis of ALL subjects indicated that pediatrics had lower rates of sNTX than adults (Figure S2B, "Indication: ALL"). Although some subgroups show pediatrics with higher rates of sNTX (Figure S2B, "Vector Design: non-gCD28vec"), this was secondary to indication as pediatrics primarily had ALL, which was associated with higher rates of sNTX (Figures 1 and 2). Controlling for indication in the non-gCD28vec group revealed pediatrics associated with lower rates of sNTX (19.7% [n = 28 of 142] versus 41.7% [n = 5 of 12]).

Cytokines

Differences in cytokine levels between subjects who experienced sNTX and those who did not are reported in Tables 1 and S2 and Figure S8.

Expansion in vivo

Median maximum transgene concentration was greater in subjects who experienced sNTX compared with those who did not (71,273 versus 19,997 transgene copies/μg DNA, p = 0.002).

Dosing parameters

Although subjects who experienced sNTX received products with lower transduced cell counts (Figure 5Biv), this finding did not persist in subgroup analysis by either indication or vector design. Transduction frequency was not a differentiating factor for sNTX (Figure 5Bv). Although subjects who experienced sNTX received products with lower total cell counts (Figure 5Bvi), this may be due to subjects in the gCD28vec group (which reported higher rates of sNTX) who received products with lower total cell counts than non-CD28vec subjects (2.36×10^8 versus 3.00×10^8, $p < 0.001$, n = 1,254). Subgroup analysis by vector design revealed no statistically significant difference in median total cell count between sNTX and non-sNTX subjects in either the gCD28vec or non-gCD28vec groups.

Percentage T cells

Percentage T cells was not a differentiating factor for sNTX (Figure 6Bv).

Percentage viable cells

Percentage of viable cells was not a differentiating factor for sNTX (Figure 6Bvi).

Vector copy number

Subjects who experienced sNTX received products manufactured with a lower rank VCN compared with subjects who did not experience sNTX (Figure 6Bvii). In subgroup analysis by indication, ALL subjects with sNTX received products manufactured with a lower rank VCN (0.21 [n = 84 of 288] versus 0.29 [n = 204 of 288],

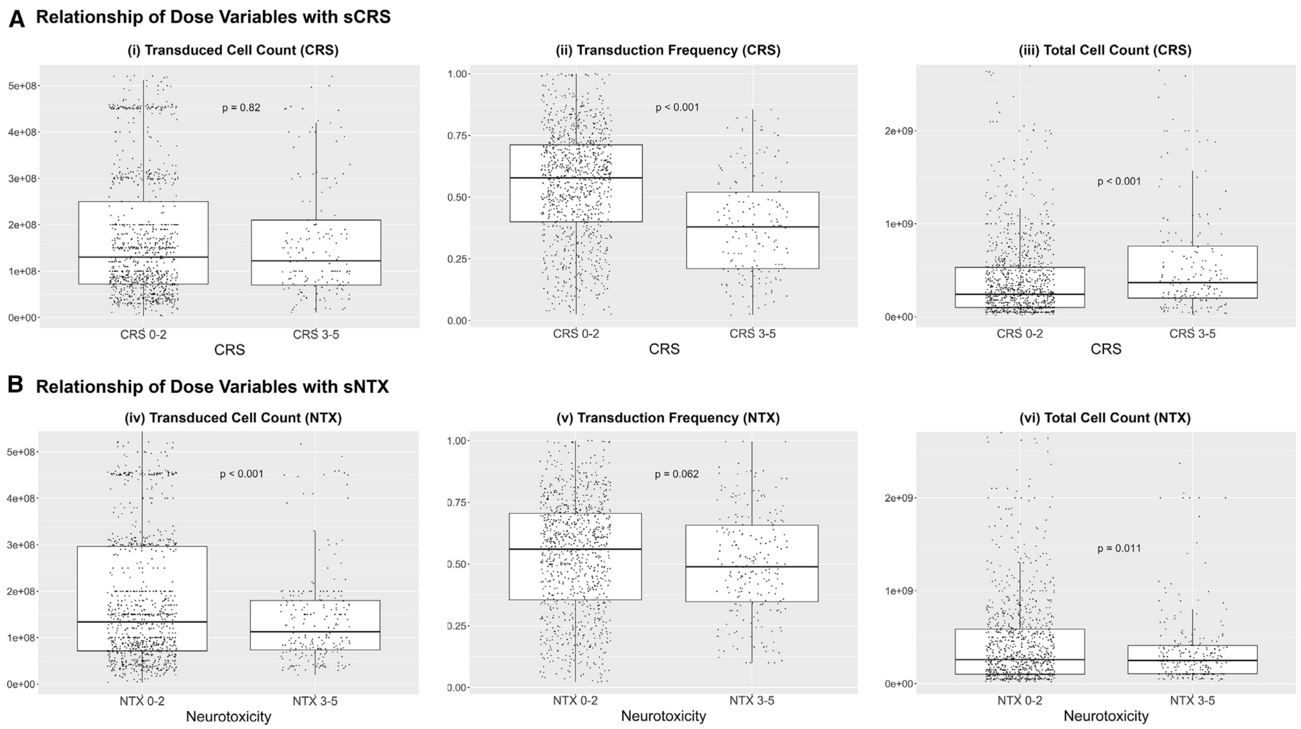

Figure 5. Relationship of dose variables with sCRS and sNTX

(Ai) There was no statistically significant difference in median transduced cell count for products administered to subjects with and without severe cytokine release syndrome (sCRS) (1.2×10^8 versus 1.3×10^8, n = 1,165). (Aii) Subjects with sCRS received products with smaller median transduction frequency (37.9% versus 57.8%, n = 1,150). (Aiii) Subjects with sCRS received products with greater median total cell counts (3.7×10^8 versus 2.4×10^8, n = 1,254). (Biv) Subjects with severe neurological toxicities (sNTX) received products with smaller median transduced cell counts (1.1×10^8 versus 1.3×10^8, n = 1,165). (Bv) There was no statistically significant difference in median transduction frequency for products administered to subjects with versus without sNTX (56.1% versus 48.9%, n = 1,150). (Bvi) Subjects with sNTX received products with smaller median total cell counts (2.5×10^8 versus 2.6×10^8, n = 1,254). Results were considered significant at $p \leq 0.05$.

p = 0.002), whereas there was no statistically significant difference in VCN rank for subjects with NHL (0.33 [n = 103 of 508] versus 0.34 [n = 405 of 508], p = 0.61) or MM (0.31 [n = 12 of 265] versus 0.33 [n = 253 of 265], p = 0.34).

Potency

Although subjects who experienced sNTX received higher rank potency products (Figure 6Bviii), this finding did not persist in subgroup analysis by indication for subjects with ALL (0.3 [n = 46 of 175] versus 0.26 [n = 129 of 175], p = 0.32), NHL (0.3 [n = 88 of 329] versus 0.24 [n = 241 of 329], p = 0.09), or MM (0.17 [n = 11 of 241] versus 0.14 [n = 230 of 214], p = 0.45).

Multivariate classification models

Multivariate models using data from all studies and all domains (clinical and CMC) were developed to identify which combination of factors show a strong association with the occurrence of sCRS or sNTX. Significant parameters for sCRS and sNTX are listed in Table 2. Forward-selected logistic regression models typically outperformed other classification methods on validation datasets. The best performing logistic regression model predicted the occurrence of sCRS with an accuracy of 73% and sensitivity of 80%, while the best model for predicting sNTX had an accuracy of 68% and sensitivity of 62%.

DISCUSSION

The first goal of this project was to assess the feasibility of integrating CAR T cell product data from multiple studies to enable cross-study data analysis. Although data formats varied among sponsors, the creation of a CAR T cell product-specific standard format and the development of data extraction, transformation, and loading tools allowed successful integration of cross-study data into a single database.

The second goal of this project was to perform exploratory analyses to validate the use of the integrated database for risk factor identification and predictive modeling. Although subgroup analysis and normalization methods were used to adjust for differences between studies, not all sources of confounding were able to be controlled for. As a result, risk factors identified in this exploratory analysis may not be directly related to sCRS/sNTX but instead may reflect fundamental differences in the included phase 1 and 2 trials.[20–23] In addition, as analyses were exploratory in nature, p values were not adjusted for multiple comparisons, further limiting the

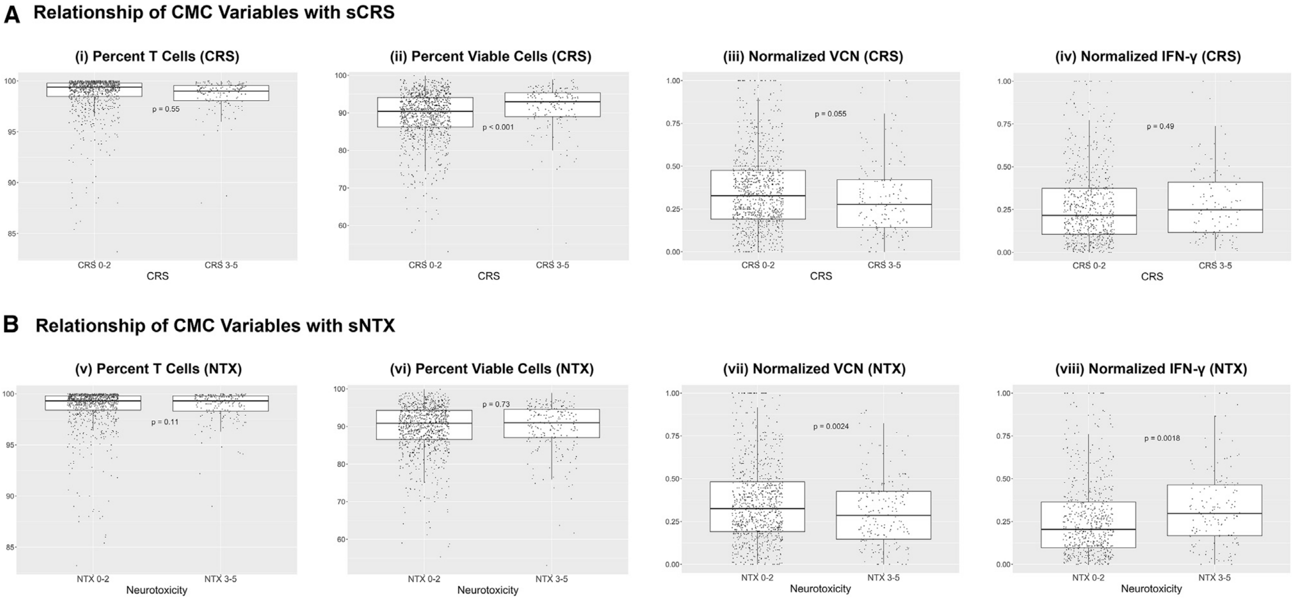

Figure 6. Relationship of cell manufacturing and control variables with sCRS and sNTX

(Ai) Percentage T cells was not a differentiating factor for severe cytokine release syndrome (sCRS) (99.0% versus 99.4%, n = 1,047). (Aii) Subjects with sCRS received products with greater percentage viable cells (92.3% versus 90.4%, n = 1,149). (Aiii) Rank-normalized vector copy number (VCN) was not a differentiating factor for sCRS (0.28 versus 0.33, n = 1,061). (Aiv) Rank-normalized interferon gamma (IFN-γ) was not a differentiating factor for sCRS (0.25 versus 0.22, n = 745). (Bv) Percentage T cells was not a differentiating factor for severe neurological toxicities (sNTX) (99.3% versus 99.3%, n = 1,047). (Bvi) Percentage viable cells was not a differentiating factor for sNTX (91.0% versus 90.9%, n = 1,149). (Bvii) Subjects with sNTX received products produced with lower rank-normalized VCN (0.29 versus 0.32, n = 1,061). (Bviii) Subjects with sNTX received products with greater rank-normalized IFN-γ (0.30 versus 0.20, n = 745). Results were considered significant at $p \leq 0.05$.

interpretation of results.[24] Therefore, findings should be interpreted as hypothesis generating and are not intended to support clinical or regulatory decisions.

Despite these limitations, several findings will be of interest to the CAR T cell clinical development community and may be considered when designing early phase dose-escalation cohorts. First, adults with ALL had a higher risk for developing both sCRS and sNTX compared with adults with either NHL or MM. Although evidence for the association of ALL with sNTX was not as strong as that for sCRS, it was still evident in multiple subgroup analyses. Although the association of ALL with sCRS and sNTX may be related to CAR T cell expansion *in vivo* (ALL subjects had substantially higher maximum transgene levels after administration), exploration of this hypothesis was beyond the scope of this analysis. A limitation of this analysis is that although disease burden was captured in our database, there was no meaningful way to standardize disease burden across different indications. As disease burden has been associated with both sCRS and sNTX, differences in underlying disease burden may have confounded this result.[13,14,25] Despite this limitation, our findings support work by others who have found that rates of sCRS are higher in patients with ALL than in those with NHL.[14,26–28] Thus, when considering the design of phase 1 CAR T cell therapy studies (particularly for CD19-targeted products), separate dose-escalation cohorts based on indication may be considered for determining maximum tolerable dose, which may be different among different indications with the same target.

Second, among subjects with ALL, pediatric subjects were less likely to experience sNTX and equally likely to experience sCRS as adult subjects. This finding supports work by others who have compared rates of sCRS and sNTX in adult versus pediatric ALL subjects.[29,30] Because of the limited number of pediatric subjects with NHL or MM in our database, it is unclear if these findings are applicable outside of ALL, although others have found age related associations with ICANS in adults with ALL and NHL.[31–34]

Third, studies using LGI with tocilizumab had lower rates of sCRS, with no difference in rates of sNTX. This finding supports the paradigm shift in CRS management that has been observed when comparing earlier studies with more recent ones, with the use of cytokine-directed therapies and corticosteroids at lower grades of toxicity leading to a significant reduction in sCRS with no evident impact on ICANS.[30,35–39] These findings suggest different risk mitigation strategies may be needed for CRS and ICANS.

This study reports several CAR T cell CMC findings. First, CAR T cells produced with gammaretrovirus vectors that included CD28 sequences in the CAR design appeared to increase the risk for sNTX. This finding corroborates other who have noted that subjects receiving CAR T cell products with CD28 domains have experienced higher levels of sNTX.[14,30,40] Because of the lack of structural variety in products produced using gammaretrovirus vectors, it was not possible to determine if the increased risk for sNTX was a result of vector type

Table 2. Significant parameters for multivariate logistic regression models

Predictors	Odds ratio	95% CI	p value
CRS (Grade 3–5)			
Maximum temperature (36 h)	1.44	1.26–1.64	<0.001
Maximum IL-4 concentration (36 h)	0.93	0.88–0.97	0.004
ALL indication	3.01	1.69–5.49	<0.001
gCD28vec products	3.96	1.55–12.21	0.008
HGI CRS management protocol	5.35	1.95–17.27	0.002
NTX (Grade 3–5)			
Maximum temperature (36 h)	1.43	1.27–1.61	<0.001
MM indication	0.17	0.08–0.35	<0.001
gCD28vec products	2.93	1.90–4.56	<0.001

Multivariate models using data from all studies and all domains were developed to identify which combination of factors show a strong association with the occurrence of severe cytokine release syndrome (CRS) or neurological toxicities (NTX). Model parameters were selected using forward selection using cross validation scores from a logistic regression estimator and selected parameters were fit using logistic regression with L1 regularization. Odds ratios and associated p values for significant variables were obtained from a logistic regression estimator. For CRS, indication, CRS intervention, vector design, temperature, IL-4, IL-8, and TNF-α were selected during parameter selection step. Temperature, IL-4 concentration, acute lymphocytic leukemia (ALL) indication, products produced with gammaretrovirus vectors with CD28 sequences in the transgene domain (gCD28vec), and high-grade intervention (HGI) CRS management protocol had significant effects on risk for severe CRS. For NTX, indication, vector design, temperature, CCL2, IL-8, IL-1β, and potency (IFN-γ) were selected during parameter selection step. Temperature, multiple myeloma (MM) indication, and gCD28vec vector design had significant effects on risk for severe NTX.

or CAR structure (Table S1). As vector design was typically uniform within a clinical study, the possibility of confounding factors unique to studies using this vector design cannot be ruled out.

Second, lower transduction frequency in the CAR T cell product was significantly associated with higher rates of sCRS and this relationship was observed in subjects grouped by both indication and study. This correlation could relate to the relationship between higher total cell counts and increased sCRS incidence, as lower transduction frequency would necessitate administration of a higher total number of cells to meet dose. This finding suggests that untransduced cells or cellular traits that contribute to lower transduction frequency could be a sCRS risk factor. Although others have found associations between T cell subset composition and toxicity, because neither the cellular composition of untransduced cells nor the T cell subsets were evaluated by most sponsors, we could not determine if particular cell types were associated with higher rates of sCRS.[13,41–45]

There were multiple challenges associated with interpreting results from this cross-study data analysis. The primary challenge was how to properly assess the trade-off between achieving greater statistical power for risk factor detection (by increasing the number of eligible subjects in the analysis) with the increase in potential confounders when combining datasets with different clinical and CMC profiles. Differences in the severity and frequency of CRS and NTX among subjects from different clinical and CMC subgroups (such as subjects with different cancers or different product profiles) may also have caused the analysis results to be confounded by factors unique to these subgroups. In addition, pertinent baseline characteristics such as tumor burden and inclusion/exclusion criteria across studies was not captured in our analysis and limits interpretation of results.

Although we tried to account for some sources of confounding by using subgroup analyses, differences in prognostic factors and limitations in the quantity of reported data between studies made it impossible to perform comparisons in a completely standardized way. Although disease burden data were sometimes available for leukemias, lymphomas, which require specifications for imaging techniques in addition to the standardized PET/CT-based imaging routinely used to evaluate response to therapy and disease status at study entry, were not available for most studies. Even if included, it would be difficult to interpret the results of an analysis evaluating the impact of disease burden on safety outcomes within and across indications, as differences in imaging parameters used to determine disease burden may also confound these results. Despite the limitations from confounding and missing data, this is the largest analysis of pooled data across CAR T cell products and results from this exploratory analysis can help researchers identify areas to test in more rigorously controlled populations.

A second major challenge was incorporating differences in grading and management strategies between studies. For CRS, differences in the Penn, Lee, and ASTCT criteria not only influence CRS toxicity grade but also the timing of tocilizumab administration, further complicating cross-study comparison.[10,46–50] Multiple steps were taken to control for and investigate how differences in grading systems affected our findings. Subgroup analyses were performed within studies using the same grading systems to test the robustness of pooled results. We also repeated our analysis with sCRS defined as toxicity grade $\geq$ 4 (the grade at which different grading criteria converge in assessment) and found the primary findings of our analysis remained consistent (results not shown). We also investigated the algorithmic conversion between grading criteria and again found the primary findings of our analysis remained consistent (results not shown). These complementary approaches lend validity to our exploratory findings, despite variations in the CRS grading systems.

For NTX, outcomes were standardized using Medical Dictionary for Regulatory Activities (MedDRA) system organ classes. Despite not taking a more selective approach for classifying neurological events (e.g., using the ICANS definition), almost all studies used the same version of the Common Terminology Criteria for Adverse Events (CTCAE), and rates of sNTX were consistent with listed rates in the package insert of marketed products.[51–55] Therefore this standardization strategy allowed accurate comparisons between studies in this exploratory analysis, although we do not recommend this approach for use in clinical trial design or decision making.

This study showed that although it is feasible to combine cross-study safety data for analysis, data standardization and differences in study design complicate analysis and interpretation of results. Although

standardization efforts in the field may facilitate future efforts to assess safety risks across products, CAR T cell products are still an evolving therapeutic class and adherence to specified standards may not be feasible or desirable at this time. Although this study focuses specifically on risk factors associated with CRS and NTX, future analyses could consider additional safety factors and emerging toxicities (e.g., prolonged cytopenia, macrophage activation syndrome, or hemophagocytic lymphohistiocytosis).[56,57] Findings from these exploratory analyses offer researchers and stakeholders in the CAR T cell clinical development community information on broader safety trends, considerations for early phase trial design, and avenues for future research.

MATERIALS AND METHODS

Proprietary and confidential clinical safety and manufacturing data from 17 early to late phase studies received prior to September 1, 2020, were voluntarily provided by investigational new drug (IND) application sponsors for incorporation into the CAR T cell safety database.[17] Studies were selected by asking sponsors with relatively large numbers of patients in CAR T cell trials to voluntarily contribute data for cross-study safety analysis. Such data, unlike submissions for licensing applications, were not cleaned or locked. Demographics, adverse events, concomitant medications, serum cytokine profiles, dosing regimen, cellular kinetic profiles, and CMC information were included in the CAR T cell safety database. Most data were provided in SAS transport files and followed the Clinical Data Interchange Standards Consortium (CDISC) Study Data Tabulation Model (SDTM).[58] All data voluntarily submitted for this project were included in the analysis.

To integrate cross-study data into a standardized database, a custom data schema was designed based on the CDISC SDTM format. Software tools were developed to transform sponsor-submitted data to meet the requirements of the integrated database. As data were received, the initial data schema was updated to incorporate additional CAR T cell-specific information (e.g., cell population metrics). Reference tables were also developed to standardize terminology and reduce inconsistencies in the database. Additional information on data standardization (Notes S1–S5), as well as the final schema for the CAR T cell safety database (Appendix I), can be found in the supplemental information.

Occurrence of sCRS or sNTX within 28 days after CAR T cell product administration were the primary safety outcomes of interest. A time window of 28 days after product administration was selected to focus our analysis on short-term toxicities likely due to product administration and not related to disease progression. Subjects were classified as experiencing sCRS if they experienced at least one occurrence of CRS at toxicity grade $\geq$ 3 within 28 days after CAR T cell product administration. CRS toxicity grades were not reviewed for accuracy or adherence to the sponsor's stated grading criteria and were accepted as reported. NTX was broadly defined as the occurrence of one or more adverse events under the neurologic disorders or psychiatric disorders Medical Dictionary for Regulatory Activities 20.1 system organ class.[59] Subjects were classified as experiencing sNTX if they experienced at least one occurrence of NTX at toxicity grade $\geq$ 3 within 28 days after CAR T cell product administration. For subjects who had multiple episodes of CRS or NTX, only the maximum CRS or NTX toxicity grade was used for analysis. The most common adverse events used to define both NTX and sNTX are listed in Tables S3 and S4.

After integration of cross-study safety data, risk factors analysis was performed separately for sCRS and sNTX. Risk factors analyzed included indication (ALL versus NHL versus MM), CRS management protocol (HGI versus LGI; Note S6), vector design (gCD28vec versus non-gCD28vec), age group (adult versus pediatric; Note S7), cytokines (maximum concentration in blood within 36 h post-CAR T cell product infusion), expansion *in vivo* (maximum concentration of CAR T cell transgene copies in blood within 28 days post-CAR T cell product infusion), dosing parameters (transduced cell count, transduction frequency, and total cell count), and CMC characteristics (percentage T cells, percentage viable cells, vector copy number, and potency).

Bivariate analysis of continuous data was performed by splitting subjects into two groups on the basis of adverse event severity (i.e., severe versus non-severe) and using t tests to determine significant differences between groups ($p \leq 0.05$). Bivariate analysis of categorical data used the chi-square test to test for differences in the percentages of subjects with sCRS or sNTX in different subgroups ($p \leq 0.05$). For select variables, odds ratios and 95% confidence intervals were calculated to evaluate comparative risk. Initial bivariate analysis was followed by subgroup analyses to rule out potential confounding factors. For cases in which integration of numerical data from across studies was not possible, because of differences in units or underlying assay methods (e.g., if two studies used different target cells for IFN-γ secretion assays), data were either rank-normalized to values between 0 and 1 within each study or dropped from analysis. Despite the potential for different underlying assay methods, cytokine data were not rank normalized, as comparisons of cytokine distributions between studies were comparable after converting all measurements to picograms per milliliter.

Multivariate models such as logistic regression, decision trees, and random forests were developed to identify which combination of factors show a strong association with the occurrence of sCRS or sNTX. Inputs to these models included demographics data (including indication), CMC data (IFN-γ, VCN, cellular composition data, and vector design data), dose data (total cell count, transduced cell count, percentage transduction, total cell count per kilogram, and transduced cell count per kg), CRS management protocol, maximum cytokine concentrations within 36 h after CAR T cell administration, and maximum temperature within 36 h after CAR T cell administration (Note S8). All multivariate models were developed using training data and validated on data not used during model training. As cytokine data were typically submitted in a timeseries format with sparse measurements at irregular time points, cytokine data were interpolated to be compatible with multivariate analysis algorithms (Note S9).

DATA AVAILABILITY

The data that support the findings of this study are not openly available. Confidentiality agreements with sponsors who voluntarily

provided clinical safety and manufacturing for this project prohibit data sharing.

SUPPLEMENTAL INFORMATION

Supplemental information can be found online at https://doi.org/10.1016/j.omto.2022.10.006.

ACKNOWLEDGMENTS

We would like to thank all the sponsors and patients who contributed data for this project, this project would not have been possible without their contributions. We would also like to thank Nirjal Bhattarai, Arya Eskandarian, Richard Forshee, Chao Liu, Donna Monk, Graeme Price, John Scott, Mark Walderhaug, Wei Wang, and Yaning Wang, who provided feedback and expertise at various stages throughout the project. CBER/FDA provided funding for these studies as part of its intramural research program. The graphical abstract was created using BioRender.com.

AUTHOR CONTRIBUTIONS

Conceptualization, B.G., K.S., X.W., and W.W.B.; Methodology, Y.N. and M.F.; Software, Y.N. and M.F.; Validation; Y.N. and M.F.; Formal Analysis, Y.N.; Data Curation, Y.N. and M.F.; Writing – Original Draft, M.F.; Writing – Review and Editing, M.F., Y.N., L.E., W.W.B., X.W., Y.X., K.S., and B.G.; Visualization, Y.N. and Y.X.; Supervision, B.G., K.S., X.W., and W.W.B.; Project Administration, L.E.; Funding Acquisition, B.G. and W.W.B.

DECLARATION OF INTERESTS

The authors declare no competing interests.

REFERENCES

1. U.S. Food & Drug Administration (2022). FDA Approves Ciltacabtagene Autoleucel for Relapsed or Refractory Multiple Myeloma. https://www.fda.gov/drugs/resources-information-approved-drugs/fda-approves-ciltacabtagene-autoleucel-relapsed-or-refractory-multiple-myeloma.

2. U.S. Food & Drug Administration (2020). FDA Approves Brexucabtagene Autoleucel for Relapsed or Refractory Mantle Cell Lymphoma. https://www.fda.gov/drugs/fda-approves-brexucabtagene-autoleucel-relapsed-or-refractory-mantle-cell-lymphoma.

3. U.S. Food & Drug Administration (2021). FDA Approves Lisocabtagene Maraleucel for Relapsed or Refractory Large B-Cell Lymphoma. https://www.fda.gov/drugs/drug-approvals-and-databases/fda-approves-lisocabtagene-maraleucel-relapsed-or-refractory-large-b-cell-lymphoma.

4. U.S. Food & Drug Administration (2021). FDA Approves Idecabtagene Vicleucel for Multiple Myeloma. https://www.fda.gov/drugs/drug-approvals-and-databases/fda-approves-idecabtagene-vicleucel-multiple-myeloma.

5. U.S. Food & Drug Administration (2017). FDA Approval Brings First Gene Therapy to the United States. https://www.fda.gov/news-events/press-announcements/fda-approval-brings-first-gene-therapy-united-states.

6. U.S. Food & Drug Administration (2017). FDA Approves Axicabtagene Ciloleucel for Large B-Cell Lymphoma. https://www.fda.gov/drugs/resources-information-approved-drugs/fda-approves-axicabtagene-ciloleucel-large-b-cell-lymphoma.

7. Murthy, H., Iqbal, M., Chavez, J.C., and Kharfan-Dabaja, M.A. (2019). Cytokine release syndrome: current perspectives. Immunotargets Ther. *8*, 43–52. https://doi.org/10.2147/ITT.S202015.

8. Shimabukuro-Vornhagen, A., Gödel, P., Subklewe, M., Stemmler, H.J., Schlößer, H.A., Schlaak, M., Kochanek, M., Böll, B., and von Bergwelt-Baildon, M.S. (2018). Cytokine release syndrome. J. Immunother. Cancer *6*, 56. https://doi.org/10.1186/s40425-018-0343-9.

9. Gust, J., Hay, K.A., Hanafi, L.A., Li, D., Myerson, D., Gonzalez-Cuyar, L.F., Yeung, C., Liles, W.C., Wurfel, M., Lopez, J.A., et al. (2017). Endothelial activation and blood-brain barrier disruption in neurotoxicity after adoptive immunotherapy with CD19 CAR-T cells. Cancer Discov. *7*, 1404–1419. https://doi.org/10.1158/2159-8290.Cd-17-0698.

10. Lee, D.W., Santomasso, B.D., Locke, F.L., Ghobadi, A., Turtle, C.J., Brudno, J.N., Maus, M.V., Park, J.H., Mead, E., Pavletic, S., et al. (2019). ASTCT consensus grading for cytokine release syndrome and neurologic toxicity associated with immune effector cells. Biol. Blood Marrow Transpl. *25*, 625–638. https://doi.org/10.1016/j.bbmt.2018.12.758.

11. Chou, C.K., and Turtle, C.J. (2020). Assessment and management of cytokine release syndrome and neurotoxicity following CD19 CAR-T cell therapy. Expert Opin. Biol. Ther. *20*, 653–664. https://doi.org/10.1080/14712598.2020.1729735.

12. Teachey, D.T., Lacey, S.F., Shaw, P.A., Melenhorst, J.J., Maude, S.L., Frey, N., Pequignot, E., Gonzalez, V.E., Chen, F., Finklestein, J., et al. (2016). Identification of predictive biomarkers for cytokine release syndrome after chimeric antigen receptor T-cell therapy for acute lymphoblastic leukemia. Cancer Discov. *6*, 664–679. https://doi.org/10.1158/2159-8290.Cd-16-0040.

13. Hay, K.A., Hanafi, L.A., Li, D., Gust, J., Liles, W.C., Wurfel, M.M., López, J.A., Chen, J., Chung, D., Harju-Baker, S., et al. (2017). Kinetics and biomarkers of severe cytokine release syndrome after CD19 chimeric antigen receptor-modified T-cell therapy. Blood *130*, 2295–2306. https://doi.org/10.1182/blood-2017-06-793141.

14. Brudno, J.N., and Kochenderfer, J.N. (2019). Recent advances in CAR T-cell toxicity: mechanisms, manifestations and management. Blood Rev. *34*, 45–55. https://doi.org/10.1016/j.blre.2018.11.002.

15. Tedesco, V.E., 5th, and Mohan, C. (2021). Biomarkers for predicting cytokine release syndrome following CD19-targeted CAR T cell therapy. J. Immunol. *206*, 1561–1568. https://doi.org/10.4049/jimmunol.2001249.

16. Greenbaum, U., Strati, P., Saliba, R.M., Torres, J., Rondon, G., Nieto, Y., Hosing, C., Srour, S.A., Westin, J., Fayad, L.E., et al. (2021). CRP and ferritin in addition to the EASIX score predict CAR-T–related toxicity. Blood Adv. *5*, 2799–2806. https://doi.org/10.1182/bloodadvances.2021004575.

17. Thompson Reuters. FDA Proposes New Databases to Monitor CAR T-Cell Safety across INDs (US). Practical Law Life Sciences W-001-6501. https://uk.practicallaw.thomsonreuters.com/w-001-6501.

18. Wei, J., Liu, Y., Wang, C., Zhang, Y., Tong, C., Dai, G., Wang, W., Rasko, J.E.J., Melenhorst, J.J., Qian, W., et al. (2020). The model of cytokine release syndrome in CAR T-cell treatment for B-cell non-Hodgkin lymphoma. Signal Transduct. Target. Ther. *5*, 134. https://doi.org/10.1038/s41392-020-00256-x.

19. Wang, Z., and Han, W. (2018). Biomarkers of cytokine release syndrome and neurotoxicity related to CAR-T cell therapy. Biomark. Res. *6*, 4. https://doi.org/10.1186/s40364-018-0116-0.

20. Kim, H., Gurrin, L., Ademi, Z., and Liew, D. (2014). Overview of methods for comparing the efficacies of drugs in the absence of head-to-head clinical trial data. Br. J. Clin. Pharmacol. *77*, 116–121. https://doi.org/10.1111/bcp.12150.

21. Greenland, S. (1987). Quantitative methods in the review of epidemiologic literature. Epidemiol. Rev. *9*, 1–30. https://doi.org/10.1093/oxfordjournals.epirev.a036298.

22. Haidich, A.B. (2010). Meta-analysis in medical research. Hippokratia *14* (*Suppl 1*), 29–37.

23. Tierney, J.F., Stewart, L.A., and Clarke, M.; on behalf of the Cochrane Individual Participant Data Meta-analysis Methods Group (2019). Individual participant data. Cochrane Handbook Syst. Rev. Interventions, 643–658.

24. Jafari, M., and Ansari-Pour, N. (2019). Why, when and how to adjust your P values? Cell J. *20*, 604–607. https://doi.org/10.22074/cellj.2019.5992.

25. Chavez, J.C., Bachmeier, C., and Kharfan-Dabaja, M.A. (2019). CAR T-cell therapy for B-cell lymphomas: clinical trial results of available products. Ther. Adv. Hematol. *10*. 2040620719841581. https://doi.org/10.1177/2040620719841581.

26. Teachey, D.T., Bishop, M.R., Maloney, D.G., and Grupp, S.A. (2018). Toxicity management after chimeric antigen receptor T cell therapy: one size does not fit 'ALL. Nat. Rev. Clin. Oncol. *15*, 218. https://doi.org/10.1038/nrclinonc.2018.19.

27. Maude, S.L., Laetsch, T.W., Buechner, J., Rives, S., Boyer, M., Bittencourt, H., Bader, P., Verneris, M.R., Stefanski, H.E., Myers, G.D., et al. (2018). Tisagenlecleucel in children and young adults with B-cell lymphoblastic leukemia. N. Engl. J. Med. *378*, 439–448. https://doi.org/10.1056/NEJMoa1709866.

28. Schuster, S.J., Bishop, M.R., Tam, C.S., Waller, E.K., Borchmann, P., McGuirk, J.P., Jäger, U., Jaglowski, S., Andreadis, C., Westin, J.R., et al. (2019). Tisagenlecleucel in adult relapsed or refractory diffuse large B-cell lymphoma. N. Engl. J. Med. *380*, 45–56.

29. Anagnostou, T., Riaz, I.B., Hashmi, S.K., Murad, M.H., and Kenderian, S.S. (2019). CD19 directed chimeric antigen receptor T cell therapy in acute lymphoblastic leukemia: a systematic review and meta-analysis. Biol. Blood Marrow Transplant. *25*, S169–S170. https://doi.org/10.1016/j.bbmt.2018.12.308.

30. Gauthier, J., and Turtle, C.J. (2021). Chimeric antigen receptor T-cell therapy for B-cell acute lymphoblastic leukemia: current landscape in 2021. Cancer J. *27*, 98–106. https://doi.org/10.1097/ppo.0000000000000508.

31. Grant, S.J., Grimshaw, A.A., Silberstein, J., Murdaugh, D., Wildes, T.M., Rosko, A.E., and Giri, S. (2022). Clinical presentation, risk factors, and outcomes of immune effector cell-associated neurotoxicity syndrome following chimeric antigen receptor T cell therapy: a systematic review. Transpl. Cell. Ther. *28*, 294–302. https://doi.org/10.1016/j.jtct.2022.03.006.

32. Santomasso, B.D., Park, J.H., Salloum, D., Riviere, I., Flynn, J., Mead, E., Halton, E., Wang, X., Senechal, B., Purdon, T., et al. (2018). Clinical and biological correlates of neurotoxicity associated with CAR T-cell therapy in patients with B-cell acute lymphoblastic leukemia. Cancer Discov. *8*, 958–971. https://doi.org/10.1158/2159-8290.Cd-17-1319.

33. Rubin, D.B., Al Jarrah, A., Li, K., LaRose, S., Monk, A.D., Ali, A.B., Spendley, L.N., Nikiforow, S., Jacobson, C., and Vaitkevicius, H. (2020). Clinical predictors of neurotoxicity after chimeric antigen receptor T-cell therapy. JAMA Neurol. *77*, 1536–1542. https://doi.org/10.1001/jamaneurol.2020.2703.

34. Gauthier, J., Cearley, A., Perkins, P., Kirk, A., Shadman, M., Williamson, S., Myers, J., Chen, A.I., Nagle, S., Hayes-Lattin, B.M., et al. (2021). CD19 CAR T-cell product type independently impacts CRS and ICANS severity in patients with aggressive NHL. J. Clin. Oncol. *39*, 7532. https://doi.org/10.1200/JCO.2021.39.15_suppl.7532.

35. Caimi, P.F., Pacheco Sanchez, G., Sharma, A., Otegbeye, F., Ahmed, N., Rojas, P., Patel, S., Kleinsorge Block, S., Schiavone, J., Zamborsky, K., et al. (2021). Prophylactic tocilizumab prior to anti-CD19 CAR-T cell therapy for non-hodgkin lymphoma. Front. Immunol. *12*, 745320. https://doi.org/10.3389/fimmu.2021.745320.

36. Oluwole, O.O., Bouabdallah, K., Muñoz, J., De Guibert, S., Vose, J.M., Bartlett, N.L., Lin, Y., Deol, A., McSweeney, P.A., Goy, A.H., et al. (2021). Prophylactic corticosteroid use in patients receiving axicabtagene ciloleucel for large B-cell lymphoma. Br. J. Haematol. *194*, 690–700. https://doi.org/10.1111/bjh.17527.

37. Gardner, R., Leger, K.J., Annesley, C.E., Summers, C., Rivers, J., Gust, J., Tarlock, K., Cooper, T.M., Pinto, N.R., Finney, O., et al. (2016). Decreased rates of severe CRS seen with early intervention strategies for CD19 CAR-T cell toxicity management. Blood *128*, 586. https://doi.org/10.1182/blood.V128.22.586.586.

38. Gardner, R.A., Ceppi, F., Rivers, J., Annesley, C., Summers, C., Taraseviciute, A., Gust, J., Leger, K.J., Tarlock, K., Cooper, T.M., et al. (2019). Preemptive mitigation of CD19 CAR T-cell cytokine release syndrome without attenuation of antileukemic efficacy. Blood *134*, 2149–2158. https://doi.org/10.1182/blood.2019001463.

39. Kadauke, S., Maude, S., Gladney, W., Motley, L., Shenoy, V., Callahan, C., Baniewicz, D., Teachey, D.T., Grupp, S., and DiNofia, A. (2019). Early administration of tocilizumab (Toci) for the prevention of grade 4 cytokine release syndrome (CRS) after CD19-directed CAR T-cell therapy (CTL019). Cytotherapy *21*, e2–e3. https://doi.org/10.1016/j.jcyt.2019.04.009.

40. Cappell, K.M., and Kochenderfer, J.N. (2021). A comparison of chimeric antigen receptors containing CD28 versus 4-1BB costimulatory domains. Nat. Rev. Clin. Oncol. *18*, 715–727. https://doi.org/10.1038/s41571-021-00530-z.

41. Sommermeyer, D., Hudecek, M., Kosasih, P.L., Gogishvili, T., Maloney, D.G., Turtle, C.J., and Riddell, S.R. (2016). Chimeric antigen receptor-modified T cells derived from defined CD8+ and CD4+ subsets confer superior antitumor reactivity in vivo. Leukemia *30*, 492–500. https://doi.org/10.1038/leu.2015.247.

42. Singh, N., Perazzelli, J., Grupp, S.A., and Barrett, D.M. (2016). Early memory phenotypes drive T cell proliferation in patients with pediatric malignancies. Sci. Transl. Med. *8*, 320ra3. https://doi.org/10.1126/scitranslmed.aad5222.

43. Turtle, C.J., Hanafi, L.A., Berger, C., Hudecek, M., Pender, B., Robinson, E., Hawkins, R., Chaney, C., Cherian, S., Chen, X., et al. (2016). Immunotherapy of non-Hodgkin's lymphoma with a defined ratio of CD8+ and CD4+ CD19-specific chimeric antigen receptor-modified T cells. Sci. Transl. Med. *8*, 355ra116. https://doi.org/10.1126/scitranslmed.aaf8621.

44. Shah, N.N., Highfill, S.L., Shalabi, H., Yates, B., Jin, J., Wolters, P.L., Ombrello, A., Steinberg, S.M., Martin, S., Delbrook, C., et al. (2020). CD4/CD8 T-Cell selection affects chimeric antigen receptor (CAR) T-cell potency and toxicity: updated results from a phase I anti-CD22 CAR T-cell trial. J. Clin. Oncol. *38*, 1938–1950. https://doi.org/10.1200/jco.19.03279.

45. Arcangeli, S., Bove, C., Mezzanotte, C., Camisa, B., Falcone, L., Manfredi, F., Bezzecchi, E., El Khoury, R., Norata, R., Sanvito, F., et al. (2022). CAR T-cell manufacturing from naive/stem memory T-lymphocytes enhances antitumor responses while curtailing cytokine release syndrome. J. Clin. Invest. *132*, e150807. https://doi.org/10.1172/jci150807.

46. Lee, D.W., Gardner, R., Porter, D.L., Louis, C.U., Ahmed, N., Jensen, M., Grupp, S.A., and Mackall, C.L. (2014). Current concepts in the diagnosis and management of cytokine release syndrome. Blood *124*, 188–195. https://doi.org/10.1182/blood-2014-05-552729.

47. Porter, D., Frey, N., Wood, P.A., Weng, Y., and Grupp, S.A. (2018). Grading of cytokine release syndrome associated with the CAR T cell therapy tisagenlecleucel. J. Hematol. Oncol. *11*, 35. https://doi.org/10.1186/s13045-018-0571-y.

48. Locke, F.L., Ghobadi, A., Jacobson, C.A., Miklos, D.B., Lekakis, L.J., Oluwole, O.O., Lin, Y., Braunschweig, I., Hill, B.T., Timmerman, J.M., et al. (2019). Long-term safety and activity of axicabtagene ciloleucel in refractory large B-cell lymphoma (ZUMA-1): a single-arm, multicentre, phase 1-2 trial. Lancet Oncol. *20*, 31–42. https://doi.org/10.1016/s1470-2045(18)30864-7.

49. Schuster, S.J., Maziarz, R.T., Rusch, E.S., Li, J., Signorovitch, J.E., Romanov, V.V., Locke, F.L., and Maloney, D.G. (2020). Grading and management of cytokine release syndrome in patients treated with tisagenlecleucel in the JULIET trial. Blood Adv. *4*, 1432–1439. https://doi.org/10.1182/bloodadvances.2019001304.

50. Schuster, S.J., Bishop, M.R., Tam, C.S., Waller, E.K., Borchmann, P., McGuirk, J.P., Jäger, U., Jaglowski, S., Andreadis, C., Westin, J.R., et al. (2019). Tisagenlecleucel in adult relapsed or refractory diffuse large B-cell lymphoma. N. Engl. J. Med. *380*, 45–56. https://doi.org/10.1056/NEJMoa1804980.

51. Kite Pharma Incorporated. YESCARTA®(axicabtagene Ciloleucel). https://dailymed.nlm.nih.gov/dailymed/drugInfo.cfm?setid=9b70606e-b99c-4272-a0f1-b5523cce0c59.

52. Novartis Pharmaceuticals Corporation. KYMRIAH (Tisagenlecleucel). https://dailymed.nlm.nih.gov/dailymed/drugInfo.cfm?setid=aad3ba54-dfd3-4cb3-9e2b-c5ef89559189.

53. Corporation, C.. ABECMA®(idecabtagene Vicleucel). https://dailymed.nlm.nih.gov/dailymed/drugInfo.cfm?setid=b90c1fe7-f5cc-464e-958a-af36e9c26d7c.

54. Juno Therapeutics Incorporated. BREYANZI® (lisocabtagene maraleucel). https://dailymed.nlm.nih.gov/dailymed/drugInfo.cfm?setid=594bb413-af3b-4b97-afb3-bfe2b174f2ed.

55. Kite Pharma Incorporated. TECARTUS™(brexucabtagene Autoleuce). https://dailymed.nlm.nih.gov/dailymed/drugInfo.cfm?setid=a16108c2-7ca7-45af-965e-54bda4713022.

56. Martín-Rojas, R.M., Gómez-Centurión, I., Bailén, R., Bastos, M., Diaz-Crespo, F., Carbonell, D., Correa-Rocha, R., Pion, M., Muñoz, C., Sancho, M., et al. (2022). Hemophagocytic lymphohistiocytosis/macrophage activation syndrome (HLH/MAS) following treatment with tisagenlecleucel. Clin. Case Rep. *10*, e05209. https://doi.org/10.1002/ccr3.5209.

57. Sandler, R.D., Tattersall, R.S., Schoemans, H., Greco, R., Badoglio, M., Labopin, M., Alexander, T., Kirgizov, K., Rovira, M., Saif, M., et al. (2020). Diagnosis and management of secondary HLH/MAS following HSCT and CAR-T cell therapy in adults; A review of the literature and a survey of practice within EBMT centres on behalf of the autoimmune diseases working party (ADWP) and transplant complications working party (TCWP). Front. Immunol. *11*, 524. https://doi.org/10.3389/fimmu.2020.00524.

58. National Cancer Institute. CDISC terminology. https://datascience.cancer.gov/resources/cancer-vocabulary/cdisc-terminology.

59. MedDRA. MedDRA Hierarchy. https://www.meddra.org/how-to-use/basics/hierarchy.

OMTO, Volume *27*

Supplemental information

Cross-study safety analysis of risk factors

in CAR T cell clinical trials:

An FDA database pilot project

Matthew Foster, Yonatan Negash, Leslie Eberhardt, Wilson W. Bryan, Kimberly Schultz, Xiaofei Wang, Yuan Xu, and Bindu George

Supplemental Materials

Supplemental Notes

Note S1: Data Standardization

Multiple reference tables were developed to standardize information across various domains, this included the adverse events, concomitant medications, disease identification, response grading, cell types, cellular markers, and cytokines/biomolecules reference tables. Further information on standardization efforts in the adverse events (Note S2) and concomitant medication (Note S3) domains can be found below.

Note S2: Adverse Event Data Standardization

Adverse event standardization required standardizing adverse event terms using the adverse event reference table, as well as labeling adverse events using an adverse event mapping dictionary. The Adverse Event reference table was built using the Medical Dictionary for Regulatory Activities (MedDRA) version 20.1 and allowed mapping between MedDRA preferred terms, lower-level terms, and system organ class entries in the adverse event domain. While a natural language processing (NLP) tool was initially developed to standardize adverse events to MedDRA preferred terms, we found that 98.8% of terms could be directly mapped using dictionary look up methods and therefore the added value of using NLP to improve dictionary mapping was offset by the time taken to perform these processes.

Note S3: Concomitant Medication Data Standardization

The concomitant medication table was built around the World Health Organization (WHO) B3 Drug Dictionary from March 2020 and allowed mapping between the WHO drug name, WHO Drug Code, reference ID, anatomic therapeutic class (ATC), chemical abstracts service (CAS) number, unique ingredient identifier (UNII), and WHO Standard Drug Groupings (SDG) identification number. This dictionary was chosen as it was the most used drug standardization dictionary by sponsors participating in our project. By standardizing treatment information with the WHO Drug dictionary, we were not only able to work with standardized data, but also utilize the ATC and SDG classification systems to analyze the effect of broader drug classes such as corticosteroids.

Note S4: Unit Testing for Data Quality Control

Unit testing occurred before parsing and served to test if there were any underlying problems with the data. Occasionally we ran into issues where a file was either corrupt or required special parameters to open and extract data. To address this, unit tests identified datafiles for which additional effort was needed to open the file. Other unit tests ensured that patient identification numbers (contained in the USUBJID column) in various SDTM domains occur in the patient demographics table. This was important as the demographics table served as a starting point during analysis and information was often merged into this table. If patients existed in other standard tables, but not in the patient demographics table, these data would be dropped during analysis and information would be lost. The results of each unit test were added to a text file which recorded the name of the test, the date and time the test was performed, and the input source of the unit test function. This log created a searchable record of problems with newly parsed data.

Note S5: Jupyter Notebooks for ETL Quality Control

Jupyter notebooks were used after parsing but before integration in the CAR T cell safety database. Parsing notebooks allowed the testing of data extract, transform, and load (ETL) functions and identified errors that required further data management solutions. For each domain, custom functions were written to perform tasks such as looking for outliers in dosing information, identifying spelling errors in adverse event or medication terms, and checking integrity of patient identifiers across data tables. Custom parsing functions were frequently developed within the parsing notebooks as well. For example, if regular expression substitution was required to clean dose information, a function to perform this action would first be developed within the Jupyter parsing notebook. This function would then be implemented into a parsing tools library to be called during parsing. By checking the final parsing result against the function output in the parsing notebook, we could identify issues in the function implementation process.

Other examples of information derived from the parsing notebooks were missing data problems, which could impact reference table creation when multiple source files were used. If a patient had multiple product identification numbers but only one administered product, this information would cause issues when tables were merged to create a single reference table. A decision could then be made on how to handle this issue (usually the patient was dropped). Another example of the missing data problem identified by the parsing notebooks was that different companies treat death differently. Usually, the demographics table would contain information about all patients, as well as if they died. The death details table however may only contain patients that died after receiving treatment. Differences like these could be identified via parsing notebooks and allowed us to capture a more complete clinical picture.

Note S6: CRS Management Protocol Group Notes

Selection into the lower grade (LGI) vs higher grade (HGI) grade intervention group was based on the management plan described in the protocol instead of based on utilizing tocilizumab administration data for each patient. To alleviate concerns that selection based on described protocol may not coincide with actual tocilizumab treatment, we evaluated tocilizumab use in low-grade CRS to ensure that a substantial proportion of HGI subjects did not receive tocilizumab at lower grades. Among subjects who only experienced grade 1 or 2 CRS (n=633), LGI subjects received tocilizumab at a relatively high rate (38.9%, n=211/542) while HGI subjects rarely received tocilizumab (9.9%, n=9/91). As only 9.9% of HGI subjects received tocilizumab at lower grade, we decided that selection of subjects into the HGI and LGI groups solely based on the study protocol requirements was an acceptable surrogate for our exploratory analysis.

Note S7: Age Group Definitions

Adults were defined as age $\geq$ 21 years of age while pediatrics were defined as age < 21. Age cut off was defined at 21 as early studies targeting pediatric subjects included young adults in this age range.

Note S8: Multivariate Model Development

Model parameters were selected using forward selection using cross validation scores from a logistic regression estimator and selected parameters were fit using Logistic Regression with L1 regularization. Odds ratios and associated p values for significant variables were obtained from a logistic regression estimator. Parameters selected during sCRS model development included Indication, CRS intervention, vector design, temperature, IL4, IL8 and TNFA. For sNTX model development, indication, vector design, temperature, CCL2, IL8, IL1β, and potency (IFN-ɣ) were selected during parameter selection step.

Note S9: Cytokine Interpolation

Three timeseries interpolation models were developed (a mixed effects model, a statistical model, and a neural network model) and evaluated on a subset of timeseries data not used during model development. The mixed effects model was a semi-mechanistic pharmacokinetic model based on an assumed model-structure of a rapid increase in cytokine levels, followed by a rapid decline, and then a slow decline.[1] The statistical model used the Expectation-Maximization with Bootstrapping algorithm to first perform longitudinal interpolation for each cytokine, followed by cross-sectional interpolation across all cytokines. The neural network model used a radial basis function network with two interpolation layers to generate regularly sampled timeseries data.[2] The mixed effects model was developed using the software tool NONMEM; the statistical interpolation model was developed using the Amelia Package in R; and the neural network model was developed using the TensorFlow package in Python.[3-5] Three metrics were used to measure interpolation model performance: the root-mean-square deviation (RMSD), the median absolute deviation (MAD), and the percentage of predictions within a 35% confidence interval of observed values. The best performing interpolation model was then used for predictive modeling.

Supplemental Tables

Table S1: *Rates of neurotoxicity and cytokine release syndrome by vector design.* CAR T cells produced with gammaretrovirus vectors that included CD28 sequences in the CAR design had higher risk of severe neurotoxicity (NTX Grade ≥ 3) sNTX but not severe cytokine release syndrome (CRS Grade ≥ 3). Grade ≤ 2 counts includes subjects with Grade 0 (did not experience CRS or NTX).

Vector Design				Maximum NTX		Maximum CRS	
**Costimulatory Domain**	**Transmembrane**	**Hinge**	**Vector Type**	**Grade ≤ 2**	**Grade ≥ 3**	**Grade ≤ 2**	**Grade ≥ 3**
CD28	CD28	CD28	Gamma-retrovirus	65.5% (n=243)	34.5% (n=128)	83.3% (n=309)	16.7% (n=62)
CD137 (4-1BB)	CD8a	CD8a	Lentivirus	88.1 % (n=446)	11.9% (n=60)	80.0% (n=405)	20.0% (n=101)
CD137 (4-1BB)	CD28	IgG4	Lentivirus	87.4% (n=341)	12.6% (n=49)	97.2% (n=379)	2.8% (n=11)
CD28	CD28	CD28	Lentivirus	100.0% (n=10)	0% (n=0)	100.0% (n=10)	0% (n=0)

Table S2: *Differences in Cytokine Levels for Subjects with sCRS and sNTX*. Average maximum cytokines levels in subjects with severe cytokine release syndrome (sCRS) or neurological toxicities (sNTX) compared to subjects without sCRS or sNTX. For subjects who experienced sCRS, average maximum cytokine concentrations were calculated using three different time ranges after CAR T cell administration (within 36 hours, before the occurrence of sCRS, and within 28 days). For subjects who experienced sNTX, average maximum cytokine concentrations were calculated using two different time ranges after CAR T cell administration (within 36 hours and within 28 days). NSD = No Significant Difference at p=0.05.

Cytokine	sCRS (36 hours)	sCRS (Before CRS)	sCRS (28 Days)	sNTX (36 hours)	sNTX (28 Days)
CCL2	Higher (p<0.001, n=582)	Higher (p=0.01, n=787)	Higher (p=0.039, n=823)	Higher (p<0.001, n=582)	NSD
CCL3	NSD	NSD	Higher (p=0.001, n=863)	Lower (p<0.001, n=623)	NSD
CCL4	NSD	NSD	Higher (p<0.001, n=865)	Higher (p=0.04, n=627)	NSD
GMCSF	NSD	NSD	Higher (p=0.036, n=923)	NSD	Higher (p=0.036, n=923)
IFN-γ	Higher (p=0.01, n=925)	Higher (p=0.03, n=1133)	Higher (p<0.001, n=1177)	NSD	NSD
IL1β	NSD	NSD	NSD	NSD	NSD
IL2	Higher (p=0.04, n=913)	NSD	Higher (p=0.005, n=1169)	NSD	NSD
IL4	Lower (p<0.001, n=764)	Lower (p<0.001, n=1014)	NSD	Higher (p=0.014, n=764)	NSD
IL5	NSD	NSD	NSD	NSD	NSD
IL6	NSD	NSD	Higher (p<0.001, n=1178)	NSD	NSD
IL7	NSD	NSD	Higher (p=0.002, n=843)	Higher (p=0.048, n=600)	Higher (p=0.002, n=843)
IL8	Higher (p=0.001, n=925)	NSD	Higher (p<0.001, n=1178)	Higher (p=0.023, n=925)	Higher (p=0.009, n=1178)
IL10	NSD	NSD	NSD	NSD	NSD
IL12	NSD	Lower (p<0.001, n=1004)	NSD	NSD	NSD
IL13	NSD	NSD	Higher (p=0.04, n=1038)	NSD	Lower (p=0.047, n=1038)
IL15	NSD	NSD	NSD	NSD	NSD
TNFα	NSD	NSD	NSD	NSD	NSD

Table S3: *Top 10 most frequent adverse events classified under neurotoxicity*. Includes all MedDRA 20.1 preferred terms (with toxicity grade ≥ 1) under the neurologic disorders or psychiatric disorders system organ class. Percentage calculated using the total number of patients who received at least one administration of a CAR T cell product (n=1,277).

MedDRA Preferred Term	Count	Frequency (n=1,277)
Headache	392	30.8 %
Confusional state	211	16.5 %
Encephalopathy	180	14.1 %
Tremor	173	13.5 %
Dizziness	145	11.4 %
Aphasia	111	8.7 %
Insomnia	91	7.1 %
Anxiety	77	6.0 %
Somnolence	71	5.6 %
Agitation	52	4.1 %

Table S4: *Top 10 most frequent adverse events classified under severe neurotoxicity*. Includes all MedDRA 20.1 preferred terms (with toxicity grade ≥ 3) under the neurologic disorders or psychiatric disorders system organ class. Percentage calculated using the total number of patients who received at least one administration of a CAR T cell product (n=1,277).

MedDRA Preferred Term	Count	Frequency (n=1,277)
Encephalopathy	113	8.8 %
Confusional state	41	3.2 %
Aphasia	36	2.8 %
Headache	22	1.7 %
Somnolence	20	1.6 %
Agitation	16	1.3 %
Mental status changes	15	1.2 %
Seizure	14	1.1 %
Delirium	14	1.1 %
Neurotoxicity	12	0.9 %

Supplemental Figures

(A) Risk of sCRS in ALL subjects compared to NHL subjects

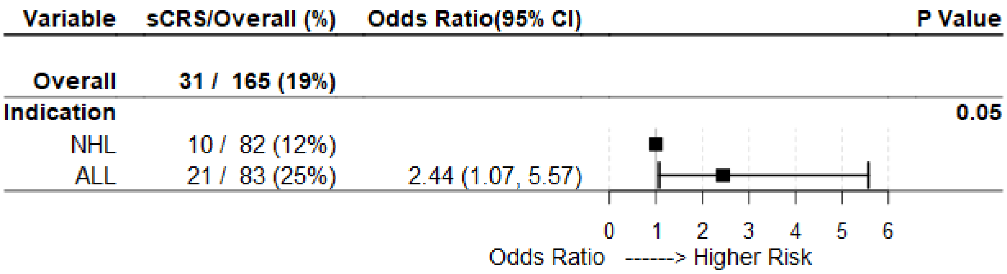

(B) Risk of sNTX in Adults with ALL vs NHL Given the Same Product

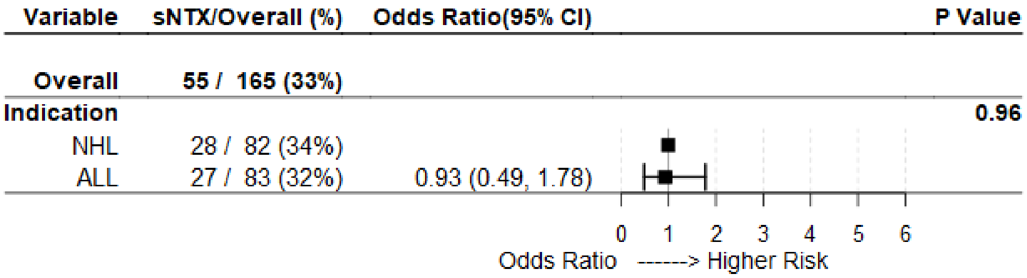

Figure S1: *Risk of sCRS and sNTX in adults with ALL vs NHL given the same product*. (A) Risk of severe CRS in ALL subjects compared to NHL subjects. (B) Risk of severe NTX in ALL subjects compared to NHL subjects. ALL = acute lymphocytic leukemia. NHL = non-Hodgkin’s lymphoma. sCRS = severe (toxicity grade ≥ 3) cytokine release syndrome. sNTX = severe (toxicity grade ≥ 3) neurological toxicities.

(A) Subgroup Analysis of Age Group and sCRS

Subgroup	Age Group	CRS3+/Overall (%)	95% CI	P Value
**CRS Intervention**				
lower grade		90 / 1019 (8%)		< 0.001
	21+ (years)	79 / 981 (8%)		
	0 to 20 (years)	11 / 38 (28%)	4.65 (2.22, 9.73)	
higher grade		84 / 258 (32%)		0.034
	21+ (years)	29 / 115 (25%)		
	0 to 20 (years)	55 / 143 (38%)	1.85 (1.08, 3.18)	
**Indication**				
MM		20 / 314 (6%)		
	21+ (years)	20 / 314 (6%)		
ALL		98 / 304 (32%)		0.064
	21+ (years)	33 / 127 (25%)		
	0 to 20 (years)	65 / 177 (36%)	1.65 (1.0, 2.73)	
NHL		56 / 659 (8%)		0.3
	21+ (years)	55 / 655 (8%)		
	0 to 20 (years)	1 / 4 (25%)	3.64 (0.37, 35.55)	
**Prior Transplant**				
Y		118 / 1075 (10%)		< 0.001
	21+ (years)	77 / 952 (8%)		
	0 to 20 (years)	41 / 123 (33%)	5.68 (3.65, 8.83)	
N		56 / 202 (27%)		0.003
	21+ (years)	31 / 144 (21%)		
	0 to 20 (years)	25 / 58 (43%)	2.76 (1.44, 5.31)	
**SEX**				
M		97 / 801 (12%)		< 0.001
	21+ (years)	62 / 703 (8%)		
	0 to 20 (years)	35 / 98 (35%)	5.74 (3.52, 9.36)	
F		77 / 476 (16%)		< 0.001
	21+ (years)	46 / 393 (11%)		
	0 to 20 (years)	31 / 83 (37%)	4.5 (2.62, 7.72)	
**Vector Design**				
non-gCD28vec		112 / 906 (12%)		< 0.001
	21+ (years)	56 / 760 (7%)		
	0 to 20 (years)	56 / 146 (38%)	7.82 (5.09, 12.03)	
gCD28vec		62 / 371 (16%)		0.082
	21+ (years)	52 / 336 (15%)		
	0 to 20 (years)	10 / 35 (28%)	2.18 (0.99, 4.82)	

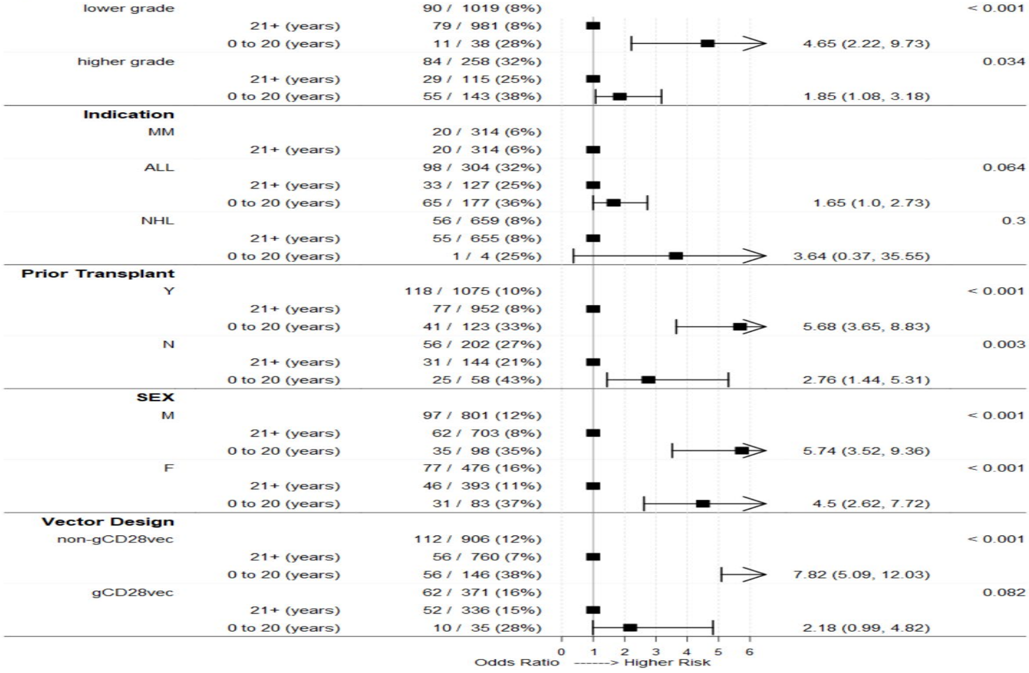

(B) Subgroup Analysis of Age Group and sNTX

Subgroup	Age Group	NTX3+/Overall (%)	95% CI	P Value
**CRS Intervention**				
lower grade		190 / 1019 (18%)		0.147
	21+ (years)	179 / 981 (18%)		
	0 to 20 (years)	11 / 38 (28%)	1.83 (0.89, 3.75)	
higher grade		47 / 258 (18%)		0.427
	21+ (years)	18 / 115 (15%)		
	0 to 20 (years)	29 / 143 (20%)	1.37 (0.72, 2.62)	
**Indication**				
MM		17 / 314 (5%)		
	21+ (years)	17 / 314 (5%)		
ALL		87 / 304 (28%)		0.002
	21+ (years)	49 / 127 (38%)		
	0 to 20 (years)	38 / 177 (21%)	0.44 (0.26, 0.72)	
NHL		133 / 659 (20%)		0.183
	21+ (years)	131 / 655 (20%)		
	0 to 20 (years)	2 / 4 (50%)	4.0 (0.56, 28.66)	
**Prior Transplant**				
Y		191 / 1075 (17%)		0.507
	21+ (years)	166 / 952 (17%)		
	0 to 20 (years)	25 / 123 (20%)	1.21 (0.75, 1.93)	
N		46 / 202 (22%)		0.632
	21+ (years)	31 / 144 (21%)		
	0 to 20 (years)	15 / 58 (25%)	1.27 (0.63, 2.58)	
**SEX**				
M		142 / 801 (17%)		0.75
	21+ (years)	123 / 703 (17%)		
	0 to 20 (years)	19 / 98 (19%)	1.13 (0.66, 1.94)	
F		95 / 476 (19%)		0.234
	21+ (years)	74 / 393 (18%)		
	0 to 20 (years)	21 / 83 (25%)	1.46 (0.84, 2.55)	
**Vector Design**				
non-gCD28vec		109 / 906 (12%)		0.001
	21+ (years)	79 / 760 (10%)		
	0 to 20 (years)	30 / 146 (20%)	2.23 (1.4, 3.55)	
gCD28vec		128 / 371 (34%)		0.556
	21+ (years)	118 / 336 (35%)		
	0 to 20 (years)	10 / 35 (28%)	0.74 (0.34, 1.59)	

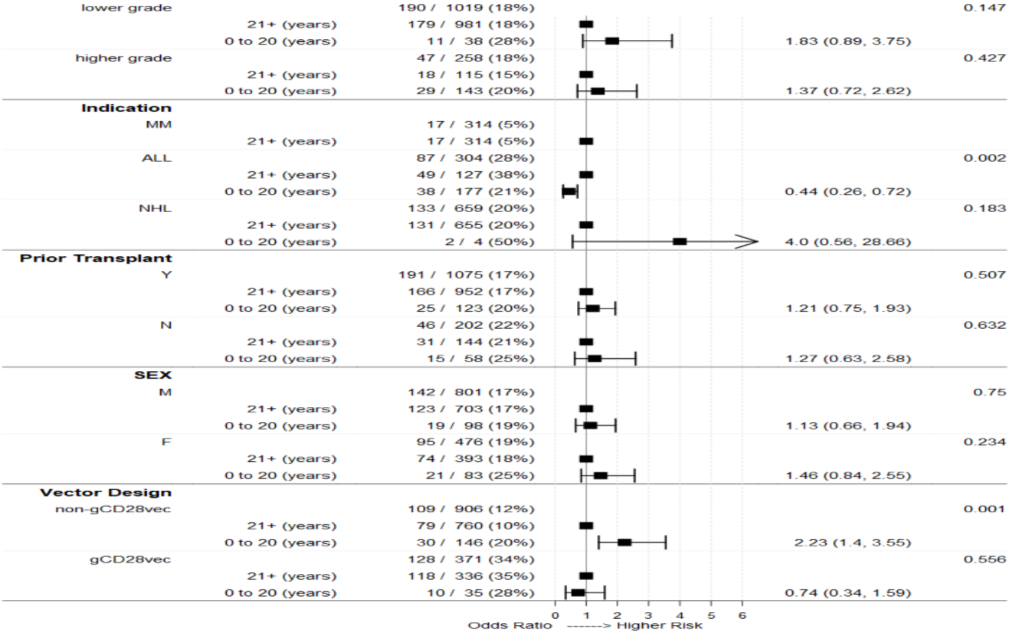

Figure S2: *Subgroup analysis of age group with sCRS and sNTX*. Within each subgroup (as defined in the first column), rates of severe (toxicity grade ≥ 3) cytokine release syndrome (sCRS) or neurological toxicities (sNTX) were calculated for adults (21+ (years)) and pediatrics (0 to 20 (years)). The fourth column contains an odds ratio and 95% confidence interval calculated using adults as a reference point within each subgroup. The fifth column contains forest plots to visually represent the odds ratio and 95% confidence interval. The sixth column contains p-values from chi-squared comparing severe toxicity rates in each group. (**A**) Subgroup analysis of age group and sCRS. (**B**) Subgroup analysis of age group and sNTX. ALL = acute lymphocytic leukemia. NHL = non-Hodgkin's lymphoma. MM = multiple myeloma. gCD28vec = products produced with gammaretroviral vectors with CD28 sequences in the transgene.

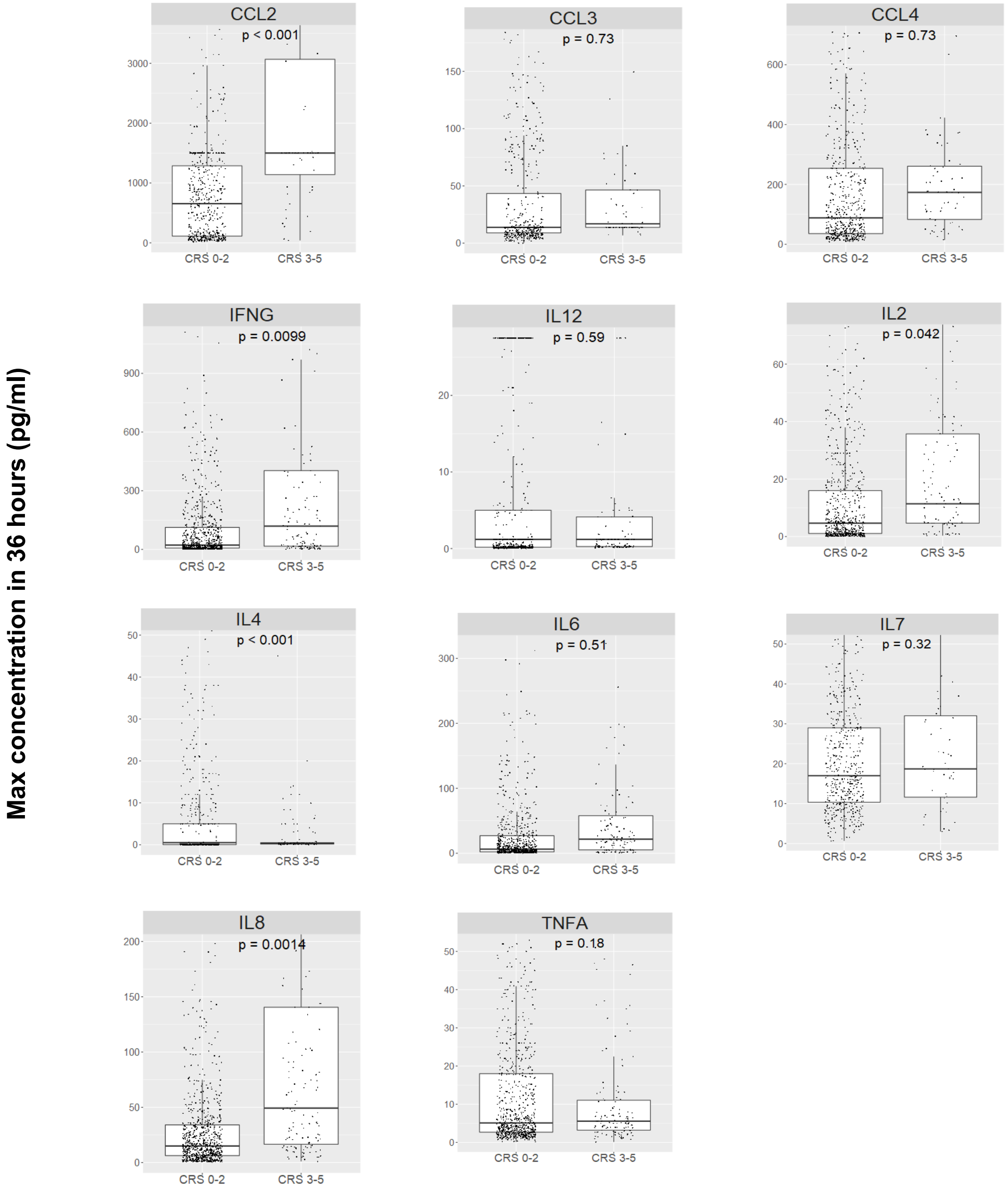

Figure S3: *Comparison of maximum cytokine concentrations between subjects with and without severe cytokine release syndrome*. Within 36 hours of CAR T cell product administration, subjects who experienced severe cytokine release syndrome (sCRS) had significantly higher maximum concentrations of IL2, CCL2, and IFN-γ and lower concentrations of IL4 compared with subjects with non-sCRS. CRS 0-2 = non-sCRS (cytokine release syndrome with toxicity grade 0, 1, or 2). CRS 3-5 = sCRS (cytokine release syndrome toxicity with toxicity grade 3, 4, or 5).

(A)

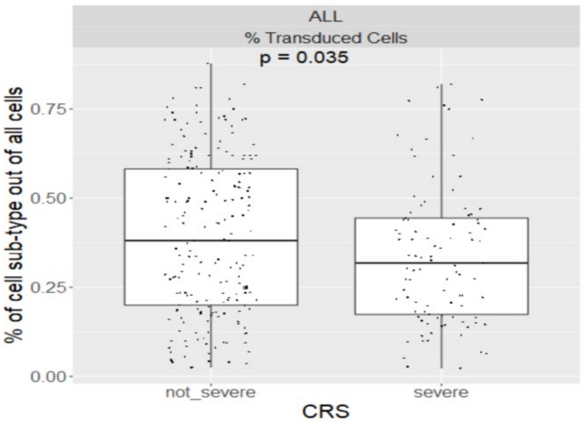

(B)

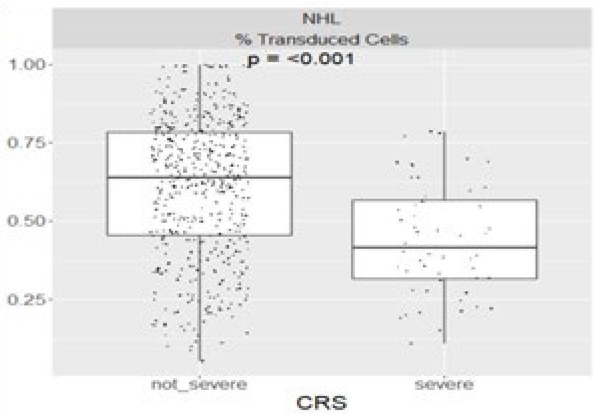

(C)

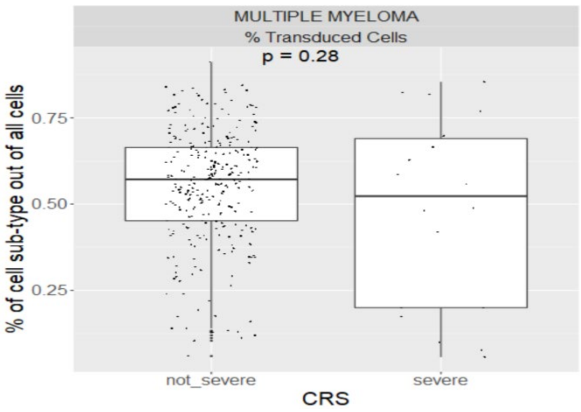

Figure S4: *Comparison of CAR T cell product transduction rates between subjects with and without sCRS by indication*. Transduction rates were lower among subjects who experienced severe (toxicity grade ≥ 3) cytokine release syndrome (sCRS) compared with subjects who did not for subjects with (A) ALL (32% vs. 38%, p=0.035, n=298) and (B) NHL (42% vs. 64%, p<0.001, n=546), but not for subjects with (C) Multiple Myeloma (52% vs. 57%, p=0.28, n=306). ALL = acute lymphocytic leukemia. NHL = non-Hodgkin’s lymphoma.

(A) Severe CRS Prediction Across Different Indications

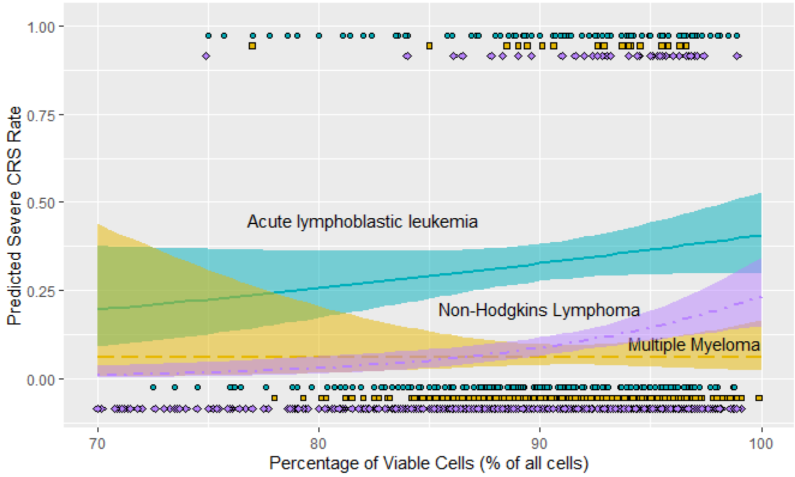

(B) Severe CRS Prediction in Different Intervention Strategies

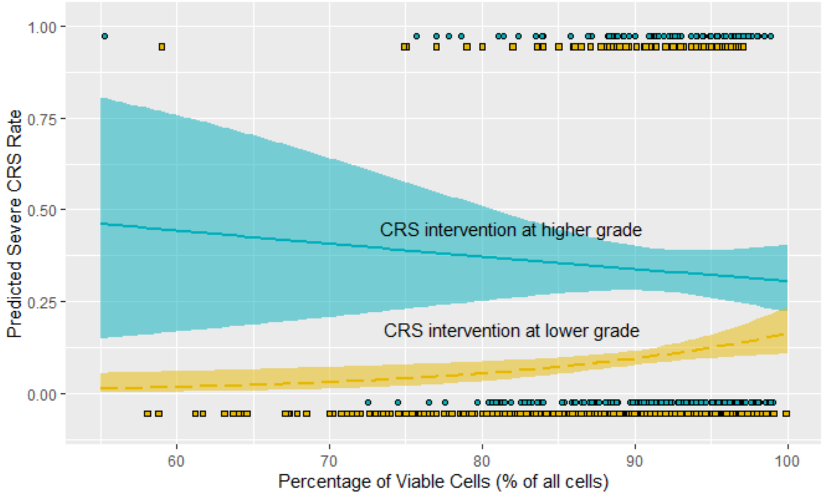

Figure S5: *Effect of Cellular composition of viable cells on the probability of sCRS*. (A) Subgroup analysis of probabilities of experiencing severe (toxicity grade ≥ 3) cytokine release syndrome (sCRS) at different levels of percentage of viable cells across different indication. Subgroup analysis by indication (underlying disease) found that among non-Hodgkin's lymphoma (NHL) subjects, percentages of viable cells were higher among subjects who experienced sCRS compared with subjects who did not (94.5%, n=52/493 vs. 89.3, n=493/545, p<0.001), although this difference was not significant in subjects with acute lymphocytic leukemia (ALL) (92.2%, n=97/298 vs. 90.0%, n=201/298, p=0.155) or multiple myeloma (MM) (92.8%, n=18/292 vs. 91.3%, n=274/292, p=0.974). (B) Subgroup analysis of probabilities of experiencing sCRS at different levels of percentage of Viable Cells across different CRS intervention groups. Subgroup analysis by CRS intervention groups found that among subjects in the lower grade intervention group, the percentage of viable cells was higher among subjects who experienced sCRS compared with subjects who did not (92.5%, n=84/886 vs. 89.8%, n=802/886, p=0.002).

Subgroup	CRS Intervention	sNTX/Overall (%)	Odds Ratio(95% CI)	P Value
**Age Group**				
Adult		49 / 127 (39%)		1
	LGI	44 / 115 (38%)		
	HGI	5 / 12 (41%)	1.15 (0.34, 3.86)	
Pediatric		38 / 177 (21%)		0.361
	LGI	10 / 35 (29%)		
	HGI	28 / 142 (19%)	0.61 (0.26, 1.42)	

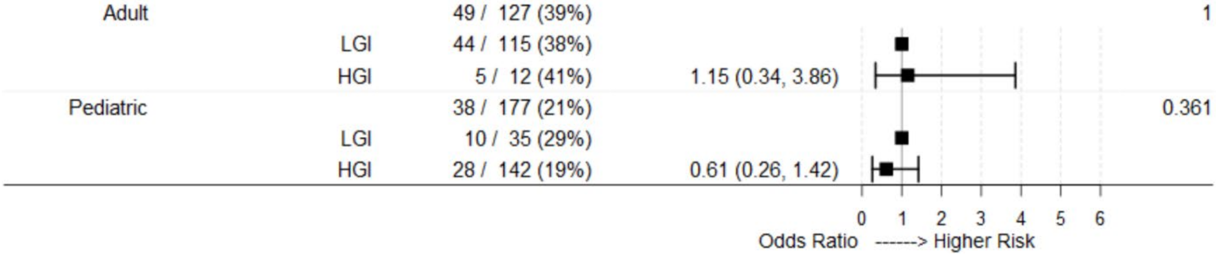

Figure S6: *Subgroup Analysis of CRS Intervention in Subjects with ALL*. For subjects with acute lymphocytic leukemia (ALL), there was no significant difference in severe (toxicity grade ≥ 3) neurological toxicity (sNTX) rates between higher grade and lower grade cytokine release syndrome (CRS) intervention strategies among either adults or pediatrics. HGI = higher grade intervention. LGI = lower grade intervention.

Subgroup	CRS Intervention	sNTX/Overall (%)	Odds Ratio(95% CI)	P Value
**Target Antigen**				
BCMA		17 / 314 (5%)		
	LGI	17 / 314 (5%)		
CD19		92 / 592 (16%)		0.143
	LGI	45 / 334 (13%)		
	HGI	47 / 258 (18%)	1.43 (0.92, 2.23)	

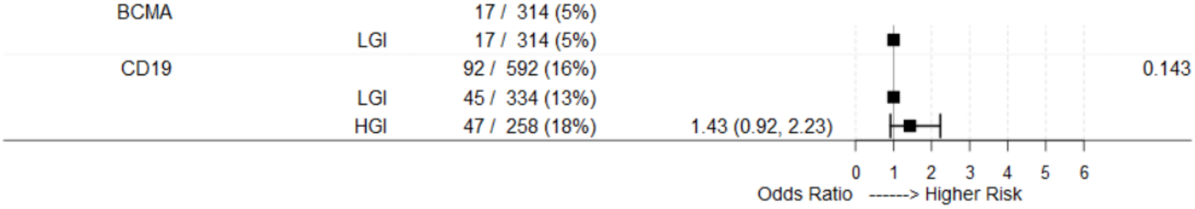

Figure S7: *Subgroup Analysis of CRS intervention in Subjects given non-gCD28vec products*. For subjects who did not receive CAR T cells with gammaretrovirus vectors containing CD28 sequences (non-gCD28vec), there was no significant difference in severe (toxicity grade ≥ 3) neurological toxicity (sNTX) rates between higher grade and lower grade cytokine release syndrome (CRS) intervention strategies among subjects who received CAR T cell products targeting CD19 antigens. For non-gCD28vec subjects, no subjects who received CAR T cell products targeting BCMA antigens were part of the higher grade intervention (HGI) group. LGI = lower grade intervention.

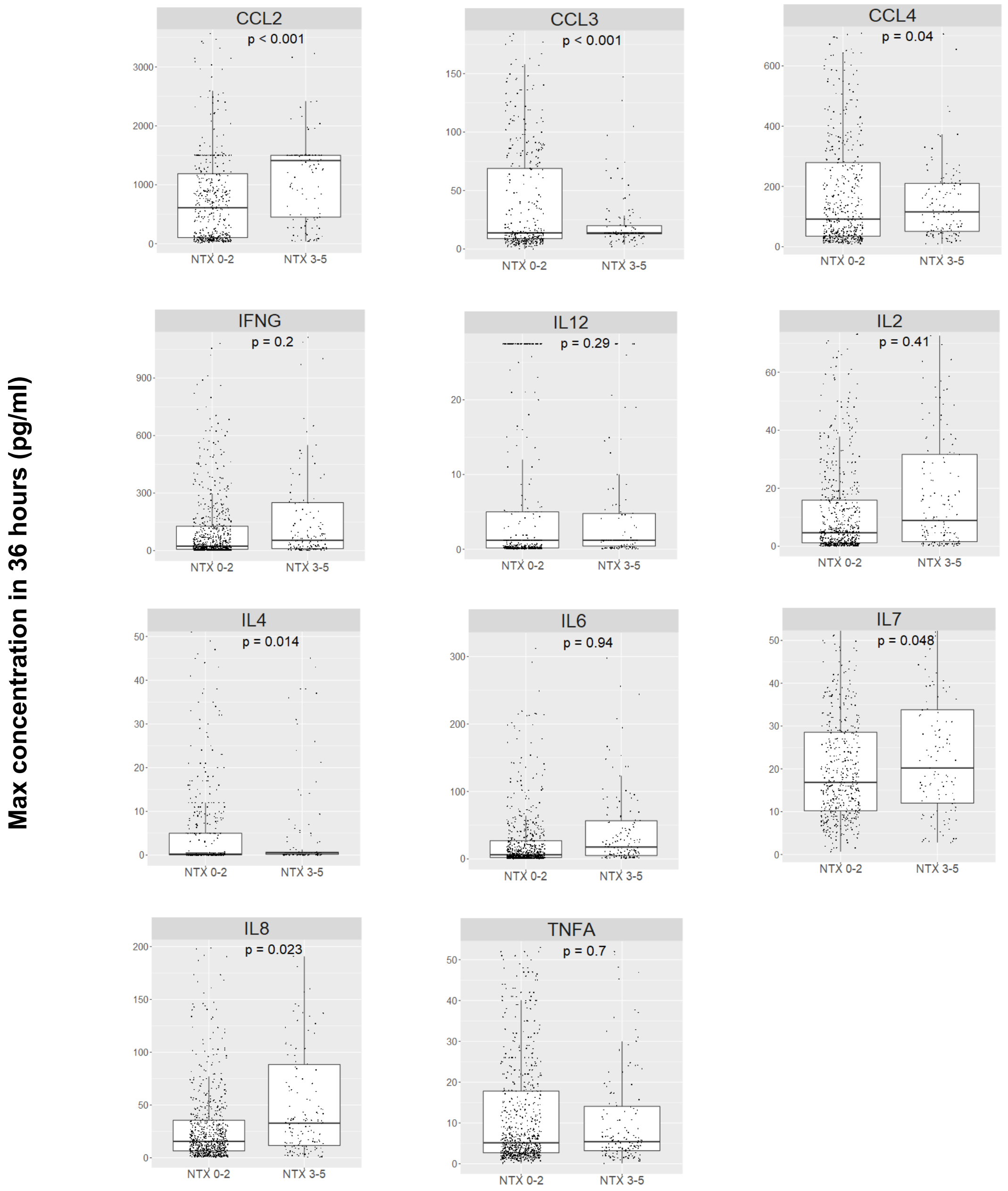

Figure S8: *Comparison of maximum cytokine concentrations between subjects with and without severe neurological toxicities***.** Within 36 hours of CAR T cell product administration, subjects who experienced severe neurological toxicities (sNTX) had significantly higher maximum concentrations of IL4, IL7, IL8, CCL2, and CCL4 and lower concentrations of CCL3 compared to subjects with non-sNTX. NTX 0-2 = non-sNTX (neurological toxicities with toxicity grade 0, 1, or 2). NTX 3-5 = sNTX (neurological toxicities with toxicity grade 3, 4, or 5).

Appendix I: CAR T Cell Data Schema

Demographics

Table Name: cart_demographics_adj

Description: Contains detailed patient information.

SDTM Domain: Demographics (DM)

Variable Name	Variable Label	Description
**USUBJID**	**Unique Subject Identifier**	Unique patient id.
**STUDYID**	**Study Identifier**	Unique study id.
**SITEID**	**Site Identifier**	Unique identifier for the site where the study occurs.
**INDID**	**IND identifier**	Investigational New Drug (IND) or Biologics License Application (BLA) number from which the data was parsed
**RFSTDTC**	**Date/Time of Initial Treatment**	CART infusion Start Date/Time. Use ISO 8601 format. All dates listed as YYYY-MM-DD T HH:MM: SS.
**RFENDTC**	**Date/Time when patient left study**	Date/Time when patient ended participation in the study. Use ISO 8601 format. All dates listed as YYYY-MM-DD T HH:MM: SS.
**BRTHDTC**	**Date/Time of Birth**	Date/Time of Birth of the subject. Use ISO 8601 format. All dates listed as YYYY-MM-DD T HH:MM: SS. Only year is parsed from patient data, all other info standardized to YYYY-01-01T00:00:00.
**AGE**	**Age**	Age of patient at the time of demographics data collection.
**AGEU**	**Age Units**	Unit for age.
**SEX**	**Sex**	Gender of patient. Use the following labels:<br>**Label** – **Description**<br>F – Female<br>M – Male
**RACE**	**Race**	Race of patient. Refer to "Collection of Race and Ethnicity Data in Clinical Trials. Guidance for Industry and Food and Drug Administration Staff" (FDA, October 2018) for guidance regarding the collection of race (https://www.fda.gov/media/75453/download).
**ETHNIC**	**Ethnicity**	Race of patient. Refer to "Collection of Race and Ethnicity Data in Clinical Trials. Guidance for Industry and Food and Drug Administration Staff" (FDA, October 2018) for guidance regarding the collection of ethnicity (https://www.fda.gov/media/75453/download).
**weight**	**Weight**	Initial weight of patient.
**weightu**	**Weight Units**	Unit for weight.
**COUNTRY**	**Country**	Country of study site.
**DMDTC**	**Date/Time of Collection**	Date of collection of demographics data.
**height**	**Height**	Initial height of patient.
**heightu**	**Height Units**	Unit for height.

<table>
<tr><td>prior_transplant</td><td>Prior Transplant</td><td>Has the patient received stem cell transplantation prior to treatment? Use the following labels:
<table><tr><th>Label</th><th>Description</th></tr><tr><td>Y</td><td>Yes</td></tr><tr><td>N</td><td>No</td></tr></table></td></tr>
<tr><td>transplant_type</td><td>Transplant Type</td><td>Type of prior_transplant. Use the following labels:
<table><tr><th>Label</th><th>Description</th></tr><tr><td>Autologous</td><td>Autologous</td></tr><tr><td>Allogeneic</td><td>Allogeneic</td></tr></table></td></tr>
</table>

Required entries:
Demographics information is required for all patients.

Non-Duplicate Key:
The following variables are used to ensure there are no duplicate data:
- USUBJID
- STUDYID

Additional Information: NA

Death Details

Table Name: DEATH_DETAILS

Description: Contains detailed information about death occurrences.

SDTM Domain: Death Details (DD)

Variable Name	Variable Label	Description
**USUBJID**	**Unique Subject Identifier**	Unique patient id.
**STUDYID**	**Study Identifier**	Unique study id.
**SITEID**	**Site Identifier**	Unique identifier for the site where the study occurs.
**DDTESTCD**	**Death Detail Assessment Short Name**	Name of assessment. See below for required values.<br>**Label**: **CAUSE_OF_DEATH** — Description: Text description of the cause of death.<br>**Label**: **AUTOPSY_FINDING** — Description: Text description of autopsy findings.
**DDTEST**	**Death Detail Assessment Name**	Long name for assessment code found in '**DDTESTCD'**.
**DDORRES**	**Result**	Result of assessment.
**DDDTC**	**Date/Time of Collection**	Date/time of assessment. Use ISO 8601 format. All dates listed as YYYY-MM-DD T HH:MM: SS.
**date_of_death**	**Date/Time of Death**	Date/time when patient was declared dead. Use ISO 8601 format. All dates listed as YYYY-MM-DD T HH:MM: SS.
**death_study_day**	**Study Day of Death**	Study Day of death derived from the 'date_of_death' (preferred) or 'DDDTC' (if 'date_of_death' not available). Calculated by subtracting the date_of_death from 'RFSTDTC' variable in the Demographics table.

Required entries: NA

Non-Duplicate Key:
The following variables are used to ensure there are no duplicate data:

- USUBJID
- STUDYID
- SITEID
- DDTEST

Additional Information: NA

Disease Identification

Table Name: DISEASE_IDENTIFICATION

Description: Contains information about the patients underlying condition and date of diagnosis.

SDTM Domain: **Disease Response (RS)**

Variable Name	Variable Label	Description
**USUBJID**	**Unique Subject Identifier**	Unique patient id.
**STUDYID**	**Study Identifier**	Unique study id.
**SITEID**	**Site Identifier**	Unique identifier for the site where the study occurs.
**indication**	**Indication**	Disease for which patients are receiving CART treatment. Use the following labels:<br>**Label** – **Description**<br>ALL – Acute lymphoblastic leukemia<br>AML – Acute myeloid leukemia<br>CLL – Chronic lymphoid leukemia<br>CML – Chronic myeloid leukemia<br>HL – Hodgkin's lymphoma<br>MM – Multiple myeloma<br>NHL – Non-Hodgkin lymphoma
**indication_cat**	**Category of Indication**	Used to identify different categories within a disease. See *Additional Information* for required categories.
**indication_scat**	**Category of Indication**	Subcategory used to identify different categories within a disease category. Use the following labels:<br>**Label** – **Description**<br>Relapsed – Relapsed<br>Refractory – Refractory<br>Relapsed/Refractory – Relapsed/Refractory
**diag_date**	**Date/Time of Diagnosis**	Date time when diagnosis was made. Use ISO 8601 format. All dates listed as YYYY-MM-DD T HH:MM: SS.
**diag_study_day**	**Study Day of Diagnosis**	Study day of diagnosis derived from the 'diag_date' variable. Calculated by subtracting the diag_date from 'RFSTDTC' variable in the Demographics table.

Required entries: NA

Non-Duplicate Key:
The following variables are used to ensure there are no duplicate data:

- USUBJID
- STUDYID
- indication_cat
- SITEID
- indication
- diag_date

Additional Information:
The following table lists indications and their corresponding list of categories. Indications with associated categories are required to have their categories listed.

indication	indication_cat
ALL	NA
AML	NA
CLL	NA
CML	NA
HL	Lymphocyte depleted
HL	Lymphocyte-rich
HL	Mixed cellularity
HL	Nodular lymphocyte predominant Hodgkin lymphoma
HL	Nodular sclerosis
HL	Other
MM	NA
NHL	B Cell (Not Otherwise Specified)
NHL	Burkitt lymphoma
NHL	Diffuse Large B cell
NHL	Follicular
NHL	Mantle Cell Lymphoma
NHL	Marginal zone
NHL	Primary Mediastinal B Cell Lymphoma

Adverse Events

Table Name: ADVERSE_EVENTS

Description: Contains a list of adverse events experienced by patients during the course of the study, or present at the beginning of the study.

SDTM Domain**: Adverse Events (AE), Clinical Events (CE)**

<table>
<tr><th>Variable Name</th><th>Variable Label</th><th>Description</th></tr>
<tr><td>USUBJID</td><td>Unique Subject Identifier</td><td>Unique patient id.</td></tr>
<tr><td>STUDYID</td><td>Study Identifier</td><td>Unique study id.</td></tr>
<tr><td>SITEID</td><td>Site Identifier</td><td>Unique identifier for the site where the study occurs.</td></tr>
<tr><td>AECAT</td><td>Category for Adverse Event</td><td>Category used to group related adverse event. Use the following labels:
<table>
<tr><th>Label</th><th>Description</th></tr>
<tr><td>PRE_TRT</td><td>Adverse event that manifests before treatment administration.</td></tr>
<tr><td>CRS</td><td>Adverse event that is attributable to treatment induced CRS (Cytokine Release Syndrome).</td></tr>
<tr><td>POST_TRT</td><td>Adverse events not including CRS that manifests during and after treatment administration.</td></tr>
<tr><td>LT</td><td>Adverse events found upon long term follow-up.</td></tr>
</table></td></tr>
<tr><td>AESCAT</td><td>Subcategory for Adverse Event</td><td>Further sub-groups within category. Use the following labels:
<table>
<tr><th>Label</th><th>Description</th></tr>
<tr><td>CRS_TOX</td><td>Cumulative toxicity of CRS event.</td></tr>
<tr><td>NEURO_TOX</td><td>Adverse event with CNS involvement.</td></tr>
<tr><td>CARDIAC_TOX</td><td>Adverse event with cardiac involvement.</td></tr>
<tr><td>RENAL_TOX</td><td>Adverse event with renal involvement.</td></tr>
<tr><td>PULMONARY_TOX</td><td>Adverse event with pulmonary involvement.</td></tr>
<tr><td>OTHER_TOX</td><td></td></tr>
</table></td></tr>
<tr><td>AESCAT_MAP</td><td>Mapped Subcategory for Adverse Event</td><td>Sub-group determined using the custom adverse event mapping function. The user selected adverse event map will determine the potential values for this variable.</td></tr>
<tr><td>AETERM</td><td>Reported Term for Adverse Event</td><td>Name of adverse event.</td></tr>
<tr><td>AEDECOD</td><td>Dictionary-Derived Term</td><td>Standardized text description of Adverse Event term using a standard dictionary such as MedDRA.</td></tr>
<tr><td>AEDECOD_MAP</td><td>Mapped Dictionary-Derived Term</td><td>Dictionary derived term derived using the adverse event reference table. The adverse event reference table was created using MedDRA 20.1. Users can map at the preferred term, lower level term, and system organ class level.</td></tr>
<tr><td>AEDICT</td><td>Adverse Event Term Dictionary</td><td>Standard used for AEDECOD (e.g. MedDRA).</td></tr>
<tr><td>AEVER</td><td>Version of Adverse Event Term Dictionary</td><td>Version of standard used for AEDECOD.</td></tr>
</table>

**AETOXGR**	**Standard Toxicity Grade**	Toxicity grade for adverse events based on given criteria.
**GRCRIT**	**Standard used for Toxicity Grade**	Standard used for grading adverse event (e.g. CTCAE).
**GRCRIVER**	**Version of Standard used for Toxicity Grade**	Version of grading standard used.
**AESTDTC**	**Start Date/Time of Adverse Event**	Date and time when adverse event began manifesting. Use ISO 8601 format. All dates listed as YYYY-MM-DD T HH:MM: SS.
**AEENDTC**	**End Date/Time of Adverse Event**	Date and time when adverse event ended due to resolution or censoring. Use ISO 8601 format. All dates listed as YYYY-MM-DD T HH:MM: SS.
**ae_study_day**	**Study Day of the Start of Adverse Event**	Study day of the start of the adverse event derived from the 'AESTDTC' variable. Calculated by subtracting the AESTDTC from 'RFSTDTC' variable in the Demographics table.
**ae_end_study_day**	**Study Day of the End of Adverse Event**	Study day of the end of the adverse event derived from the 'AEENDTC' variable. Calculated by subtracting the AEENDTC from 'RFSTDTC' variable in the Demographics table.

Required entries:

NA

Non-Duplicate Key:

The following variables are used to ensure there are no duplicate data:

- USUBJID
- SITEID
- STUDYID
- AESCAT
- AETERM
- AEDECOD
- AEDICT
- AEVER
- AETOXGR
- GRCRIT
- GRCRIVER
- AESTDTC
- AEENDTC

Additional Information:

NA

Treatment

Table Name: TREATMENT

Description: Contains a list of non-CART treatments administered to patients. Each administration should be in a separate row.

SDTM Domain: **Concomitant/Prior Medications (CM)**

<table>
<tr><th>Variable Name</th><th>Variable Label</th><th>Description</th></tr>
<tr><td>USUBJID</td><td>Unique Subject Identifier</td><td>Unique patient id.</td></tr>
<tr><td>STUDYID</td><td>Study Identifier</td><td>Unique study id.</td></tr>
<tr><td>SITEID</td><td>Site Identifier</td><td>Unique identifier for the site where the study occurs.</td></tr>
<tr><td>CMCAT</td><td>Category of treatment</td><td>Category used to group related adverse event. Use the following labels:
<table>
<tr><th>Label</th><th>Description</th></tr>
<tr><td>PRE_TRT</td><td>Treatments administered before CART administration.</td></tr>
<tr><td>POST_TRT</td><td>Treatments administered after CART administration.</td></tr>
</table></td></tr>
<tr><td>CMSCAT</td><td>Subcategory of treatment</td><td>Further sub-groups within category. Use the following labels:
<table>
<tr><th>Label</th><th>Description</th></tr>
<tr><td>OTHER_CHEMO</td><td>Chemotherapy other than Lymphodepletion.</td></tr>
<tr><td>LD</td><td>Treatments that are part of the pre-CART Lymphodepletion regiment.</td></tr>
<tr><td>CRS_MGMT</td><td>Treatments administered to manage CRS.</td></tr>
<tr><td>CONCOMITANT</td><td>All other non-CART treatments.</td></tr>
</table></td></tr>
<tr><td>CMFLEX_MAP</td><td>Flexible mapping category for Treatment</td><td>Category determined using the custom concomitant medication mapping function. Users can categorize by anatomic therapeutic class (ATC), active ingredient, chemical abstracts service (CAS) number, and unique ingredient identifier (UNII) number. The user selected adverse event map will determine the potential values for this variable.</td></tr>
<tr><td>CMTRT</td><td>Reported Name of Treatment</td><td>Name of treatment. Use generic name when possible.</td></tr>
<tr><td>CMDECOD</td><td>Standardized Name of Treatment</td><td>Standardized identification for treatment (If available).</td></tr>
<tr><td>CMDECOD_MAP</td><td>Mapped Dictionary-Derived Treatment</td><td>Dictionary derived term derived using the concomitant medication reference table. The adverse event reference table was created using the World Health Organization (WHO) B3 Drug Dictionary from March 2020.</td></tr>
<tr><td>CMDECOD_MAPID</td><td>Mapped Dictionary-Derived Treatment ID</td><td>Reference ID for the reference table derived term in CMDECOD_MAP.</td></tr>
<tr><td>CMDICT</td><td>Dictionary used for Standardized Treatment Name</td><td>Criteria used for CMDECOD (e.g. NDC).</td></tr>
</table>

**CMVER**	**Version of Dictionary used for Standardized Treatment Name**	Version of criteria used for CMDECOD.
**CMDOSE**	**Dose per Administration**	Dose per administration of treatment.
**CMDOSU**	**Dose Units**	Unit of dose (e.g. mg, mg/ml).
**CMDOSFRQ**	**Dose Frequency**	Dosing Frequency per interval (e.g. bid).
**CMROUTE**	**Route of Administration**	Route of Administration (e.g. oral, I.V.).
**CMSTDTC**	**Start Date/Time of Treatment**	Date and time when treatment administration began. Use ISO 8601 format. All dates listed as YYYY-MM-DD T HH:MM: SS.
**CMENDTC**	**End Date/Time of Treatment**	Date and time when treatment administration ended. Use ISO 8601 format. All dates listed as YYYY-MM-DD T HH:MM: SS.
**cm_study_day**	**Study Day of the Start of Treatment**	Study day of the start of the treatment derived from the 'CMSTDTC' variable. Calculated by subtracting the CMSTDTC from 'RFSTDTC' variable in the Demographics table.
**cm_end_study _day**	**Study Day of the End of Treatment**	Study day of the end of the treatment derived from the 'CMENDTC' variable. Calculated by subtracting the CMENDTC from 'RFSTDTC' variable in the Demographics table.

Required entries:

NA

Non-Duplicate Key:

The following variables are used to ensure there are no duplicate data:

- USUBJID
- CMSCAT
- CMVER
- CMROUTE
- SITEID
- CMTRT
- CMDOSE
- CMSTDTC
- STUDYID
- CMDECOD
- CMDOSU
- CMENDTC
- CMCAT
- CMDICT
- CMDOSFRQ

Additional Information:

NA

CAR T Treatment

Table Name: CART_TRT

Description: Contains a list of CART infusions administered to patients. Each infusion should be in a separate row.

SDTM Domain: **Exposure (EX), Exposure as Collected (EC)**

Variable Name	Variable Label	Description
**USUBJID**	**Unique Subject Identifier**	Unique patient id.
**STUDYID**	**Study Identifier**	Unique study id.
**SITEID**	**Site Identifier**	Unique identifier for the site where the study occurs.
**PRODUCTID**	**Product Identifier**	Unique Product Identifier.
**dose_num**	**Dose Number**	Each dose administered to a patient is sequentially numbered starting from 1.
**infusion_num**	**Infusion Number**	For each dose, each infusion (split) is sequentially numbered starting from 1.
**trans_cell_cnt**	**Transduced Cell Count**	Total transduced cell count.
**nucl_cell_cnt**	**Nucleated Cell Count**	Total nucleated cell count.
**start_date_time**	**Start Date/Time of Treatment**	Date and time when treatment administration began. Use ISO 8601 format. All dates listed as YYYY-MM-DD T HH:MM: SS.
**infusion_study_day**	**Study day of Treatment**	Study day of CART product administration derived from the 'start_date_time' variable. Calculated by subtracting the start_date_time from the 'RFSTDTC' variable in the Demographics table.

Required entries:
All infusions are required to be entered into this table.

Non-Duplicate Key:
The following variables are used to ensure there are no duplicate data:

- USUBJID
- STUDYID
- infusion_num
- SITEID
- dose_num
- start_date_time

Additional Information: NA

Lab Results

Table Name: LAB_RESULTS

Description: Contains information related to cytokine level measurements, persistence of CAR T cells after administration, temperature data, and other laboratory findings.

SDTM Domain: **Laboratory Test Results (LB), Vital Signs (VS), Pharmacokinetic Concentrations (PC)**

<table>
<tr><th>Variable Name</th><th>Variable Label</th><th>Description</th></tr>
<tr><td>USUBJID</td><td>Unique Subject Identifier</td><td>Unique patient id.</td></tr>
<tr><td>STUDYID</td><td>Study Identifier</td><td>Unique study id.</td></tr>
<tr><td>SITEID</td><td>Site Identifier</td><td>Unique identifier for the site where the study occurs.</td></tr>
<tr><td>LABCAT</td><td>Category of Lab Test</td><td>Category for lab test data. Use the following labels:
<table>
<tr><th>Label</th><th>Description</th></tr>
<tr><td>CYTOKINE</td><td>Cytokine measurements from blood</td></tr>
<tr><td>CYTOKINE_CSF</td><td>Cytokine measurements from cerebrospinal fluid</td></tr>
<tr><td>LAB</td><td></td></tr>
<tr><td>PERSISTENCE</td><td>Persistence measurements from blood</td></tr>
<tr><td>PERSISTENCE_BM</td><td>Persistence measurements from bone marrow</td></tr>
<tr><td>TEMP</td><td>Temperature data in degrees Celsius (˚C)</td></tr>
</table></td></tr>
<tr><td>LBTEST</td><td>Lab Test</td><td>Name of laboratory test being measured.</td></tr>
<tr><td>LBTEST_MAPPED</td><td>Mapped Lab Test</td><td>Dictionary derived term derived using the cytokine reference table.</td></tr>
<tr><td>LBORRES</td><td>Amount of Cytokine</td><td>Result of the laboratory test</td></tr>
<tr><td>LBORRESU</td><td>Unit for Amount of Cytokine</td><td>Unit for the result of the laboratory test.</td></tr>
<tr><td>LBDTC</td><td>Date/time of Sample Collection</td><td>Date/time of sample collection. Use ISO 8601 format. All dates listed as YYYY-MM-DD T HH:MM: SS.</td></tr>
<tr><td>lab_study_day</td><td>Study Day of Sample Collection</td><td>Study day of lab test derived from the ‘LBDTC’ variable. Calculated by subtracting the LBDTC from the ‘RFSTDTC’ variable in the Demographics table</td></tr>
<tr><td>lab_type</td><td>Lab type</td><td>Name of laboratory where test was conducted.</td></tr>
</table>

Required entries:

All measured cytokines, persistence of CAR T cells, and temperature data are required to be entered into this table. Other laboratory information may or may not be included.

Non-Duplicate Key:
The following variables are used to ensure there are no duplicate data:

- USUBJID
- STUDYID
- LBCAT
- LBTEST
- LBORRES
- LBORRESU
- LBDTC
- lab_type

Additional Information: NA

Disease Status

Table Name: DISEASE_STATUS

Description: Contains information about the status of disease at different points of assessment. If available, disease related pre-study assessments should be placed in this table.

SDTM Domain: **Disease Response (RS)**

Variable Name	Variable Label	Description
**USUBJID**	**Unique Subject Identifier**	Unique patient id.
**STUDYID**	**Study Identifier**	Unique study id.
**SITEID**	**Site Identifier**	Unique identifier for the site where the study occurs.
**RSTESTCD**	**Response Assessment Short Name**	Short name of disease assessment test. See below for required assessments.
**RSTEST**	**Response Assessment Name**	Verbatim name of the response assessment.
**RSTEST_MAPPED**	**Mapped Response Assessment Name**	Dictionary derived term derived using the disease response reference table.
**location**	**Anatomical Location of Assessment**	Anatomical location of assessment. See below for required locations.
**criteria**	**Criteria used for Response Assessment Result**	Criteria used for assessment. See below for required criteria.
**RSORRES**	**Response Assessment Result**	Result of assessment.
**RSDTC**	**Date/Time of Response Assessment**	Date time when assessment was made. Use ISO 8601 format. All dates listed as YYYY-MM-DD T HH:MM: SS.
**rs_study_day**	**Study Day of Response Assessment**	Study day of response assessment derived from the ‘RSDTC’ variable. Calculated by subtracting the RSDTC from the ‘RFSTDTC’ variable in the Demographics table.

Required entries: NA

Non-Duplicate Key:
The following variables are used to ensure there are no duplicate data:

- USUBJID
- STUDYID
- RSTESTCD
- RSTEST
- RSORRES
- location
- RSDTC
- criteria

Additional Information: NA

Medical History

Table Name: MEDICAL_HISTORY

Description: Contains patient's medical history information.

SDTM Domain: **Medical History (MH)**

Variable Name	Variable Label	Description
**USUBJID**	**Unique Subject Identifier**	Unique patient id.
**STUDYID**	**Study Identifier**	Unique study id.
**SITEID**	**Site Identifier**	Unique identifier for the site where the study occurs.
**MHTERM**	**Reported Term**	Term for describing medical condition.
**MHDECOD**	**Dictionary-Derived Term**	Dictionary derived text description of term. Equivalent to PT in MedDRA. Dictionary name and version should be provided in **term_std** and **term_std_ver**.
**term_std**	**Dictionary used for Dictionary-Derived Term**	Dictionary used for Medical History term (e.g. MedDRA).
**term_std _ver**	**Version of Dictionary used for Dictionary-Derived Term**	Version of Dictionary used for Medical History term.
**MHTOXGR**	**Medical History Toxicity Grade**	Standard Toxicity Grade for Medical History term.
**GRCRIT**	**Toxicity Grading Criteria**	Grading criteria used for Medical History term toxicity grade.
**GRCRIVER**	**Toxicity Grading Criteria Version**	Version of grading criteria used for Medical History term toxicity grade.
**MHSTDTC**	**Start Date/Time of Medical History Event**	Date of diagnosis / date of event. Use ISO 8601 format. All dates listed as YYYY-MM-DD T HH:MM: SS.
**MHENDTC**	**End Date/Time of Medical History Event**	Date of resolution / end date of event. Use ISO 8601 format. All dates listed as YYYY-MM-DD T HH:MM: SS.
**mh_study_day**	**Study day of Start of Medical History Event**	Study day of medical history start date derived from the 'MHSTDTC' variable. Calculated by subtracting the MHSTDTC from the 'RFSTDTC' variable in the Demographics table.
**mh_end_study_day**	**Study Day of Medical History Event**	Study day of medical history end date derived from the 'MHENDTC' variable. Calculated by subtracting the MHENDTC from the 'RFSTDTC' variable in the Demographics table.
**MHONGO**	**Medical History Ongoing**	Binary variable to determine if medical history event is ongoing at start of the study period.

Required entries:

All disease related pre-study medical history is required. This includes date of onset and anatomical location of each relapse. For example, we are interested in knowing if the patient had a prior history of CNS relapse pre-study, whether in remission or not.

Non-Duplicate Key:
The following variables are used to ensure there are no duplicate data:

- USUBJID
- STUDYID
- MHTERM
- MHDECOD
- term_std
- term_std_ver
- GRCRIT
- GRCRIVER
- MHSTDTC
- MHONGO

Additional Information: NA

Product

Table Name: PRODUCT

Description: Contains detailed product information.

SDTM Domain: Custom

Variable Name	Variable Label	Description
**USUBJID**	**Unique Subject Identifier**	Unique patient id.
**STUDYID**	**Study Identifier**	Unique study id.
**SITEID**	**Site Identifier**	Unique identifier for the site where the study occurs.
**product_id**	**Product Id**	Unique identifier for the product.
**product_name**	**Product Name**	Name of the product.
**PRODCAT**	**Product Category**	Type of Product (e.g. CD4, CD8, Mixed)
**donor_type**	**Donor Type**	Source of CART cells (e.g. Use Autologous or Allogeneic)
**VECID**	**Vector Identifier**	Unique Product Vector Identifier
**VECLOTID**	**Vector Lot Identifier**	Unique Product Vector Lot Identifier
**MANUFDT**	**Manufacturing Date**	Date Product Manufacturing was completed. Use ISO 8601 format. All dates listed as YYYY-MM-DD T HH:MM: SS.
**prod_manuf_study_day**	**Study Day of Manufacturing Date**	Study day of manufacturing date derived from the ‘MANUFDT’ variable. Calculated by subtracting the MANUFDT from the ‘RFSTDTC’ variable in the Demographics table.

Required entries: NA.

Non-Duplicate Key:
The following variables are used to ensure there are no duplicate data:

- PRODUCTID

Additional Information: NA

Cell Product

Table Name: CELL_PRODUCT

Description: Descriptions or measurements of CAR T cell product attributes typically contained in the product Certificate of Analysis. Example attributes include appearance, measures of potency, vector copy number (VCN), and off target rate (for genome edited products).

SDTM Domain: Custom

Variable Name	Variable Label	Description
**PRODUCTID**	**Unique Product Identifier**	Unique Product Identifier.
**CLPRDCAT**	**Category of Cell Product attribute**	Category used to group related cell product attributes.
**CLPRDTEST**	**Name of cell product attribute**	Name of cell product attribute.
**CLPRDORRES**	**Result of cell product attribute assessment**	Result of cell product attribute assessment.
**CLPRDORRESU**	**Unit of assessment**	Unit of assessment.
**CLPRDDTC**	**Date/Time of Assessment**	Date/Time of Assessment. Use ISO 8601 format. All dates listed as YYYY-MM-DD T HH:MM: SS.
**clprd_study_day**	**Study Day of Date of Assessment**	Study day of date of assessment derived from the 'CLPRDDTC' variable. Calculated by subtracting the CLPRDDTC from the 'RFSTDTC' variable in the Demographics table.

Required entries: NA

Non-Duplicate Key:
The following variables are used to ensure there are no duplicate data:

- PRODUCTID
- CLPRDTEST

Additional Information: NA

Cell Components

Table Name: CELL_COMPONENTS

Description: Measurements of cellular constituents during product manufacturing. Cell measurements at any point during manufacturing (including during cell expansion and in the final product) should be included in this table. Cell types and associated surface markers should be indicated in the CELL and MARKER columns. The type of measurements, for example cell count or percentage of cells, should be indicated in the CLCPTEST column. If the measurement was performed on a subset of cell types, this parent cell population should be identified using the DENOM_CELL and DENOM_MARKER columns. For cell expansion, measurements of different cell types, including CAR+ cells, from any in-process time point should be included in this table. Measurements at the start of expansion, as well as at harvest time, should also be included. Mean and median fluorescence for the CAR+ population should also be included in this table.

SDTM Domain: Custom.

Variable Name	Variable Label	Description
**PRODUCTID**	**Unique Product Identifier**	Unique Product Identifier.
**CLCMPCAT**	**Stage of manufacturing**	Stage of manufacturing when measurements of cellular constituents were made (e.g. Release, Apheresis, Cryopreserved).
**CELL**	**Cell Type**	Cell type being measured.
**CELLID**	**Cell Type Identifier**	Identification number from the cell type standard table for the cell type being measured.
**STD**	**Standard**	Standard dictionary from the cell type standard table for the cell type being measured.
**MARKER**	**Cell Marker**	Cell surface markers used for staining.
**MARKERID**	**Cell Marker Identifier**	Identification number from the cell marker standard table for the cell marker being measured.
**CLCMPTEST**	**Test type**	The type of measurement performed (count, percentage, volume, mean/median fluorescent intensity etc.).
**DENOM_CELL**	**Denominator cell type**	Parent cell population from which measurement was taken (if any).
**DENOM_CELLID**	**Denominator cell type Identifier**	Identification number from the cell type standard table for the parent cell population from which measurement was taken (if any).
**DENOM_MARKER**	**Denominator markers**	Cell surface markers of parent cell population (if any).
**DENOM_MARKERID**	**Denominator markers Identifier**	Identification number from the cell marker standard table for the cell surface markers of parent cell population (if any).
**CLCMPORRES**	**Result of cell component measurement**	Result of cell component measurement.
**CLCMPORRESU**	**Unit of cell component measurement**	Unit of cell component measurement.
**METHOD**	**Method of measurement**	Method used for cell component measurement.
**CLCMPSTDTC**	**Date/Time of measurement**	Date/Time of measurement. Use ISO 8601 format. All dates listed as YYYY-MM-DD T HH:MM: SS.
**clcmp_study_day**	**Study Day of Date/Time of measurement**	Study day of date of measurement derived from the 'CLCMPSTDTC' variable. Calculated by subtracting the

		CLCMPSTDTC from the ‘RFSTDTC’ variable in the Demographics table.

Required entries: NA

Non-Duplicate Key:
The following variables are used to ensure there are no duplicate data:

- PRODUCTID
- CLCMPCAT
- CELLID
- STD
- MARKERID
- CLCMPTEST
- DENOM_CELLID
- DENOM_MARKERID
- CLCMPORRESU
- METHOD
- CLCMPSTDTC

Additional Information: NA

Vector

Table Name: VECTOR

Description: Table describing the design of the vector encoding the CAR transgene. Example attributes include vector type (lentivirus, plasmid, etc.), backbone, costimulatory domain, and svFc clone.

SDTM Domain: Custom.

Variable Name	Variable Label	Description
**VECID**	**Unique vector identifier**	Unique vector identifier.
**VECNAME**	**Name of Vector**	Name of the vector
**VECTEST**	**Name of vector attribute**	Name of vector attribute being described or measured (e.g., vector type, backbone, costimulatory domain, antigen species).
**VECORRES**	**Result of vector assessment**	Result of vector assessment.
**VECORRESU**	**Unit of assessment**	Unit of assessment if relevant.
**VECSTDTC**	**Date/Time of Assessment**	Date/Time of Assessment. Use ISO 8601 format. All dates listed as YYYY-MM-DD T HH:MM: SS.

Required entries: NA

Non-Duplicate Key:
The following variables are used to ensure there are no duplicate data:

- VECNAME
- VECTEST

Additional Information: NA

Vector Lot

Table Name: VECTORLOT

Description: Table describing lot release information for the CAR transgene vector. Attributes of the vector lot which are typically contained in the Certificate of Analysis should be included in this table. Example attributes include transducing units (TU), and residual host cell DNA+.

SDTM Domain: Custom.

Variable Name	Variable Label	Description
**VECLOTID**	**Unique vector lot identifier**	Unique vector lot identifier.
**VECLOTNAME**	**Vector Lot Name**	Name of the vector lot.
**VECLOTTEST**	**Name of vector lot attribute**	Name of vector lot attribute being described or measured.
**VECLOTORRES**	**Result of vector lot assessment**	Result of vector lot assessment.
**VECLOTORRESU**	**Unit of assessment**	Unit of assessment.
**VECLOTSTDTC**	**Date/Time of Assessment**	Date/Time of Assessment. Use ISO 8601 format. All dates listed as YYYY-MM-DD T HH:MM: SS.

Required entries: NA

Non-Duplicate Key:
The following variables are used to ensure there are no duplicate data:

- VECLOTNAME
- VECLOTTEST

Additional Information: NA

Genome Editing Tool

Table Name: GENOME_EDITING_TOOL

Description: Table describing the design of the agent used to edit the T cell genome. This table should be filled if genome editing was performed on the product other than the transfer of the CAR transgene. The table should include the attributes of the genome editing tools including the genome target, the components of the genome editing tool, the mechanism by which the tools are introduced into the cells (e.g., electroporation, AAV vector) and the DNA repair pathway (e.g. NHEJ, HDR) being utilized for the given target.

SDTM Domain: Custom.

Variable Name	Variable Label	Description
**GEID**	**Unique identifier for gene editing agent**	Unique identifier for gene editing agent.
**GECAT**	**Category of gene editing agent**	Category of gene editing agent:<br>**Category**<br>TALEN<br>ZFN<br>CRISPR-CAS
**GETEST**	**Name of measured/assessed attribute**	Name of measured/assessed attribute.
**PRODUCTID**	**Unique Product Identifier**	Unique Product Identifier.
**GEORRES**	**Result of assessment**	Result of assessment
**GEORRESU**	**Unit of assessment**	Unit of assessment.

Required entries: NA

Non-Duplicate Key: NA

Additional Information: NA

Supplemental References

1. Stein, A.M., S.A. Grupp, J.E. Levine, T.W. Laetsch, M.A. Pulsipher, M.W. Boyer, K.J. August, B.L. Levine, L. Tomassian, S. Shah, et al., *Tisagenlecleucel Model-Based Cellular Kinetic Analysis of Chimeric Antigen Receptor-T Cells.* CPT Pharmacometrics Syst Pharmacol, 2019. **8**(5): p. 285-295.
2. Shukla, S.N. and B.M. Marlin, *Modeling Irregularly Sampled Clinical Time Series.* arXiv preprint arXiv:1812.00531, 2018.
3. Beal, S., L. Sheiner, A. Boeckmann, and R. Bauer, *NONMEM 7.4 users guides.* ICON plc, Gaithersburg, MD, 1989. **2018**.
4. Honaker, J., G. King, and M. Blackwell, *Amelia II: A program for missing data.* Journal of statistical software, 2011. **45**(7): p. 1-47.
5. Pedregosa, F., G. Varoquaux, A. Gramfort, V. Michel, B. Thirion, O. Grisel, M. Blondel, P. Prettenhofer, R. Weiss, and V. Dubourg, *Scikit-learn: Machine learning in Python.* the Journal of machine Learning research, 2011. **12**: p. 2825-2830.
